# Advancements in Hydrogels: A Comprehensive Review of Natural and Synthetic Innovations for Biomedical Applications

**DOI:** 10.3390/polym17152026

**Published:** 2025-07-24

**Authors:** Adina-Elena Segneanu, Ludovic Everard Bejenaru, Cornelia Bejenaru, Antonia Blendea, George Dan Mogoşanu, Andrei Biţă, Eugen Radu Boia

**Affiliations:** 1 Department of Chemistry, Institute for Advanced Environmental Research, West University of Timişoara (ICAM–WUT), 4 Oituz Street, 300086 Timişoara, Romania; adina.segneanu@e-uvt.ro; 2 Drug Research Center, Faculty of Pharmacy, University of Medicine and Pharmacy of Craiova, 2 Petru Rareş Street, 200349 Craiova, Romania; ludovic.bejenaru@umfcv.ro (L.E.B.); antonia.radu@umfcv.ro (A.B.); george.mogosanu@umfcv.ro (G.D.M.); andreibita@gmail.com (A.B.); 3 Department of Pharmacognosy & Phytotherapy, Faculty of Pharmacy, University of Medicine and Pharmacy of Craiova, 2 Petru Rareş Street, 200349 Craiova, Romania; 4 Department of Pharmaceutical Botany, Faculty of Pharmacy, University of Medicine and Pharmacy of Craiova, 2 Petru Rareş Street, 200349 Craiova, Romania; 5 Department of Ear, Nose, and Throat, Faculty of Medicine, Victor Babeş University of Medicine and Pharmacy Timişoara, 2 Eftimie Murgu Square, 300041 Timişoara, Romania; eugen.boia@umft.ro

**Keywords:** hydrogels, polymers, biomedical applications, drug delivery systems, regenerative medicine

## Abstract

In the rapidly evolving field of biomedical engineering, hydrogels have emerged as highly versatile biomaterials that bridge biology and technology through their high water content, exceptional biocompatibility, and tunable mechanical properties. This review provides an integrated overview of both natural and synthetic hydrogels, examining their structural properties, fabrication methods, and broad biomedical applications, including drug delivery systems, tissue engineering, wound healing, and regenerative medicine. Natural hydrogels derived from sources such as alginate, gelatin, and chitosan are highlighted for their biodegradability and biocompatibility, though often limited by poor mechanical strength and batch variability. Conversely, synthetic hydrogels offer precise control over physical and chemical characteristics via advanced polymer chemistry, enabling customization for specific biomedical functions, yet may present challenges related to bioactivity and degradability. The review also explores intelligent hydrogel systems with stimuli-responsive and bioactive functionalities, emphasizing their role in next-generation healthcare solutions. In modern medicine, temperature-, pH-, enzyme-, light-, electric field-, magnetic field-, and glucose-responsive hydrogels are among the most promising “smart materials”. Their ability to respond to biological signals makes them uniquely suited for next-generation therapeutics, from responsive drug systems to adaptive tissue scaffolds. Key challenges such as scalability, clinical translation, and regulatory approval are discussed, underscoring the need for interdisciplinary collaboration and continued innovation. Overall, this review fosters a comprehensive understanding of hydrogel technologies and their transformative potential in enhancing patient care through advanced, adaptable, and responsive biomaterial systems.

## 1. Introduction

### 1.1. Overview

Gels are soft, viscoelastic materials composed of three-dimensional (3D) crosslinked polymeric networks swollen with a solvent, which can be either aqueous or non-aqueous. Their hybrid nature, exhibiting both solid-like and liquid-like behavior, along with high porosity and tunable mechanical properties, renders them suitable for a broad spectrum of applications. These include biomedical applications (e.g., drug delivery systems (DDS), tissue engineering (TE) scaffolds, wound dressings, biosensors), agriculture (e.g., water-retentive soil conditioners, controlled-release fertilizers), environmental remediation (e.g., pollutant adsorption, slow-release agents), and use in food and cosmetic formulations (e.g., thickeners, stabilizers, and encapsulating systems for active ingredients) [1,2,3,4,5,6].

A prominent subclass of these materials, hydrogels, are defined as hydrophilic polymeric networks capable of absorbing and retaining large quantities of water or biological fluids while maintaining structural integrity [1]. Their network architecture is stabilized through covalent (chemical) or non-covalent (physical) crosslinking and is typically enriched with hydrophilic functional groups such as hydroxyl (–OH), carboxyl (–COOH), amide (–CONH_2_), sulfonic acid (–SO_3_H), and amino (–NH_2_). These moieties facilitate water uptake and contribute to the physicochemical versatility of hydrogels [3,6,7,8,9,10,11,12].

Hydrogels garnered considerable interest across multiple fields, including environmental protection, agriculture, and especially biomedical engineering (BME), due to their ability to closely mimic the extracellular matrix (ECM), support cellular interactions, and offer customizable mechanical and biochemical environments [3,13,14,15,16,17,18,19,20].

Biocompatibility, high water content, viscoelasticity, and responsiveness to stimuli such as pH, temperature, or ionic strength, enable precise control over hydrogels behavior in physiological conditions. As a result, they are extensively utilized in DDS, wound healing matrices, implantable medical devices, and scaffolds for TE and regenerative medicine (RM) [3,13,14,15,16,17,18,19,20]. Their adaptability continues to position them at the forefront of material innovation in both therapeutic and diagnostic domains. Crosslinking agents and techniques not only dictate the degree of network swelling and mechanical strength but also modulate biodegradability, adhesiveness, and responsiveness to environmental cues, features critical for targeted and controlled therapeutic functions [3,21,22,23].

Since their initial development by Wichterle and Lím in the 1960s for ophthalmic use in soft contact lenses, hydrogels have undergone profound technological evolution [9,15,24,25,26,27,28]. Hybrid hydrogels that integrate natural and synthetic components are increasingly being developed to harness the synergistic advantages of both material classes, enabling customization for specific therapeutic needs [16,29,30,31,32].

A significant advancement in the field is the emergence of stimuli-responsive (“smart”) hydrogels, which undergo reversible changes in their physical or chemical properties in response to external stimuli such as temperature, pH, ionic strength, light, or biomolecular signals. These “smart” systems have transformed the landscape of drug delivery, enabling spatio-temporally controlled release and reducing off-target effects. Moreover, their dynamic behavior enhances cell–matrix interactions and functional integration in TE constructs [33,34,35,36,37,38,39,40]. Parallel progress in polymer chemistry and nanotechnology has facilitated the incorporation of bioactive peptides, nanoparticles (NPs), and supramolecular architectures into hydrogel matrices, further augmenting their mechanical properties, bioadhesiveness, and degradation profiles for enhanced clinical efficacy [16,20,41,42,43].

Hydrogels can be systematically classified based on a range of criteria including their composition (natural, synthetic, hybrid), ionic nature (anionic, cationic, neutral, ampholytic), polymerization strategies (homopolymeric, copolymeric, interpenetrating networks), structural organization (amorphous, semicrystalline, crystalline), crosslinking mechanisms (chemical vs. physical), and physical form (bulk matrices, films, microspheres, NPs). Each of these characteristics critically influences the hydrogel’s interaction with biological systems and its suitability for specific applications such as injectable scaffolds, wound dressings, biosensors, or nanocarriers for targeted drug delivery [7,8,9,10,15,17,44,45].

### 1.2. Advanced Fabrication Techniques

Four-dimensional (4D) bioprinting combines 3D bioprinting, smart materials, and time-dependent transformation to produce living, dynamic, and responsive tissue-like constructs. 4D bioprinting adds time as a factor, when structures change their shape, behavior, or function over time in response to external stimuli such as temperature, pH, light, moisture, electric or magnetic fields. Hydrogels as the core bioinks are ideal for 4D bioprinting due to high water content (mimics native tissue), biocompatibility, tunable mechanical and chemical properties, responsiveness to stimuli: e.g., thermoresponsive (Pluronic, poly(*N*-isopropylacrylamide) (PNIPAAm)), changes shape with temperature; pH-responsive (e.g., chitosan, alginate blends), swell/shrink in gastrointestinal (GI) tract or wound microenvironments; light-responsive (poly(ethylene glycol) (PEG) diacrylate (PEGDA)), gelatin–methacrylate (GelMA)), with controlled degradation, crosslinking, or shape change; magnetically responsive (magnetic NP composites), with remote-controlled movement [46,47,48,49,50].

Advanced fabrication strategies are used to make 4D constructs, such as multi-material printing, when different hydrogels are printed in layers or zones to create regions with distinct responses; photo-crosslinking techniques, where light is used to crosslink gels in precise regions for tunable stiffness and degradation; embedded printing, which is printing into a supporting medium (like a sacrificial gel) to allow for complex, unsupported geometries; and shape memory integration, where hydrogels with shape-memory properties return to a programmed shape when stimulated [46,47,48,49,50].

Main biomedical applications for 4D bioprinting of hydrogels include DDS, TE, and wound healing. Smart hydrogels release therapeutics only when triggered by a specific condition. Dynamic scaffolds fold, stretch, or change shape to guide cell growth and mimicking natural organ folding like intestines or blood vessels. Self-adapting dressings conform to wounds, release drugs, and support tissue regeneration [46,47,48,49,50].

Artificial intelligence (AI)-driven design is transforming the advanced fabrication of hydrogels, especially for biomedical applications like DDS (optimize hydrogel–drug interactions for release profiles), TE (customize stiffness, porosity, degradation for cell types), 4D bioprinting (predict folding/swelling patterns under biological conditions), and wound healing (design hydrogels that adapt to infection, pH, or healing stage). AI is revolutionizing hydrogel development by reducing experimentation, increasing precision, and opening new design spaces that were too complex for traditional methods. AI-driven design of hydrogels means using machine learning (ML) and AI algorithms to design new hydrogel formulations, optimize fabrication parameters (e.g., print speed, crosslinking conditions), predict hydrogel properties before physical synthesis, and automate the discovery of smart, responsive hydrogels. Instead of trial-and-error in the lab, AI models can simulate tens of thousands of compositions or conditions in minutes and suggest the best candidates [51,52,53,54].

AI applications in hydrogel fabrication include materials discovery and design, rheology and printability prediction, 4D and smart behavior modeling, image-based feedback loops, next-generation medical devices, and robotic and automated hydrogel fabrication. AI predicts how a new combination of polymers will behave (e.g., gelation temperature, swelling, degradation, mechanical strength) using deep learning (DL), genetic algorithms, or Bayesian optimization to explore massive chemical spaces. AI can be trained to model the printability and extrudability of hydrogels based on their viscosity, shear-thinning behavior, and temperature sensitivity, helping to tune hydrogel ink to balance cell viability and printing fidelity. AI models can simulate time-dependent transformations of hydrogels under external stimuli (e.g., pH, temperature, light), predicting how a 4D printed hydrogel will fold, swell, or morph post-fabrication. AI can analyze microscopy images or magnetic resonance imaging (MRI) structures of hydrogel to detect defects or inconsistencies, assess cell distribution and viability in bioprinted constructs, and refine printing and crosslinking protocols in real-time. AI-controlled robotic systems can mix and test multiple hydrogel formulations automatically, use reinforcement learning to improve fabrication efficiency over time, and create adaptive feedback systems that auto-correct in real-time during printing [51,52,53,54].

Computer-guided hydrogel design (AI-driven formulation) predict and optimize formulations in silico before lab synthesis. Main applications refer to AI-designed personalized drug carriers and AI-guided bioink customization for patient-specific tissue scaffolds: e.g., a generative model suggests optimal PEG–alginate crosslinking density for controlled insulin release. Challenges and limitations of AI-driven design of hydrogels refer to data scarcity, transferability, interpretability, and lab-to-clinic gaps. Many material systems lack large, high-quality datasets, and AI models trained on one hydrogel system may not generalize well to others. DL can be a “black box”, making it hard to understand why a model recommends a particular formulation. Therefore, translating AI-optimized hydrogels to clinical products still faces regulatory and safety hurdles. Future perspectives of AI-driven design include closed-loop labs, organ-on-a-chip, intelligent implants, neurotechnology, and precision medicine [51,52,53,54].

Synthetic biology–hydrogels combination includes gene circuit-integrated materials such as clustered regularly interspaced short palindromic repeats (CRISPR) used in conjunction with CRISPR-associated (Cas) proteins, particularly Cas9, synthetic promoters, and logic gates embedded into cell-laden hydrogels. Engineered cells inside hydrogel sense a signal, compute logic, and release output (protein, drug) for smart cancer therapies (tumor-sensing gels that release chemo-locally) or biosensors for inflammation, infection, or toxins. CRISPR adds capabilities like gene circuit integration for biosensing or therapeutic response, cell programming inside 3D-printed constructs, and live tissue fabrication with controllable behavior. A CRISPR-enabled bacteria–hydrogel system releases cytokines when it detects tumor microenvironment markers. Also, CRISPR-edited bacteria embedded in hydrogels secretes insulin in response to glucose. CRISPR-engineered bioinks are used for encapsulation of gene-edited cells into hydrogels that sense and respond to environmental cues (e.g., pH, glucose, toxins), release proteins or drugs in a controlled way, and even self-destruct or self-renew under genetic control [55].

Biohybrid systems based on hydrogel–electronic integration (soft electronics or iontronics) refers to conductive or semiconductive hydrogels embedded with graphene, carbon nanotubes (CNTs), poly(3,4-ethylenedioxythiophene) (PEDOT)–polystyrene sulfonate (PSS) or MXenes. It enables signal transmission, actuation, and real-time data readout for smart bandages that assess infection, real-time wound healing monitors, electroceuticals, neuroprosthetics, or brain–machine interfaces. A hydrogel-based wearable patch can measure sweat glucose and deliver insulin using a feedback loop. Hydrogel-based neural interfaces and electroactive systems refer to integration of soft, conductive hydrogels into neural circuits, brain–machine interfaces, and spinal cord regeneration, mimicking the modulus and ion conductivity of neural tissue: e.g., PEDOT–PSS/alginate hydrogels interfacing with cortical neurons to record and stimulate brain activity [56].

Sustainability-focused hydrogel design starts from the observation that many synthetic hydrogels (like poly(vinyl alcohol) (PVA), PEG) are non-degradable and petroleum-derived. Also, crosslinkers can be toxic or persistent in the environment. Green design goals refer to the use of biopolymers (e.g., alginate, chitosan, cellulose, silk), enzymatic or UV-free crosslinking, biodegradable, non-toxic, and renewable components, end-of-life strategies such as compostable, recyclable, or microbial degradation: e.g., hydrogels derived from bacterial cellulose and soy protein (both biodegradable and plant-sourced) used in wound healing [57].

### 1.3. Applications in Clinical Practice

Hydrogels are already being used in clinical practice, and their presence is growing rapidly in DDS, TE, ophthalmology, or wound care. With the ongoing development of AI-driven design, stimuli-responsive hydrogels, and bioprinted tissue constructs, their clinical role will expand in the next 5–10 years [20,58,59,60].

Hydrogels (poloxamers, hyaluronic acid (HA), PEG) enable sustained and localized drug release for chronic diseases, such as rectal or vaginal suppositories using poloxamer thermoresponsive hydrogels (TRHs), hydrogel-based eye drops (e.g., Tears Naturale^®^, Alcon, Geneva, Switzerland) for prolonged lubrication and drug retention, or injectable hydrogel depots for subcutaneous delivery (experimentally used for cancer or arthritis medications). Moreover, hydrogels are used in contact lenses, ocular drug delivery, and corneal implants: e.g., soft contact lenses made from polymers like poly(2-hydroxyethyl methacrylate) (pHEMA), PVA or silicone hydrogels for extended wear; hydrogel-based plugs (e.g., SmartPlug^®^, Seattle, WA, USA) treating dry eye syndrome by blocking tear drainage; hydrogel-based intraocular lenses for cataract surgery [20,58,59,60].

In neurology, hydrogels are being investigated for neural stem cell delivery, stroke recovery, spinal cord injury repair: e.g., PEG-based injectable hydrogel for supporting regeneration in spinal cord injury patients, already considered for clinical trial. Hydrogels for TE and RM (ECM mimetic hydrogels) are increasingly used in cell encapsulation, 3D cell culture, and even bioprinted implants. Dermagraft^®^ (Organogenesis Inc., Canton, MA, USA), a bioengineered human dermis embedded in a hydrogel scaffold, is used for the treatment of diabetic foot ulcers. Matrigel^®^ (Corning Life Sciences, Tewksbury, MA, USA) is a common hydrogel used in preclinical research for tumor models and stem cell culture, though not yet for direct clinical implantation. Hydrogels serve as scaffolds or injectable materials (PVA, gelatin, collagen) for cartilage regeneration and bone healing. GelrinC^®^ (Regentis Biomaterials, Or Akiva, Israel), a biodegradable hydrogel for treating knee cartilage defects, is clinically used in Europe. Also, Cartiva^®^ (Cartiva, Inc., Alpharetta, GA, USA), a synthetic cartilage implant, is a PVA-based hydrogel implant used in great toe arthritis [20,58,59,60].

Hydrogels based on Carbopol, PEG, or alginate blends are widely used in chronic wound management, including diabetic ulcers, burns, and surgical wounds, maintaining moist environment, absorbing exudate, promoting healing, delivering antibiotics or growth factors (GFs): e.g., Intrasite Gel^®^ (Smith & Nephew, London, UK), a sterile amorphous hydrogel for moist wound healing; Purilon Gel^®^ (Coloplast, Humlebaek, Denmark), a hydrogel for necrotic and sloughy wounds; and Aquacel^®^ Hydrofiber (ConvaTec, Reading, UK), a hydrogel-infused dressing with silver (Ag) for antimicrobial action. Hydrogels made from crosslinked HA are widely used in dermal fillers for cosmetic procedures. Juvederm^®^ (Allergan Aesthetics, Dublin, Ireland), Restylane^®^ (Galderma, Lausanne, Switzerland), and Belotero^®^ (Merz Aesthetics, Frankfurt, Germany) are HA-based dermal fillers applied for wrinkle treatment and facial volume enhancement [20,58,59,60].

Several hydrogel delivery systems are already in clinical trials, including those for non-muscle invasive bladder cancer, basal cell carcinoma, and hypervascular cancer. ReGel^®^ (BTG/Protherics, London, UK) is a formulation based on thermoresponsive triblock copolymer (Poloxamer 407/188). Phase II clinical trials with Paclitaxel–ReGel^®^ demonstrate effectiveness at reducing systemic toxicity and local drug levels significantly higher than traditional methods. Injectable hydrogel solidifies at body temperature, ensuring a sustained release of chemotherapy at tumor site (e.g., for pancreatic cancer). Main barriers include manufacturing scale-ups of sterile, injectable formulations and narrow application windows (e.g., locally advanced, non-resectable tumors). A thermosensitive PEG-*co*-poly(glycolic acid) hydrogel formulation (Encapson, Enschede, The Netherlands) is used as long-acting local anesthetic for postoperative pain. It is an injectable liquid at room temperature, forming gel in situ and slowly releasing bupivacaine for localized pain relief. Phase II clinical trials showed an extended analgesia for up to three days, with patient pain scores improved vs. conventional bupivacaine. Precise control of drug release is technically challenging in terms of gelation temperature and release kinetics that need to be optimized. Also, regulatory concerns over polymer biodegradation byproducts are an important barrier [44,58,61,62].

Vascugel^®^ (Tengion/Baxter, Winston-Salem, NC, USA), an ECM-mimetic hydrogel with endothelial cells, is recommended for vascular access patency enhancement in dialysis patients. Injected around arteriovenous grafts during placement, it promotes endothelialization and reduces intimal hyperplasia. Completed Phase II clinical trials highlighted that hydrogel provided effective cell delivery and retention, with an improved blood flow and graft patency. Barriers include difficulty in standardizing the cell component, and manufacturing and cost scaling issues. It was eventually shelved despite promising results due to business/market challenges. GelrinC^®^ (Regentis Biomaterials, Or Akiva, Israel) includes a PEGylated fibrinogen-based hydrogel formulation for the treatment of cartilage defects in the knee. Completed Phase II in the European Union (EU) and ongoing Phase III clinical trials in the U.S. (Food and Drug Administration (FDA) Investigational Device Exemption granted) exhibited that injectable, ultraviolet (UV)-crosslinked hydrogel scaffold degrades over months and promotes regeneration of native cartilage. Lessons learned refer to scaffold stiffness and degradation that match native cartilage healing timelines, and easy delivery via arthroscopy is a key commercial advantage. High cost of production and complex regulatory path for combination (biomaterial and device) products are the main barriers. HyStem^®^ (Glycosan/BioTime, Carlsbad, CA, USA), included in Phase II/III clinical trials (U.S.), is a HA-based hydrogel designed for stem cell delivery for ischemic limb disease and vocal cord repair. Injectable, in situ crosslinking matrix supports cell survival and integration in damaged tissue. Cell–hydrogel interaction, critical to efficacy and crosslinking chemistry balancing structure, and cell viability can be considered the most important lessons learned. Several barriers such as expensive and complex logistics for cell-therapy combo, together with storage and shelf-life issues of biologically active gels, must be considered [44,58,61,62].

This review aims to provide a comprehensive and forward-looking analysis of recent innovations in hydrogel science, emphasizing next-generation biomedical applications such as microscale-engineered drug carriers, 3D/4D bioprinted tissue scaffolds, and multifunctional wound healing platforms. A particular focus is placed on emerging biohybrid and composite hydrogel systems, which incorporate NPs, peptides, or bioactive macromolecules to expand functional versatility. In addition to discussing material classifications and structure–function relationships, this work critically evaluates the challenges and regulatory constraints that must be addressed to realize the full translational potential of hydrogel technologies. By bridging fundamental polymer science with applied BME, this review endeavors to inspire future innovation and contribute to the development of clinically impactful hydrogel-based solutions.

## 2. Natural Hydrogels for Biomedical Applications

### 2.1. Overview

Natural hydrogels, derived from biopolymers of plant, animal, and microbial origin, have emerged as versatile and indispensable materials in modern biomedical science. Each category brings distinct physicochemical characteristics that define their functionality across therapeutic platforms [6,7,8,9,10,17,18,19,20]. Plant-based polysaccharides, such as alginate and pectin, are renowned for their excellent gel-forming capacity and high biocompatibility [6,7,8,9,16,17,20,63,64,65,66,67]. Animal-derived proteins, including gelatin, collagen, and fibrin, offer superior mechanical strength and intrinsic bioactivity [6,7,8,9,16,17,20,63]. Meanwhile, microbial polysaccharides, such as gellan gum and bacterial cellulose, are increasingly investigated due to their reproducibility, structural versatility, and tunable biofunctional properties [6,7,8,9,20,66,68].

Natural hydrogels, as hydrophilic 3D polymeric networks, exhibit key attributes such as biocompatibility, biodegradability, and structural similarity to the native ECM. These features render them ideal candidates for scaffold fabrication, tissue repair (TR), and DDS [16,20,63,64,65,66,67,68].

The medical use of natural hydrogels dates back centuries, with early applications involving plant gums and animal gelatin for wound healing. However, the scientific inquiry into their potential accelerated in the mid-20th century. Between the 1950s and 1970s, materials such as agarose and gelatin became staples in microbiological and tissue culture applications, while alginate emerged as clinical wound dressing due to its ion-responsive gelation and moisture-retention properties [6,7,8,9,19,20,63,64,65,66,67]. In subsequent decades, the focus expanded to ECM-mimetic polymers like HA, chitosan, and collagen, which demonstrated exceptional biointegration and cell-supportive behavior [6,7,8,9,17,63,64,65,66,67,69,70]. From the 2000s onward, advances in nanotechnology, bioengineering, and 3D bioprinting have led to the development of composite hydrogels, integrating natural and synthetic components to overcome the mechanical and functional limitations of earlier generations. Today, natural hydrogels are foundational in RM, being applied in TE-based scaffolds, injectable cell delivery matrices, wound care, and targeted therapeutic platforms [6,7,8,9,31,32,33,34,63,64,65,66,67,70].

Natural hydrogels are generally classified by their crosslinking mechanism into: (*i*) Physically crosslinked hydrogels: These are formed via non-covalent interactions, including hydrogen bonding, ionic interactions, and hydrophobic associations. Examples include gelatin and alginate. They are typically reversible and responsive to environmental stimuli, which is advantageous for dynamic systems, but they often exhibit limited mechanical robustness and rapid degradation; (*ii*) Chemically crosslinked hydrogels: These involve covalent bond formation, leading to enhanced structural stability and resistance to enzymatic breakdown. Chitosan and pectin-based hydrogels fall into this category. Their durability and controlled degradation make them well-suited for sustained drug delivery and wound management [6,7,8,9,20,63,64,65,66].

Natural hydrogels exhibit several key characteristics that make them suitable for biomedical applications: (*i*) High water content (often exceeding 90%) facilitates efficient diffusion of nutrients, oxygen, and bioactive molecules, thereby supporting cellular viability, promoting tissue regeneration, and enabling biochemical signaling. Their hydrophilic nature also closely mimics the native ECM, making them ideal scaffolding materials for TE. In addition to their hydration capabilities, natural hydrogels inherently exhibit bioactivity by presenting native ligands that support cellular adhesion, proliferation, and differentiation; (*ii*) Intrinsic bioactivity, supporting cell adhesion, proliferation, and differentiation through natural ligand presentation; (*iii*) Tunable porosity, mechanical strength, and degradation rates via chemical or physical modifications, allowing for application-specific optimization; (*iv*) Cytocompatibility and low immunogenicity, minimizing adverse inflammatory responses and making them especially attractive for in vivo applications in RM and the development of biomedical devices; (*v*) Biodegradability, ensuring safe metabolic clearance without generating harmful byproducts, further enhancing their clinical appeal. In wound management, natural hydrogels function as moisture-retentive dressings that maintain a hydrated microenvironment conducive to healing by supporting cellular migration, preventing scab formation, and protecting against microbial invasion. Their barrier properties and inherent bioactivity help reduce infection risks while accelerating TR; (*vi*) Considerable functional versatility, as they can be engineered to incorporate bioactive molecules, such as GFs, peptides, and NPs, to modulate specific cellular behaviors or serve as vehicles for controlled drug delivery. This adaptability extends their utility beyond biomedicine into areas like pharmaceuticals, food technology, and environmental science. These features collectively enable natural hydrogels to function as ECM analogs, promoting tissue regeneration, supporting biological signaling, and integrating seamlessly with surrounding tissues [6,7,8,9,20,63,64,65,66,67,68,70].

Despite their promise, natural hydrogels face limitations such as low mechanical strength, rapid degradation, source variability, high production costs, and potential immunogenicity. Current research focuses on mitigating these issues through strategies like hybridization with synthetic polymers, nanomaterials, and advanced crosslinking methods (e.g., photo-crosslinking, enzymatic processes). Innovations such as “smart” hydrogels that respond to pH, temperature, or light; biofunctionalization with peptides or GFs; and use in 3D bioprinting are rapidly expanding their biomedical utility [6,7,8,9,20,63,64,65,66,67,68,70].

Sustainability is also becoming a key trend, with increasing interest in marine-derived or agrowaste-based biopolymers. Multidisciplinary efforts in material science, biotechnology, and clinical engineering are expected to drive the clinical translation of natural hydrogels, enhancing their performance in RM, precision drug delivery, and implantable devices [6,7,8,9,20,63,64,65,66,67,68,70].

Natural hydrogels can be further categorized based on the type of biopolymer from which they are derived. Each class contributes distinct physicochemical and biological properties, making them suitable for specific biomedical applications [6,7,8,9,20,63,64,65,66,67,68,70]. These ongoing advancements are positioning natural hydrogels as cornerstone materials for next-generation biomedical solutions.

Table 1 includes some quantitative data, such as Young’s modulus, tensile strength, swelling ratio, gelation time at 37 °C, in vitro degradation time, and porosity, to compare natural hydrogels for biomedical applications. Table 2 summarizes advantages, limitations, and key applications for an overall reference on natural hydrogels [9,63,71,72].

### 2.2. Protein-Based Hydrogels

Protein-based hydrogels represent a critical class of biomaterials in the biomedical and pharmaceutical fields due to their superior biocompatibility, inherent bioactivity, and tunable mechanical properties [6,7,8,9,16,17,20,63,64,65,66,67]. These materials are particularly advantageous for replicating the ECM, offering native cell-binding motifs such as the arginine–glycine–aspartic acid (RGD) sequence, which facilitate cell adhesion, migration, and differentiation. As a result, protein hydrogels have found widespread applications in TE, DDS, and wound healing platforms. Notable examples include gelatin (a denatured form of collagen), collagen itself (the most abundant ECM protein in animals), fibrin (derived from fibrinogen in blood plasma), silk fibroin (SF; extracted from silkworm cocoons), and casein (a major milk protein) [6,7,8,9,16,17,20,63]. These proteins support favorable biological interactions while enabling enzymatically controlled biodegradability. Despite their promise, protein-based hydrogels face critical limitations that can hinder their clinical translation. Thermosensitivity remains a major concern, particularly with gelatin, which undergoes gel-to-sol transition near physiological temperatures unless chemically stabilized [6,7,8,9,16,17,20,63]. Furthermore, their animal-derived origin poses potential immunogenicity risks, and their degradation rates may be too rapid to support long-term tissue regeneration or sustained drug release. Achieving consistent structural stability and precise control over crosslinking mechanisms, whether physical, enzymatic, or photo-induced, remains technically challenging. Moreover, the availability and reproducibility of tissue-specific proteins can limit scalability and standardization in biomedical applications [6,7,8,9,16,17,20,63].

To overcome these barriers, current research is focusing on the development of next-generation protein-based hydrogels with enhanced functionality. Innovations include genetically engineered proteins with programmable sequences and mechanical properties, as well as the incorporation of photo-crosslinkable moieties that allow for spatiotemporal control during in situ applications. These advancements are facilitating the design of injectable protein hydrogels for minimally invasive therapies and “smart” wound dressings with dynamic responsiveness. Moving forward, synergistic integration of synthetic biology, nanotechnology, and biofabrication is expected to yield highly customizable protein hydrogels tailored for RM, controlled therapeutic delivery, and precision tissue modeling [6,7,8,9,16,17,20,63].

#### 2.2.1. Animal-Derived Proteins

Animal welfare, environmental impact, transparency and labeling, and religious and cultural issues are considered as ethical concerns in using animal-derived proteins (e.g., collagen or silk). Collagen often comes from cow or pig skin/bones, and silk from silkworm cocoons, usually involving killing the animals and insects, respectively. Practices vary, but in many industrial settings, animals are raised in intensive farming systems with questionable welfare standards. Silkworms are boiled alive to preserve long silk fibers, a major concern for insect welfare advocates. Animal agriculture contributes significantly to greenhouse gas emissions, deforestation, water usage, and waste. Silk farming, while smaller in scale, still has ecological footprints, including the use of mulberry trees and energy-intensive processes. Many products with animal-derived proteins do not clearly state their origins and ethical consumers may unknowingly use animal-based substances. Products derived from pigs or cows may conflict with religious dietary laws (e.g., Islam, Judaism, Hinduism). Moreover, some consumers object to animal use entirely due to vegan or spiritual beliefs [73,74].

Ethical and sustainable alternatives of animal-derived proteins are represented by recombinant (lab-grown) and plant-based proteins. Recombinant or biotech-based proteins are made by inserting animal genes into bacteria or yeast, which then produce the protein in fermentation tanks, e.g., lab-grown collagen, or bioengineered spider silk. Ethical advantages refer to no animals killed, highly controlled production with less environmental impact, and customizable properties (strength, texture). Caveats include the use of animal genes in production, sometimes involving genetically modified organisms (GMOs), which some consumers dislike (though risks are minimal). Plant-based proteins derived from sources like soy, pea, or hemp, or engineered from plants are used to mimic animal proteins. Fully vegan, no animal exploitation, lower environmental footprint, and suitability for religious and cultural diets are the main ethical advantages. Several challenges should be considered for plant-derived proteins, such as they may not always perfectly mimic animal protein properties, allergen concerns (e.g., soy), and they are less suitable for some specialized uses (e.g., biomedical-grade collagen) [73,74].

##### Collagen- and Gelatin-Based Hydrogels

Structural Properties

Collagen is the most abundant structural protein in mammals and a principal component of connective tissues. Among its isoforms, type I collagen is most employed in hydrogel formulations due to its prevalence in the ECM of skin, tendon, and bone. Type II and type III collagen are suited for cartilage and vascular tissues, respectively, while type IV collagen, a key component of basement membranes, is essential for applications requiring specialized microenvironments [6,7,8,9,16,17,20,63,75,76,77]. Collagen’s triple-helix structure, stabilized by glycine, proline, and hydroxyproline, provides high mechanical strength and thermal stability, with the repetitive Gly–Pro–X sequence critical to maintaining this conformation [75,76].

Upon denaturation through heat or chemical treatment, collagen yields gelatin, a denatured biopolymer that retains essential bioactivities while adopting a more flexible, linear structure [6,7,8,9,16,17,20,63,75,76,77]. Gelatin exhibits thermoresponsive sol–gel transitions, enabling its use in injectable hydrogels and controlled drug delivery. Its lower gelation temperature enhances processing ease and adaptability across physiological conditions [6,7,8,9,16,17,20,63,78]. Rich in glycine and proline, gelatin supports cell adhesion, proliferation, and differentiation. Its multifunctionality has led to extensive use in TE, where it provides biocompatible scaffolds, and in DDS, where it enables precise therapeutic release [6,7,8,9,16,17,20,63,78,79,80].

Fabrication Methods

Collagen- and gelatin-based hydrogels can be fabricated using physical, chemical, or self-assembly methods. Thermal modulation enables reversible sol–gel transitions for gelatin- and collagen-based systems, advantageous in injectable formats. Lyophilization produces porous architectures that improve cell infiltration and nutrient flow. Chemical crosslinking enhances stability and longevity, while photopolymerization allows spatial control via UV or visible (VIS) light for minimally invasive applications. Self-assembly under physiological conditions forms ECM-mimetic fibrillar networks without external crosslinkers. These approaches enable fine-tuning of biochemical and mechanical properties, supporting a wide array of TE, RM, and therapeutic delivery applications [6,7,8,9,16,17,20,63,78,79,80].

Hydrogel integrity is maintained via physical or chemical crosslinking [6,7,8,9,16,17,20,63,81]. Chemical agents like glutaraldehyde (GAD) and genipin (GNP) form stable covalent networks, improving mechanical strength and enzymatic resistance, while physical interactions (e.g., ionic bonding, hydrogen bonding, temperature-induced gelation) allow for reversible and stimuli-responsive systems [81,82,83,84]. Properties like tensile strength can be tailored by adjusting polymer concentration, degree of crosslinking (DC), or hybrid polymer incorporation. Both collagen and gelatin possess intrinsic biocompatibility and bioactivity, with RGD sequences promoting integrin-mediated cell binding. Their enzymatic degradability by collagenases and gelatinases facilitates gradual matrix replacement during tissue healing. Additionally, their high swelling capacity in aqueous environments supports drug encapsulation and controlled release [6,7,8,9,16,17,20,63,78,79,80].

Biomedical Applications

Collagen- and gelatin-based hydrogels serve as multifunctional biomaterials with broad applicability in biomedicine, owing to their excellent biocompatibility, biodegradability, and ability to mimic the native ECM [6,7,8,9,16,17,20,42,44,63,78,79,80]. In TE, these hydrogels are widely employed for soft tissue regeneration (e.g., skin, cartilage) and bone regeneration, where collagen scaffolds are often mineralized with hydroxyapatite to enhance osteoconductivity, while gelatin is incorporated to improve mechanical integrity and cellular interactions [7,9,10,42,43,44,45,77,81]. In drug delivery, both materials act as effective matrices for controlled release systems, capable of encapsulating and gradually releasing therapeutic agents such as small-molecule drugs, proteins, or nucleic acids [6,7,8,9,16,17,20,42,44,63,78,79,80]. Gelatin’s lower gelation temperature enables stimuli-responsive behavior, particularly advantageous in thermally triggered release systems. Their inherent moisture retention, biodegradability, and promotion of cell migration make collagen and gelatin hydrogels ideal for wound healing applications, where they support TR while reducing infection risk [6,7,8,9,16,17,20,42,44,63,78,79,80]. Furthermore, they provide highly tunable 3D environments for cell culture, facilitating in vitro models that more closely replicate physiological conditions, useful for studying cell behavior, tissue development, and pharmacological testing. Recent innovations have expanded their potential through the development of hybrid hydrogels, where blends with natural or synthetic polymers (e.g., alginate, chitosan, PEG) improve mechanical strength, degradation control, and functional versatility [6,7,8,9,16,17,20,42,44,63,78,79,80]. The emergence of “smart” hydrogels, responsive to environmental stimuli such as pH, temperature, or light, offers precise spatiotemporal control over drug release and scaffold behavior. Moreover, collagen and gelatin-based bioinks have become essential in 3D bioprinting, enabling the fabrication of complex tissue constructs with accurate architectural and cellular organization for RM. The integration of nanotechnology, via incorporation of NPs such as gold (Au), Ag, or graphene, further augments these hydrogels by enhancing antibacterial activity, electrical conductivity, and targeted delivery capabilities. As research progresses, the combination of biomolecular engineering, advanced manufacturing, and nanotechnological strategies continues to elevate the performance of collagen- and gelatin-based hydrogels, solidifying their role in next-generation biomedical devices, therapeutic systems, and regenerative therapies [6,7,8,9,16,17,20,42,44,63,78,79,80].

##### Elastin-Based Hydrogels

Structural Properties

Elastin is a fundamental fibrous protein of the ECM, predominantly localized in dynamic connective tissues such as skin, lungs, elastic cartilage, and blood vessels, where it imparts essential biomechanical properties such as elasticity, compliance, and resilience under repetitive mechanical strain [6,7,8,9,16,17,20,42,44,63,85,86,87,88]. These functional attributes stem from elastin’s unique amino acid composition, which is rich in glycine, proline, valine, and hydrophobic residues (e.g., alanine, leucine, isoleucine) that facilitate self-assembly through coacervation, while lysine-derived crosslinks, particularly desmosine and isodesmosine, enable the formation of a covalently bonded, insoluble, and mechanically stable network capable of withstanding cyclical deformation [85,86,87,88]. Elastin-based hydrogels are engineered to mimic these native mechanical and structural characteristics, providing elastic scaffolds that support physiological tissue loads and promote cellular function [6,7,8,9,16,17,20,42,44,63,85,86,87,88].

Fabrication Methods

Fabrication approaches include chemical crosslinking, employing agents such as 1-ethyl-3-(3-dimethylaminopropyl) carbodiimide (EDC), *N*-hydroxysuccinimide (NHS), or GNP to establish covalent bonds between functional groups within elastin or elastin-like polypeptide (ELP) chains, producing robust hydrogels ideal for load-bearing applications like dermal matrices and vascular grafts [6,7,8,9,16,17,20,42,44,63,85,86,87,88]. Physical crosslinking strategies leverage thermally induced coacervation and pH-responsive interactions to form reversible hydrogels with injectable and stimuli-responsive properties, facilitating minimally invasive application and in situ gelation. Functionally, elastin-based hydrogels exhibit high mechanical resilience, extensibility, and rapid elastic recovery, properties essential for replicating the biomechanics of cardiovascular, pulmonary, and dermal tissues. ELPs, often designed with pentapeptide motifs such as VPGVG, allow for precise modulation of mechanical properties, degradation kinetics, and stimuli responsiveness [6,7,8,9,16,17,20,42,44,63,85,86,87,88].

Biomedical Applications

These hydrogels are inherently biocompatible and biodegradable, promoting cellular adhesion, proliferation, and ECM deposition. Functionalization with bioactive peptides, such as RGD motifs, enhances integrin-mediated signaling, supports rapid wound closure, and facilitates tissue remodeling. Their gradual in vivo degradation aligns with the timeline of tissue regeneration, making them suitable for regenerative scaffolding without requiring surgical removal [6,7,8,9,16,17,20,42,44,63,85,86,87,88,89,90]. However, maintaining structural and mechanical stability under physiological stress remains a significant limitation, especially in dynamic environments like vascular or myocardial tissues [6,7,8,9,16,17,20,42,69,89,90]. To overcome this, emerging crosslinking strategies, including dynamic covalent bonds (e.g., Schiff base formation, Michael-type addition), photo-crosslinkable derivatives (e.g., methacrylated elastin), and click chemistry, are being developed to enhance mechanical durability and enable controlled degradation and stimuli-triggered gelation [82,84,85,86,87,88,89]. In addition, hybrid hydrogel systems incorporating elastin with other biopolymers (e.g., gelatin, chitosan, HA, SF) demonstrate synergistic improvements in mechanical strength, moisture retention, and biological functionality. For instance, elastin–gelatin composites have exhibited enhanced cardiomyocyte alignment, contractility, and electrical coupling, supporting their use in cardiac scaffolds [6,7,8,9,16,17,20,42,44,63,85,86,87,88,89,90].

Elastin-based hydrogels are proving increasingly effective in several biomedical applications. In skin wound healing, they maintain a moist environment, support keratinocyte and fibroblast migration, and enhance ECM remodeling [6,7,8,9,16,17,20,42,44,63,85,86,87,88,89,90,91]. Notably, elastin–HA composite hydrogels have been shown to significantly improve dermal regeneration in diabetic wound models [92]. In vascular TE, their mechanical compliance and hemocompatibility make them excellent candidates for small-diameter vascular grafts, where they mimic the biomechanical behavior of native arteries and reduce thrombotic risk. In cardiac TE, elastin-enriched hydrogels promote cardiomyocyte viability, alignment, and synchronized contraction, with in vitro and in vivo studies reporting enhanced functional integration following myocardial infarction [6,7,8,9,16,17,20,42,44,63,85,86,87,88,89,90,91].

Elastin-based hydrogels offer a bioinspired, adaptable platform that effectively integrates mechanical elasticity with biological functionality. Ongoing advances in molecular design, hybridization, and fabrication techniques continue to improve their performance, establishing them as promising materials for next-generation regenerative therapies, including wound dressings, vascular grafts, and engineered cardiac tissues [6,7,8,9,16,17,20,42,44,63,85,86,87,88,89,90,91,92].

##### Fibrin-Based Hydrogels

Structural Properties

Fibrin-based hydrogels are derived from fibrin, a natural, insoluble fibrous protein that plays a fundamental role in hemostasis and TR. Fibrin originates from fibrinogen, a soluble plasma glycoprotein produced by the liver. Upon vascular injury, thrombin, a serine protease activated in the coagulation cascade, cleaves fibrinogen, releasing fibrinopeptides A and B. This cleavage triggers the polymerization of fibrin monomers into a crosslinked, 3D fibrous network, forming a provisional ECM. This scaffold stabilizes the clot and facilitates cellular adhesion, migration, and proliferation, critical for effective wound healing and tissue regeneration. The structure and biological functionality of fibrin are further determined by its amino acid composition, which includes a balanced mix of glycine, alanine, proline, glutamic acid, lysine, and serine, each contributing to mechanical flexibility, electrostatic interactions, enzymatic activity, and cellular communication [6,7,8,9,16,17,20,42,44,63,93,94,95,96,97,98,99,100,101,102].

Fabrication Methods

Fibrin hydrogels are typically synthesized by mixing fibrinogen with thrombin, initiating rapid gelation into a hydrated matrix. They possess several properties that make them highly suitable for biomedical applications. These include excellent biocompatibility, ECM-mimicking architecture, and a porous structure that supports nutrient diffusion and cell infiltration. Additionally, fibrin hydrogels can encapsulate and gradually release GFs such as vascular endothelial growth factor (VEGF), promoting angiogenesis and sustained tissue regeneration. Their mechanical properties are tunable by altering fibrinogen or thrombin concentrations or through additional crosslinking strategies, making them adaptable to both soft and relatively more rigid tissue environments. Natural biodegradation via proteolytic enzymes like plasmin ensures the scaffold is progressively replaced by native tissue, which is especially beneficial in applications such as myocardial repair, where gradual integration into cardiac tissue is crucial [6,7,8,9,16,17,20,42,44,63,93,94,95,96,97,98,99,100,101,102].

Biomedical Applications

The biomedical potential of fibrin-based hydrogels spans diverse areas. They have shown efficacy in managing chronic wounds by maintaining a moist healing environment and promoting angiogenesis and granulation tissue formation. In surgical settings, they serve as adhesives for hemostasis and tissue sealing. Furthermore, they are employed as scaffolds in TE for skin, cartilage, and vascular grafts due to their ability to support cellular attachment, proliferation, and differentiation. Their ease of preparation using readily available components (fibrinogen and thrombin) offers a practical advantage in clinical environments, where rapid intraoperative deployment is often essential [6,7,8,9,16,17,20,42,44,63,93,94,95,96,97,98,99,100,101,102].

Challenges and Future Perspectives

Certain limitations hinder the broader clinical use of fibrin hydrogels. These include limited mechanical strength, particularly in load-bearing tissues, and rapid degradation, which may not provide sufficient structural support during prolonged healing. Additionally, fibrin sourced from human plasma poses risks of pathogen transmission, and variability in fibrinogen quality can lead to inconsistent hydrogel performance. Inflammatory responses and complex cell–matrix interactions can also affect regenerative outcomes. Lastly, challenges related to storage stability and short shelf-life complicate logistics in clinical applications. To address these issues, ongoing research focuses on developing modified or hybrid fibrin materials, optimizing crosslinking methods, and integrating advanced drug and cell delivery strategies to enhance the functionality and translational potential of fibrin-based hydrogels in RM [6,7,8,9,16,17,20,42,44,63,93,94,95,96,97,98,99,100,101,102].

##### Albumin-Based Hydrogels

Structural Properties

Albumin exhibits remarkable solubility, thermal and structural stability, and a high capacity for binding endogenous and exogenous compounds. Its amino acid profile comprises both essential amino acids (e.g., leucine, lysine, methionine, phenylalanine, threonine, tryptophan, valine) and non-essential amino acids (e.g., alanine, arginine, aspartic acid, cysteine, glutamic acid, glycine, proline, serine, tyrosine) serum. Approximately 40% of these residues are hydrophobic and 60% are hydrophilic, conferring amphiphilic properties that enable albumin to encapsulate hydrophobic drugs and maintain colloidal osmotic pressure. Particularly, the high content of leucine (~9%) and glutamic acid (~10%) enhances the protein’s interaction with both small therapeutic molecules and biological environments. Human serum albumin (HSA) is the most commonly employed in biomedical applications due to its superior biocompatibility, low immunogenicity, and physiological relevance serum [6,7,8,9,16,17,20,42,44,63,103,104,105].

Fabrication Methods

Albumin-based hydrogels are formulated using albumin, a water-soluble globular protein primarily derived from human or animal serum. Albumin-based hydrogels offer a suite of advantages for biomedical use serum. Their tunable viscoelastic and biochemical properties can be modulated by varying the albumin concentration and the DC, allowing for customization based on specific therapeutic needs serum [6,7,8,9,16,17,20,42,44,63,103,104,105].

Biomedical Applications

In terms of biomedical applications, albumin-based hydrogels have shown promise in both DDS and TE. In oncology, albumin hydrogels have been employed as carriers for chemotherapeutic agents such as paclitaxel, enabling sustained and localized drug release, thereby reducing systemic toxicity and enhancing therapeutic impact. In RM, these hydrogels have been explored for cardiac TE, where they mimic native ECM conditions and support cell viability, proliferation, and function. Their injectable nature and biodegradability further expand their utility in minimally invasive procedures and in situ tissue regeneration strategies [6,7,8,9,16,17,20,42,44,63,103,104,105].

Challenges and Future Perspectives

Albumin-based hydrogels exhibit excellent biocompatibility and minimal immune response, especially when prepared from HSA. Furthermore, they provide a stable matrix for drug encapsulation and controlled release, which enhances therapeutic efficacy and minimizes off-target effects. The mild gelation conditions often employed in albumin hydrogel synthesis help preserve the activity of encapsulated bioactive agents, including small molecules, proteins, and cells. Despite their potential, albumin hydrogels face several limitations. Source-related variability in serum-derived albumin can lead to batch-to-batch inconsistencies in hydrogel properties. Additionally, there is a non-negligible risk of contamination and pathogen transmission when using biologically sourced albumin, necessitating rigorous purification protocols and quality control (QC) measures. The relatively soft mechanical properties of native albumin hydrogels may also limit their applicability in load-bearing TE without additional structural reinforcement [6,7,8,9,16,17,20,42,44,63,103,104,105].

##### Silk Fibroin-Based Hydrogels

Structural Properties

SF derives from the primary structural protein found in the cocoons of the *Bombyx mori* (Linnaeus, 1758), silkworm moth (*Bombycidae*), obtained through the degumming process that removes sericin. SF constitutes approximately 75% of the silk fiber and is highly valued for its outstanding mechanical strength, tunable biodegradability, biocompatibility, and minimal immunogenicity. Its amino acid composition is dominated by glycine (~44%), alanine (~30%), and serine (~12%), which facilitate the formation of β-sheet crystallites, key contributors to the hydrogel’s mechanical robustness and stability. In contrast, the amorphous regions consisting of non-repetitive sequences provide flexibility and hydration capacity. Minor constituents such as tyrosine, valine, aspartic acid, and threonine contribute to inter- and intramolecular interactions, hydrogen bonding, and hydrophilicity. The dual-phase organization of crystalline β-sheets and amorphous regions gives SF hydrogels a unique balance of mechanical integrity and processability, positioning them as ideal candidates for biomedical applications such as drug delivery, wound healing, and tissue regeneration [6,7,8,9,16,17,20,42,44,63,106,107,108,109,110].

Fabrication Methods

Gelation mechanisms of SF are diverse and allow for precise tailoring of material properties. Physical gelation occurs via hydration and incubation (aging), promoting molecular self-assembly and β-sheet formation without chemical additives. Chemical crosslinking, using agents such as GAD or GNP, enhances network stability and mechanical properties. Solvent-induced gelation is achieved by altering parameters like pH, ionic strength, or solvent polarity to trigger conformational changes and phase separation. Thermally induced gelation leverages heat to accelerate β-sheet formation and structural reorganization. These versatile gelation routes allow researchers to fine-tune the hydrogel’s stiffness, porosity, degradation rate, and swelling behavior according to the intended application [6,7,8,9,16,17,20,42,44,63,106,107,108,109,110].

Biomedical Applications

Biomedical and cosmetic applications of SF hydrogels are broad and continually expanding. In wound care, SF hydrogels maintain a moist healing environment and promote faster epithelial closure, especially when combined with bioactive agents such as GFs. In DDS, SF serves as a biocompatible carrier enabling controlled and sustained release [6,7,8,9,16,17,20,42,44,63,106,107,108,109,110]. For example, composite hydrogels incorporating sodium alginate have demonstrated enhanced mechanical strength and extended drug release in vivo [111]. In TE, SF hydrogels support cellular activities and ECM mimicry. Nanofibrous hydrogels produced via electrospinning present high porosity and promote chondrocyte growth, indicating their potential for cartilage regeneration [6,7,8,9,16,17,20,42,44,63,106,107,108,109,110,111]. Blends of SF with HA have been explored for skin and soft tissue repair, showing improved healing and dermal regeneration [112,113]. In the cosmetic industry, SF is used for its hydrating and anti-aging properties [111,114], while in the food sector, silk-based hydrogels emerge as biodegradable alternatives for packaging and encapsulation of nutrients and flavors [115].

Challenges and Future Perspectives

SF hydrogels possess a suite of desirable properties. Their excellent biocompatibility results in minimal inflammatory response upon implantation. They exhibit high tensile strength and elasticity, making them suitable for load-bearing tissue scaffolds. The hydrogels are biodegradable into non-toxic amino acid byproducts, ideal for temporary matrices in TR. Furthermore, their superior water retention capacity creates a hydrated microenvironment that supports cell adhesion, proliferation, and tissue regeneration. These features, coupled with tunable architecture and functionalization potential, make SF hydrogels a highly adaptable platform [6,7,8,9,16,17,20,42,44,63,106,107,108,109,110]. Continued innovation in formulation and processing techniques is expected to further broaden the application spectrum of SF-based hydrogels, particularly in personalized medicine, sustainable packaging, and advanced regenerative therapies.

##### Sericin-Based Hydrogels

Structural Properties

Sericin is a hydrophilic glycoprotein obtained as a byproduct during the degumming of silk cocoons [6,7,8,9,16,17,20,42,44,63,116]. Structurally, sericin is rich in both hydrophilic and hydrophobic amino acids. Hydrophilic amino acids such as serine (~30%), glycine, threonine, asparagine, and glutamic acid contribute to its moisture-retaining capacity and biocompatibility. Meanwhile, hydrophobic amino acids like alanine, valine, and phenylalanine provide additional stability and influence intermolecular interactions, enhancing gel formation [116].

Fabrication Methods

Traditionally considered waste in the silk industry, sericin has garnered increasing attention for biomedical and cosmetic applications due to its unique physicochemical and biological properties, including excellent water solubility, bioadhesiveness, and film-forming ability. These attributes enable the formulation of hydrogels with desirable characteristics for skin regeneration, wound healing, and TE [6,7,8,9,16,17,20,42,44,63,116,117,118,119,120,121,122,123].

Biomedical Applications

The advantages of sericin-based hydrogels lie in their intrinsic biocompatibility, non-toxicity, and ability to support cellular activity. In vitro studies have demonstrated that sericin promotes fibroblast adhesion, proliferation, and migration, critical factors in wound repair and tissue regeneration. Its high serine content facilitates water retention, maintaining a moist wound environment that accelerates healing and reduces scar formation. Furthermore, sericin hydrogels are biodegradable, undergoing hydrolytic degradation into non-toxic byproducts, which supports their integration into biological systems without eliciting significant immune responses [6,7,8,9,16,17,20,42,44,63,117,118,119,120,121,122,123].

Biomedical and cosmetic applications of sericin-based hydrogels are diverse. In wound healing, sericin hydrogels have shown promising results, particularly in chronic wounds such as diabetic ulcers [117,124,125]. In the cosmetic industry, sericin is incorporated into skincare products for its moisturizing and anti-aging properties. Additionally, its biocompatibility and water retention ability make it a candidate for injectable dermal fillers and as a base for bioactive formulations targeting skin rejuvenation [117,119,120,121,122,123,124]. Sericin hydrogels hold significant potential in the food sector for active packaging, nutrient delivery, and functional food development, aligning with trends toward natural, sustainable, and health-promoting ingredients [126]. With continued research and formulation refinement, sericin-based hydrogels hold substantial potential as multifunctional biomaterials across clinical and aesthetic domains.

Challenges and Future Perspectives

Some challenges persist in the development and application of sericin hydrogels. One major limitation is their relatively weak mechanical strength, which restricts their use in load-bearing or mechanically demanding TE applications. Additionally, variability in extraction and purification processes can result in batch-to-batch inconsistency in protein composition and performance. Addressing these limitations often requires blending sericin with other polymers or employing crosslinking strategies to enhance mechanical properties and ensure reproducibility [6,7,8,9,16,17,20,42,44,63,119,120,121,122,123].

#### 2.2.2. Plant-Derived Proteins

Soy protein isolate (SPI), pea protein, and wheat gluten are increasingly recognized as sustainable biomaterial candidates due to their inherent gelation capabilities, biocompatibility, and abundance. While their primary applications have been explored in the food industry, current research is extending their use toward the development of eco-friendly, biodegradable packaging and functional biomaterials. These proteins exhibit tunable viscoelastic properties and compatibility with green processing techniques, making them promising alternatives for reducing environmental impact and enhancing circular economy strategies [127,128].

SPI, pea protein, and wheat gluten are emerging as valuable sources of hydrogels with significant potential in biomedical applications. Their inherent gelation properties, biocompatibility, and sustainability make them ideal candidates for developing functional biomaterials that align with contemporary environmental goals. Continued research and innovation in this field will likely lead to the advancement of new, eco-friendly solutions that address both medical and environmental challenges [127,128].

##### Soy Protein Isolate-Based Hydrogels

Structural Properties

SPI is obtained from soybean, *Glycine max* (L.) Merr. (*Fabaceae*), and comprises approximately 90% protein by weight, consisting of a well-balanced amino acid composition that includes essential amino acids, such as leucine (~5.9%), lysine (~5.5%), methionine (~1.5%), threonine (~4.0%), and phenylalanine (~5.0%), as well as non-essential amino acids like alanine (~5.5%), aspartic acid (~9.5%), glutamic acid (~18.8%), glycine (~3.3%), and serine (~6.9%). Due to its high nutritional value, functional versatility, and sustainable origin, SPI has gained considerable attention in both food and biomedical sectors. This amino acid profile supports protein synthesis, cellular function, and tissue regeneration, making SPI an ideal biomaterial for hydrogel development in health-related applications [63,127,128].

Fabrication Methods

SPI is extracted from defatted soybean flour or meal. The process includes dissolving of soluble proteins and sugars in a mild alkali solution, separating the insoluble residue (mostly sugars) from the protein solution, precipitating the protein at its isoelectric point (pH~4.5), then separating, washing, neutralizing, and spray drying of highly purified SPI [63,127,128,129]. Various methods can be used for the fabrication of SPI hydrogels: (*i*) physical techniques using electric fields (anisotropic SPI hydrogels with distinctive pore structures), thermal aggregation (deamidated SPI), or freeze-thawing (high-strength SPI–epichlorohydrin (ECH) composite hydrogels); (*ii*) chemical crosslinking with *N*,*N*’-methylenebisacrylamide (MBAm), GAD, ECH, transglutaminase (TG); and (*iii*) combining with other polymers like κ-carrageenan, hydroxypropyl chitosan, PVA or poly(acrylic acid) (PAA) to create composite hydrogels with enhanced properties (good water absorption capacity, mechanical strength, and pH sensitivity) [130,131].

Biomedical Applications

The applications of SPI hydrogels span both the biomedical and food industries. In biomedical contexts, SPI hydrogels are being explored for wound healing, offering a moist environment conducive to TR while also enabling controlled drug delivery. Recent studies have demonstrated the potential of SPI hydrogels as biocompatible wound dressings that promote cell proliferation and accelerate healing, further underscoring their multifunctional utility [63,127,128,132]. In the food sector, SPI-based hydrogels are used to enhance the texture and nutritional profile of meat analogs, such as plant-based burgers, where they simulate the mouthfeel and juiciness of animal-derived products [127,128,132].

Challenges and Future Perspectives

Key advantages of SPI-based hydrogels include excellent biocompatibility and hydrophilicity. SPI is non-toxic, does not provoke significant immune responses, and can be safely used in biomedical systems such as wound healing and drug delivery. Its hydrophilic nature contributes to superior water absorption and retention, properties that are essential for hydrogel networks, especially in moisture-dependent applications like tissue scaffolds and wound dressings. However, certain challenges are associated with the formulation of SPI-based hydrogels. Gelation often requires tightly controlled pH and temperature conditions, which may limit the scalability and flexibility of processing methods. Additionally, some SPI preparations exhibit off-flavors or beany odors, potentially affecting consumer acceptance in food products or topical biomedical applications. Techniques such as enzymatic treatment, blending with other polymers, or deodorization processes are often employed to address these limitations [63,127,128].

##### Pea Protein-Based Hydrogels

Structural Properties

Pea protein, derived from yellow split peas, *Pisum sativum* L. (*Fabaceae*), is increasingly recognized for its high protein content, balanced amino acid composition, and environmental sustainability, positioning it as a valuable alternative to animal-based proteins [127,133,134,135,136]. Pea protein contains a wide range of essential amino acids such as leucine (~7.0%), lysine (~6.4%), methionine (~1.3%), threonine (~4.2%), and phenylalanine (~5.9%), which are critical for protein synthesis, immune function, and cellular metabolism [133,134,135,136]. Additionally, its non-essential amino acids, including alanine (~6.0%), aspartic acid (~10.2%), glutamic acid (~18.0%), glycine (~4.2%), and serine (~6.1%), contribute to energy production, nitrogen metabolism, and overall nutritional value [133,134,135].

Fabrication Methods

Fabrication of pea protein involves several steps: (*i*) a flour containing soluble fibers, starches, and proteins is obtained by mechanically removing the outer shell of the pea; (*ii*) separation of pea protein through wet filtration and centrifugation; (*iii*) precipitation of pea protein at the isoelectric point and purification to obtain a high-protein content (85–90%) isolate [137]. Pea protein hydrogels are fabricated through different methods including pH-shifting, TG-induced crosslinking, freeze-thawing, high-pressure processing, microparticulation, combining with other biomaterials such as sodium alginate, κ-carrageenan, inulin, gellan to enhance gelation, network strength, stability, and textural properties [135,137].

Biomedical Applications

Pea protein-based hydrogels have demonstrated potential in various applications. In the nutraceutical and pharmaceutical sectors, they are used for the encapsulation and controlled release of bioactive compounds, improving bioavailability and protecting sensitive molecules from degradation. Emerging studies also highlight the potential of pea protein hydrogels in biomedical applications, including wound dressings and scaffold materials, due to their biocompatibility, biodegradability, and low immunogenicity. In the food industry, they enhance the texture and protein content of plant-based meat analogues and functional foods [133,134,135,136,138,139].

Challenges and Future Perspectives

The functional properties of pea protein, including water-holding capacity, emulsifying ability, and gel-forming behavior, make it particularly suitable for the development of hydrogel systems. Its relatively high proportion of branched-chain amino acids supports muscle growth and recovery, making it popular in sports nutrition and therapeutic foods. Pea protein is also hypoallergenic and easily digestible, making it a suitable choice for individuals with dairy or soy allergies and for pediatric and geriatric nutritional products. Despite its advantages, certain limitations must be addressed. Pea protein hydrogels may exhibit an undesirably gritty texture, which can affect consumer acceptance in food applications. Additionally, sensitivity to enzymatic or thermal hydrolysis during processing may impact its gelling capacity and structural stability. Optimizing processing conditions and combining pea protein with other biopolymers (e.g., polysaccharides) may help overcome these issues and enhance hydrogel performance [133,134,135,136].

##### Wheat Gluten-Based Hydrogels

Structural Properties

Wheat gluten is a plant-derived protein complex predominantly composed of two major protein fractions: glutenin, responsible for elasticity and strength, and gliadin, which imparts viscosity and extensibility [139]. While primarily known for its essential role in bread-making and dough formation, wheat gluten has gained growing attention in hydrogel development due to its favorable mechanical and structural characteristics [139,140,141]. The amino acid profile of wheat gluten includes essential amino acids such as leucine (~4.0%), lysine (~2.5%), methionine (~1.0%), threonine (~3.0%), and phenylalanine (~4.5%), along with a high concentration of non-essential amino acids such as alanine (~8.0%), aspartic acid (~14.0%), glutamic acid (~26.0%), glycine (~2.5%), and proline (~12.0%). Although it is not a complete protein due to its low lysine content, its high glutamic acid and proline content contribute to its unique viscoelastic behavior and cohesive network-forming ability [140,141,142].

Fabrication Methods

Wheat gluten is obtained by separating the gluten protein from wheat flour in a multi-step process, which includes as main steps: mixing with water (hydration of wheat flour), starch removal, gluten separation, drying at and low temperature (40 °C) and processing into various forms (powder, sheets, blocks) [143]. Several methods can be used for the fabrication of wheat gluten hydrogels: (*i*) dispersing wheat gluten in water and adjusting the pH of the suspension; (*ii*) incubation of the suspension at a controlled temperature (20–50 °C) for 30 min, then cooling for 12 h to facilitate hydrogel formation; (*iii*) using crosslinking agents to enhance mechanical properties and network stability; (*iv*) enzymatic hydrolysis and fractionation to obtain peptides that can self-assemble into hydrogels [144].

Biomedical Applications

In terms of applications, wheat gluten hydrogels are widely used in the food industry to create meat-like structures, such as seitan, which is valued for its fibrous, chewy texture similar to muscle tissue [140,141]. In biomedical research, wheat gluten has been investigated as a matrix for DDS, particularly for the encapsulation of poorly water soluble or hydrophobic drugs. Its ability to form sustained-release hydrogels presents a promising platform for therapeutic delivery, although further studies are required to assess biocompatibility and performance in vivo [145].

Challenges and Future Perspectives

One of the primary advantages of wheat gluten in hydrogel systems is its exceptional elasticity and mechanical strength, which stem from the disulfide bond-mediated crosslinking of glutenin subunits. These structural features allow for the formation of robust and resilient hydrogels suitable for both food and biomedical applications. Furthermore, its viscoelastic properties make it a desirable material for mimicking the texture and chewiness of animal-based products in plant-based meat analogues. Wheat gluten’s ability to form a stable matrix also facilitates the encapsulation of active agents, offering opportunities in functional material design. However, there are notable challenges associated with wheat gluten-based systems. A major concern is its allergenic potential and gluten intolerance, which restricts its use among individuals with celiac disease or non-celiac gluten sensitivity. Additionally, the reactivity of wheat gluten to enzymatic and thermal hydrolysis can affect the reproducibility and performance of gluten-based hydrogels. Therefore, careful control of processing conditions, such as pH, temperature, and enzymatic treatment, is essential to ensure desirable gelation and stability for specific applications [135,136,138,139,140,141,142,145].

### 2.3. Polysaccharide-Based Hydrogels

Natural polysaccharides, such as chitin, chitosan, HA, alginate, carrageenan (CG), cellulose, starch, xanthan gum (XG), dextran (Dex), and pullulan, with many possible shapes and characteristics of the structure and with significant biological properties, are part of the category of natural polymers and have in their structure a sequence of carbohydrate monomers linked by glucose. Due to their exceptional biological properties, natural polysaccharides are intensively studied for their various applications, especially in medicine and pharmacy. Not long ago, hydrogels obtained based on natural polysaccharides showed particular interest in their biomedical applications. A series of natural polymers is the basis of natural hydrogels. An important characteristic of them is that they retain their designed properties upon contact with blood and, as drug carriers, they can be delivered either subcutaneously, orally, or intramuscularly. In contrast to synthetic hydrogels, natural ones obtained based on polysaccharides are superior in that they are easy to prepare and model as well as in structure, they can be obtained from many natural precursors at low cost, they are not toxic, they have good biocompatibility and biodegradability, and their physical and chemical properties are optimal for applications [2,10,11,12,13]. This category of hydrogels is widely utilized in hydrogel synthesis due to their biocompatibility, biodegradability, and low toxicity. Key examples include chitin, chitosan, HA, alginate, starch, CG, and cellulose. Although polysaccharides are very suitable for obtaining hydrogels, several properties need to be improved, such as antimicrobial activity and mechanical strength. Overcoming these technological challenges requires investment in research to develop ecological synthesis methods, the use of non-toxic solvents, and the identification of new crosslinking, functionalization, and combination procedures. Thus, they could become interesting for biomedical applications in DDS, delivery by injection, TE, or wound repair, and “smart” hydrogels [6,7,8,9,15,16,17,18,19,20,21,63,64,65,66,67,69,76].

#### 2.3.1. Animal-Derived Polysaccharides

##### Chitin-Based Hydrogels

Structural Properties

Chitin is a naturally abundant polysaccharide found predominantly in the exoskeletons of arthropods such as crabs, shrimp, and insects, as well as in the cell walls of fungi and certain algae It is composed of β(1→4)-linked *N*-acetyl-D-glucosamine (GlcNAc) units, forming a linear chain similar to cellulose, but with an acetylated amino group replacing the –OH group on the second carbon. The structural diversity of chitin is characterized by its polymorphic forms: α-chitin (most prevalent, found in crustaceans, with tightly packed antiparallel chains), β-chitin (present in squid pens and certain fungi, with parallel chains allowing for greater hydration and enzymatic accessibility), and the rarer γ-chitin (a mixture of both arrangements). These crystalline forms influence the polymer’s physicochemical behavior and reactivity. The molecular weight of chitin, which typically ranges from several thousand to several million Da, also significantly affects its solubility and mechanical properties, with higher molecular weight variants being particularly favorable for hydrogel development due to improved structural integrity [9,10,11,65].

Fabrication Methods

Chitin’s transformation into hydrogel form involves various gelation strategies that enhance its solubility, stability, and functionality. Since native chitin is poorly soluble in water, chemical modifications, such as partial deacetylation to produce chitosan or functionalization through grafting reactions, are often required to facilitate hydrogel formation. These modified chitin derivatives can undergo chemical crosslinking using agents like GAD or carbodiimide to yield covalently bonded networks with enhanced durability. Physical crosslinking, involving hydrogen bonding and van der Waals forces, allows for reversible gel formation under controlled pH or temperature conditions. Enzymatic crosslinking using biocompatible enzymes like laccase or TG offers a green synthesis pathway, while ionic gelation, particularly with divalent ions (e.g., Ca^2+^), provides mild and tunable crosslinking options. These diverse methods enable the design of hydrogels with tailored characteristics for specific biomedical and industrial applications [9,10,11,65,82,83,84].

Biomedical Applications

Chitin-based hydrogels exhibit a unique combination of properties that make them attractive for various applications. They are biocompatible, non-toxic, and biodegradable, degrading into naturally occurring monosaccharides that can be metabolized by environmental or host microorganisms. Their mechanical properties, such as elasticity and tensile strength, are highly tunable through crosslinking density, polymer blending, and molecular weight adjustment. These hydrogels demonstrate notable swelling capacity, allowing them to absorb large volumes of water, which is advantageous for moisture retention in wound healing or drug release systems. Moreover, chitin and its derivatives, particularly chitosan, possess inherent antimicrobial activity, attributed to their polycationic nature, which disrupts microbial cell membranes. These characteristics also extend their use beyond biomedicine into food preservation, cosmetics (e.g., skin moisturization), and agriculture, where they function as soil conditioners improving water retention and nutrient delivery [9,10,11,17,18,19,20,65].

In drug delivery, chitin-based hydrogels serve as effective carriers for a wide range of therapeutic agents, offering controlled and sustained release profiles. Functionalized hydrogels, such as those combining chitin with tamarind seed polysaccharides, have demonstrated enhanced drug encapsulation efficiency and hydration capacity, making them suitable for hydrophilic DDS and wound healing. Additionally, chitin-derived spheroidal hydrogels allow real-time monitoring of drug release, holding promises for targeted therapies and biosensor platforms. In TE, chitin hydrogels support cellular adhesion, proliferation, and differentiation. Their nanofibrous structure enhances mechanical properties and mimics the ECM, facilitating applications in skin, cartilage, and bone regeneration. Composite systems like chitin–collagen hydrogels further improve bioactivity and structural stability, making them excellent candidates for scaffold fabrication and 3D bioprinting applications [9,10,11,17,18,19,20,65,146].

Challenges and Future Perspectives

Chitin-based hydrogels are particularly promising in wound healing due to their moisture-retentive, absorbent, and antimicrobial properties. These characteristics foster an optimal healing environment by preventing dehydration and reducing infection risks. Some formulations, enhanced with natural or synthetic antimicrobial agents, accelerate tissue regeneration and epithelialization. Beyond wound care, their roles in cosmetic formulations and environmentally friendly agricultural products illustrate their versatility. To this end, chitin-based hydrogels are emerging as multifunctional platforms with broad potential across biomedical, cosmetic, food, and agricultural sectors [9,10,11,17,18,19,20,65]. Continued advancements in chitin modification, composite formulation, and crosslinking technologies are expected to expand their application spectrum, offering sustainable and effective solutions for future biotechnological innovations.

##### Chitosan-Based Hydrogels

Structural Properties

Chitosan, a biopolymer derived from the deacetylation of chitin, abundantly found in the exoskeletons of crustaceans and the cell walls of fungi, has garnered significant attention due to its biocompatibility, biodegradability, and low toxicity. Structurally, chitosan is composed of β-1,4-linked D-glucosamine and *N*-acetyl-D-glucosamine units, with its physicochemical properties largely influenced by the degree of deacetylation (DD) and molecular weight. A higher DD enhances solubility in acidic environments due to the presence of protonated amino groups, which also confer a cationic charge that facilitates electrostatic interactions with negatively charged molecules. Molecular weights can range from 50 to 1000 kDa, impacting viscosity, gelation, and biological performance. These features make chitosan an attractive candidate for biomedical applications, particularly in DDS, wound healing, and TE, where customizable structural and functional attributes are crucial [6,7,8,9,44,45,63,76,147,148,149].

Fabrication Methods

Chitosan-based hydrogels are synthesized through various gelation methods, which dictate their final properties. Physical gelation is driven by hydrogen bonding and chain entanglement, induced by changes in temperature, pH, or ionic strength. Chemical crosslinking employs agents like GAD, GNP, or citric acid to enhance network stability and mechanical strength [82,83,84]. Ionotropic gelation involves ionic interactions between chitosan and polyanions such as sodium tripolyphosphate (STPP), forming stable hydrogel matrices. Additionally, microwave-assisted and freeze–thaw techniques have emerged as innovative strategies for tuning porosity, gelation kinetics, and mechanical properties [147,148,149,150,151]. The resulting hydrogels possess high water retention, desirable viscoelasticity, and structural flexibility, properties that are further modulated by chitosan’s concentration, DD, molecular weight, and formulation conditions [6,7,8,9,21,29,44,45,63,76,147,148,149,150].

Polysaccharide-based hydrogels, especially those from sources like chitosan, alginate, HA, and cellulose derivatives, are widely used in BME due to their biocompatibility, biodegradability, and tunable properties. Recent innovations like 3D bioprinting, enzyme-assisted crosslinking, and NP integration are radically enhancing how these hydrogels are fabricated and applied [152,153,154].

3D bioprinting of polysaccharide hydrogels creates complex, customizable structures for DDS, TE, and wound healing. The hydrogel acts as a bioink that can be extruded, inkjetted, or laser-printed into layered structures. Chitosan is often blended with other materials (e.g., gelatin, alginate, PEG) to improve printability, viscosity, and cell viability. Challenges addressed chitosan’s pH sensitivity and poor solubility at physiological pH that can be tackled using derivatives (e.g., carboxymethyl chitosan) or co-printing with other bioinks. Recent applications include bioprinted organs-on-chips for drug testing, cartilage and skin tissue scaffolds, and wound dressings with embedded antimicrobial agents [152,153,154].

Enzyme-assisted crosslinking achieves mild, biocompatible, and site-specific crosslinking without toxic reagents. Several common enzymes are used, such as TG (crosslinking of proteins within the matrix), horseradish peroxidase (HRP)/hydrogen peroxide (H_2_O_2_) for catalyzing phenol or tyramine-functionalized polysaccharides, and laccase (oxidation of catechol groups in modified chitosan). Benefits include physiological conditions (e.g., neutral pH, low temperature), improved cell viability and encapsulation for TE, enabling the use of injectable hydrogels. Chitosan modified with tyramine or catechol groups can be crosslinked using HRP/H_2_O_2_, resulting in in situ gelation [152,153,154,155].

NP integration enhances mechanical strength, drug delivery, antibacterial, or stimuli-responsive properties. There are several types of NPs integrated: silver nanoparticles (AgNPs) for antibacterial activity, magnetic (magnetite—Fe_3_O_4_) NPs for remote control drug delivery or imaging, clay nanosheets (e.g., Laponite) for mechanical reinforcement, carbon-based nanomaterials (e.g., graphene oxide (GO)) for electrical conductivity and strength, polymeric NPs (e.g., poly(lactic-*co*-glycolic) acid (PLGA)- or PEG-based) for controlled drug release. NPs are mixed into the polysaccharide solution before or during gelation. Sometimes, they are surface modified to improve compatibility. Such pH-responsive hydrogels (pHRHs) or TRHs, triggered by environmental changes, and dual or multi-responsive systems are used for precision medicine [152,153,154].

Biomedical Applications

Chitosan-based hydrogels also hold value across multiple domains [6,7,8,9,29,63,147,148,149,150,151,155,156,157,158,159,160,161]. In wound care, their antimicrobial nature and moisture retention accelerate healing, with formulations such as chitosan–AgNPs hydrogels showing enhanced pathogen resistance [162,163]. Chitosan–fucoidan composites have demonstrated improved healing in diabetic wounds due to synergistic anti-inflammatory effects [164]. Beyond biomedicine, cosmetic applications benefit from their hydrating and skin-repairing properties [147,165], while in the food industry, chitosan hydrogels are utilized in biodegradable packaging to preserve product quality [3,86,147]. In agriculture, they improve soil moisture and nutrient availability, promoting plant health [6,7,8,9]. With ongoing research exploring novel polymer blends and crosslinking techniques, chitosan-based hydrogels are poised to become central to the next generation of sustainable and functional biomaterials.

Challenges and Future Perspectives

The physicochemical and biological properties of chitosan hydrogels include excellent biocompatibility, biodegradability, mucoadhesiveness, and antimicrobial activity. Their high water absorption capacity helps maintain a moist wound environment, essential for accelerated healing and tissue regeneration. These hydrogels also serve as effective drug delivery platforms due to their ability to encapsulate and sustain the release of hydrophobic and hydrophilic agents [6,7,8,9,29,63,147,148,149,150,151,155]. For example, chitosan–sodium alginate hydrogels have been shown to deliver anticancer drugs with controlled release profiles [147,148,149,150,151]. In TE, composite systems such as enhance fibroblast proliferation, support skin regeneration and enable targeted neuro-drug delivery [156,157,158], while chitosan, GO hybrids enhance mechanical integrity and conductivity, offering potential for nerve regeneration [159,160,161].

##### Hyaluronic Acid-Based Hydrogels

Structural Properties

HA is a naturally occurring linear glycosaminoglycan that is widely distributed in connective, epithelial, and neural tissues, where it plays essential structural and physiological roles. Structurally, HA is composed of repeating disaccharide units of *N*-acetyl-D-glucosamine and D-glucuronic acid, connected via alternating β-1,4 and β-1,3 glycosidic linkages. As a key component of the ECM, HA contributes to tissue hydration, scaffolding, and cellular communication. Its molecular weight, which can range from 5 kDa to over 6000 kDa, significantly influences its viscoelastic behavior, degradation kinetics, and biological activity [31,65,66,67,92,112,113,166].

Fabrication Methods

Various gelation mechanisms enable the formation of HA-based hydrogels tailored to specific applications. Physical gelation involves reversible interactions such as hydrogen bonding and hydrophobic forces, influenced by pH, temperature, or ionic strength. Ionic gelation occurs through interactions with multivalent cations like Ca^2+^, forming reversible crosslinks. Chemical crosslinking with agents like GAD, EDC/NHS, or citric acid generates covalent bonds for increased stability, while photo-crosslinking allows for precise spatiotemporal gelation using UV light and photoinitiators. Enzymatic crosslinking, using enzymes such as HRP or tyrosinase, provides a gentle, in situ approach to hydrogel formation compatible with physiological environments. The resulting hydrogels exhibit high water content, excellent biocompatibility, and tailorable mechanical properties. Their swelling ability is advantageous for maintaining a moist wound environment, and their degradation by hyaluronidase enzymes results in non-toxic, bioresorbable byproducts [31,65,66,67,81,82,83,84,91,92,112,113,166,167].

Biomedical Applications

The biomedical utility of HA is underpinned by its hydrophilicity, allowing it to retain water up to 1000 times its own weight, thereby preserving tissue hydration and elasticity. Its viscoelastic properties make it suitable for load-bearing applications like synovial joints. HA’s interactions with cell surface receptors, such as cluster of differentiation 44 (CD44), a transmembrane glycoprotein that serves as a primary receptor for HA, receptor for HA-mediated motility (RHAMM), and others, promote cell adhesion, migration, and proliferation, reinforcing its role in tissue regeneration and wound healing. In cosmetic applications, HA is extensively used in dermal fillers and skincare formulations for its skin-plumping and wrinkle-reducing effects. In ophthalmology, it functions in eye drops for dry eye syndrome and as a viscoelastic agent in cataract surgery. In orthopedics, intra-articular HA injections restore joint lubrication and relieve pain in osteoarthritis. These hydrogels support essential cellular activities such as adhesion, migration, and proliferation, making them ideal for use in RM. By modifying the molecular weight or crosslinking density, properties like stiffness and elasticity can be precisely tuned for specific therapeutic contexts [31,65,66,67,81,82,83,84,91,92,112,113,166,167].

In drug delivery, HA hydrogels offer a promising platform for localized, sustained re-lease of therapeutic agents. Hybrid systems like HA–alginate hydrogels employ ionic crosslinking to enhance network stability and have demonstrated effectiveness in controlled anticancer drug delivery, reducing systemic toxicity. Functionalization of HA with targeting ligands or NPs allows for site-specific delivery, particularly to tumor cells overexpressing CD44 receptors [168]. In TE, combinations like HA–gelatin hydrogels enhance cell adhesion and support dermal regeneration [169], while photocurable HA–PEG hydrogels provide injectability and mechanical integrity for cartilage repair [170]. HA–PLGA composites promote osteoblast activity and bone healing [170]. In wound care, HA hydrogels maintain hydration and act as microbial barriers [166,167], while innovations such as HA–AgNPs hydrogels offer potent antimicrobial activity for chronic wound management [171].

Challenges and Future Perspectives

Thanks to its intrinsic biocompatibility, hydrophilicity, viscoelasticity, and biodegradability, HA has gained prominence as a versatile biomaterial in BME. Its physicochemical properties can be finely tuned by altering the polymer’s molecular weight or concentration, thereby adjusting mechanical strength, degradation rate, and swelling characteristics. These attributes make HA an excellent candidate for hydrogel fabrication, where it serves as a soft, ECM-mimetic matrix capable of sustaining cellular viability and biofunctionality, especially in regenerative therapies. However, native HA hydrogels have limitations, particularly their mechanical fragility and susceptibility to enzymatic degradation, which constrain their use in load-bearing tissues or long-term applications. To overcome these drawbacks, various chemical modifications such as sulfation, methacrylation, and conjugation with hydrophobic moieties are employed to enhance stability and functionality. Additionally, multiple crosslinking strategies, including physical, ionic, chemical, enzymatic, and photo-crosslinking, are applied to reinforce the hydrogel network, enabling better control over structure and longevity [31,65,66,67,82,83,84,92,93,112,113,166,167].

#### 2.3.2. Plant-Derived Polysaccharides

##### Alginate-Based Hydrogels

Structural Properties

Alginate is a naturally derived, linear polysaccharide composed of β-D-mannuronic acid (M) and α-L-guluronic acid (G), primarily extracted from brown seaweeds (*Phaeophyceae*). These monomers are arranged in homopolymeric (MM or GG) or heteropolymeric (MG) blocks, and their distribution directly influences the physicochemical properties of alginate. G-blocks contribute to gel strength and rigidity, while M-blocks provide flexibility. The molecular weight of alginate varies based on its source and extraction method, typically ranging from several tens of thousands to over a million Da. Its high molecular weight is associated with improved viscosity and gel-forming capacity, which are advantageous in many industrial and biomedical applications. Due to its excellent biocompatibility, biodegradability, hydrophilicity, and high water absorption capacity, alginate is widely employed in food, pharmaceutical, cosmetic, and biomedical sectors [6,7,8,9,41,63,64,65,66,67,69,172,173,174,175,176,177,178,179,180,181].

Fabrication Methods

Alginate readily forms hydrogels through multiple crosslinking strategies, enabling tailored material properties for specific applications. The most prevalent method, ionotropic gelation, involves the interaction of alginate with divalent cations such as calcium, forming “egg-box” junctions between G-blocks. This allows for rapid gelation under mild, biocompatible conditions. In addition to ionic crosslinking, chemical agents like GAD or PEG derivatives can be used to generate covalent networks with enhanced mechanical stability and prolonged degradation profiles. Physical gelation via changes in temperature or pH, though reversible and less stable, is also feasible. Moreover, enzymatic crosslinking, such as through TG, enables site-specific, biocompatible gelation suitable for biomedical use [6,7,8,9,41,63,64,65,66,67,69,82,83,84,172].

Alginate is a standout among natural polymers for hydrogel fabrication, especially in biomedical applications due to its biocompatibility, gentle gelation, and ease of functionalization. Recent innovations like 3D bioprinting, enzyme-assisted crosslinking, and NP integration are driving major advancements in how alginate-based hydrogels are designed and applied [182,183,184].

In situ 3D bioprinting of alginate hydrogels allows rapid ionic gelation and mild processing conditions that protect cells and biomolecules. The main challenges refer to weak mechanical strength and lack of cell-adhesive sites. Blending with gelatin, collagen, or cellulose for bioactivity and NPs or synthetic polymers for strength are the solutions applied to solve the challenges. Techniques include extrusion-based printing (most common) and multi-material printing with spatial control over bioactive agents. High-throughput drug screening and bioprinted tissues (e.g., cartilage, vascular grafts), organoids, and tumor models represent the main applications. Alginate-gelatin blends are printed at 37 °C, then crosslinked with calcium chloride spray post-printing for scaffold stability and cell support [182,183,184].

Because traditional Ca^2+^ crosslinking can be rapid and uncontrolled, enzyme-assisted crosslinking allows for gradual, uniform gelation with biological compatibility. Main enzymes used are represented by tyrosinase/HRP for crosslinking of phenol-functionalized alginate, and TG, if alginate is blended with proteins like gelatin. Enhanced spatiotemporal control over gelation, tunable mechanical properties, and the possibility of injectable formulations are the most important benefits. Alginate modified with tyramine is crosslinked enzymatically via HRP/H_2_O_2_, forming a stable, injectable hydrogel for drug delivery or tissue repair [182,183,184].

NP integration improves mechanical, electrical, or biological properties and enables stimuli-responsive behaviors (pH, temperature, magnetic field). Specific types of NPs are used for drug-loading capacity (mesoporous silica (SiO_2_) NPs), remote-controlled release and MRI contrast (Fe_3_O_4_ NPs), antibacterial wound dressing (AgNPs), mechanical reinforcement (clay nanosheets—Laponite), and electrical conductivity of neural scaffolds (graphene, CNTs). Approaches include dispersing of NPs in alginate pre-gel solution, crosslinking as usual (ionically or enzymatically), and optimizing of NP surface chemistry for better dispersion and interaction. Alginate–Ag nanocomposite hydrogels for antibacterial wound care show enhanced strength, slow Ag^+^ ion release, and high biocompatibility [182,183,184].

Biomedical Applications

Owing to these tunable properties, alginate hydrogels have been successfully applied in diverse biomedical domains [172,173,174,175,176,177,178,179,180,181]. In DDS, ionically crosslinked alginate–Ca^2+^ systems have demonstrated sustained release capabilities for molecules like insulin, relevant for chronic disease management [127,185]. For TE, composite hydrogels such as alginate–gelatin [79,127,172] and alginate–PEG offer scaffolding with tailored cell-adhesive, proliferative, and differentiation-supportive environments, particularly for cartilage, skin, and bone regeneration [28,101,172]. Innovations such as alginate–GO composites provide controlled release, mechanical reinforcement, and electrical conductivity, useful for nerve TR and biosensing [177,178]. Furthermore, alginate–chitosan hydrogels are noted for their moist environment maintenance and antimicrobial performance, leading to improved healing rates in diabetic models [179,186] and promising candidates for spinal cord injury [181].

Beyond biomedical applications, alginate hydrogels are extensively used in food, cosmetics, and agricultural industries. In food technology, they function as thickeners, stabilizers, and gelling agents for improved texture and shelf life. Edible films and coatings based on alginate enhance moisture retention and serve as barriers against spoilage [172,181]. In cosmetics, alginate hydrogels are incorporated into facial masks and skin treatments for their hydrating and film-forming properties, promoting active ingredient delivery [172]. Agriculturally, alginate-based hydrogels are applied to soil for water retention and nutrient delivery, reducing irrigation needs and enhancing sustainability [172].

Challenges and Future Perspectives

The key material properties of alginate hydrogels make them ideal for multifunctional applications. Their biocompatibility ensures minimal cytotoxicity, supporting their use in tissue interfaces. Alginate hydrogels are biodegradable, typically degraded by alginate lyases or ion exchange in physiological fluids, making them favorable for transient implants or controlled drug release. Mechanical properties such as elasticity and compressive strength can be modulated by varying polymer concentration, molecular weight, and crosslinking density. Their swelling capacity allows high water uptake, facilitating use in wound dressings and moisturizing formulations. Furthermore, alginate’s ability to encapsulate bioactive agents and release them in a controlled manner has driven its use in drug delivery platforms [6,7,8,9,41,63,64,65,66,67,69,172,173,174,175,176,177,178,179,180,181]. Collectively, the multifunctionality and adaptability of alginate hydrogels underscore their significance in advancing material science and addressing interdisciplinary challenges in health, food, and environmental sectors.

##### Carrageenan-Based Hydrogels

Structural Properties

CG is a naturally occurring sulfated polysaccharide derived from red algae (*Rhodophyceae*), particularly species within the *Chondrus* (Irish moss), *Kappaphycus*, and *Gigartina* genera. Structurally, it is a linear galactan composed of alternating *β*-D-galactose and *α*-D-galactose (or 3,6-anhydro-D-galactose) residues, heavily substituted with sulfate groups. The degree and position of sulfation define the three major types of CG: kappa (κ), iota (ι), and lambda (λ). κ-CG forms strong, brittle gels in the presence of K^+^ ions, while ι-CG produces softer, more elastic gels with Ca^2+^ ions. λ-CG, characterized by a higher degree of sulfation, does not form gels but functions effectively as a thickening agent. The molecular weight of CG can vary significantly, from 50,000 to several million Da, depending on the extraction and purification processes, impacting its rheological and gelation properties [6,7,8,10,41,63,64,65,66,67,69].

Fabrication Methods

CG-based hydrogels can be formed through several gelation mechanisms, including ionic crosslinking, thermal gelation, pH modulation, and enzymatic reactions. Ionic gelation, particularly prominent in κ-CG and ι-CG, involves the formation of helical structures stabilized by the introduction of mono- or divalent cations. Thermal gelation occurs when CG solutions transition from sol to gel upon cooling, a reversible process that is advantageous for injectable systems. pH-induced gelation leverages the ionization state of functional groups to control network formation, while enzymatic crosslinking (e.g., using TG) enhances mechanical robustness and stability. These hydrogels are biocompatible and biodegradable, with tunable mechanical strength, swelling behavior, and diffusivity, key features for controlled drug delivery and BME applications [6,7,8,10,41,69,177,187,188,189,190].

Biomedical Applications

In the biomedical field, CG-based hydrogels are emerging as promising platforms for drug delivery, TE, and wound healing [6,7,8,10,41,63,64,65,66,69,187,188,189,190]. κ-CG hydrogels, known for their thermoreversible gelation and mechanical strength, have been utilized for the encapsulation of hydrophilic drugs like metformin, facilitating sustained and localized release [191]. CG–alginate composite hydrogels combine the ionic responsiveness of both polymers, enhancing structural integrity and expanding utility in cancer drug delivery [6,7,8,10,41,63,64,65,66,69,187,188,189,190]. ι-CG hydrogels have shown success as scaffolds for cell proliferation and GFs encapsulation in bone regeneration [192,193]. Furthermore, CG–collagen blends enhance fibroblast proliferation, making them suitable for skin regeneration and wound healing [194]. The addition of AgNPs imparts antimicrobial properties, resulting in composite hydrogels capable of accelerating wound healing while preventing infection, as demonstrated in multiple in vivo studies [194].

CG plays a significant role in food and cosmetic industries due to its multifunctional properties. κ-CG is widely used as a gelling and stabilizing agent in dairy products, meat emulsions, and desserts, enhancing textural and sensory qualities. ι-CG is utilized in processed foods to create elastic gels that resist syneresis, while λ-CG serves as a thickener in sauces and beverages. In cosmetics, CG-based hydrogels are incorporated into moisturizers, facial masks, and emulsions for their water-holding capacity and skin-soothing effects. λ-CG, in particular, has demonstrated superior skin hydration properties, contributing to improved product performance and consumer satisfaction. These attributes, combined with safety and regulatory acceptance, make CG a versatile ingredient across commercial formulations [6,7,8,10,21,41,63,64,65,66,69].

Challenges and Future Perspectives

CG-based hydrogels represent a versatile and sustainable class of biomaterials with demonstrated utility in food, cosmetic, and biomedical applications [6,7,8,10,63,64,65,66,187,188,189,190,191,192,193,194]. Their key advantages, biocompatibility, environmental degradability, and adjustable mechanical and rheological properties, make them ideal for use in next-generation DDS, scaffolding materials, and therapeutic wound dressings. Current research is increasingly focused on molecular tailoring, composite development (e.g., with alginate or collagen NPs), and biofunctionalization to expand their therapeutic potential [192,193,194]. The integration of advanced crosslinking techniques and nanostructured components is expected to enhance the performance of CG hydrogels, paving the way for innovative, eco-friendly solutions in healthcare and biotechnology [82,83,84].

##### Cellulose-Based Hydrogels

Structural Properties

Cellulose, the most abundant organic polymer on Earth, is a structural component of the cell walls in green plants, certain algae, and select bacteria. Composed of linear chains of β-D-glucose units connected via β-1,4-glycosidic bonds, cellulose exhibits notable properties such as biocompatibility, biodegradability, and non-toxicity. These attributes have driven substantial interest in its use for hydrogel development across biomedical, pharmaceutical, environmental, and food-related applications. Structurally, cellulose consists of repeating cellobiose units, each formed by two glucose molecules. The degree of polymerization (DP), ranging from hundreds to over 10,000 glucose units, significantly influences its mechanical properties and solubility. The crystalline organization of cellulose varies based on its source (e.g., cellulose I, II), affecting its swelling capacity and hydrophilic interactions, key parameters in hydrogel design. While cellulose’s abundant –OH groups confer hydrophilicity, chemical modification is often necessary to enhance its reactivity and functional compatibility for specialized uses [65,66,67,195,196,197].

Fabrication Methods

Cellulose-based hydrogels can be prepared via several routes. Physical crosslinking utilizes hydrogen bonding and van der Waals interactions to form reversible networks. Chemical crosslinking involves covalent bonding using agents such as GAD or ECH, leading to more stable and durable hydrogels. Solvent exchange methods involve dissolving cellulose in suitable solvents (e.g., ionic liquids, sodium hydroxide/urea systems) and regenerating it into a gel phase, forming highly hydrated networks [65,66,67,195,196].

Several cellulose derivatives have been developed to improve hydrogel performance. Carboxymethyl cellulose (CMC) is a water-soluble derivative known for its excellent thickening and stabilizing properties, especially in pharmaceuticals and food [65,66,67,195,196]. Hydroxypropyl methylcellulose (HPMC), another widely used ether derivative, serves as a film-former and drug delivery vehicle. Microcrystalline cellulose (MCC) enhances texture and stability in formulations [195,197]. Nanocellulose, in the form of cellulose nanofibers (CNFs) or cellulose nanocrystals (CNCs), offers superior mechanical strength, high surface area, and enhanced functionalization for advanced hydrogel applications [195,198].

Biomedical Applications

In biomedicine, cellulose-based hydrogels demonstrate notable potential. CMC hydrogels are used in wound dressings, maintaining a moist environment that promotes healing and facilitates the delivery of antimicrobial agents. HPMC hydrogels are widely employed in controlled DDS, where their viscoelastic properties allow for sustained release of therapeutic agents. Nanocellulose-based hydrogels offer ECM-like architecture suitable for TE, supporting cell adhesion, proliferation, and differentiation [65,66,67,195,196,197,198,199]. Beyond medicine, cellulose hydrogels play a critical role in food technology. Their thickening and stabilizing functions improve food texture and moisture retention. They are also used in edible films and coatings, providing barriers that extend shelf life and reduce microbial contamination. In environmental science, cellulose hydrogels aid in water purification by adsorbing pollutants, while in agriculture, they improve soil moisture retention and nutrient delivery, contributing to more sustainable farming practices [65,66,67,195,200,201].

Challenges and Future Perspectives

The key properties of cellulose-based hydrogels include high swelling capacity, allowing absorption of water many times their weight, an essential trait for applications like wound dressings and agricultural soil enhancers. Their inherent biocompatibility ensures minimal immune response, making them suitable for in vivo use. Moreover, their biodegradability promotes eco-friendliness, making them advantageous for temporary biomedical implants or sustainable environmental applications. Cellulose-based hydrogels are versatile, sustainable materials with expanding roles in biomedical, food, environmental, and agricultural applications [65,66,67,195,196,197,198,199,200,201]. Their tailored structure, biocompatibility, and degradability make them ideal candidates for next-generation “smart” materials. Ongoing research into new derivatives and fabrication methods continues to unlock their potential, especially in tissue regeneration, DDS, and eco-conscious technologies.

##### Starch-Based Hydrogels

Structural Properties

Starch is a naturally abundant polysaccharide composed predominantly of glucose units, and it serves as a primary energy reserve in plants. It exists mainly in two structural forms: amylose and amylopectin. Amylose consists of linear chains of glucose linked by α-(1→4) glycosidic bonds and typically adopts a helical conformation. Amylopectin, in contrast, has a highly branched structure, incorporating both α-(1→4) and α-(1→6) linkages. These two components are generally present in proportions of 20–30% amylose and 70–80% amylopectin, depending on the botanical source. The molecular weight of starch can vary substantially, from a few thousand to several million Da, affecting its physicochemical behavior. This variability plays a significant role in determining the rheological and mechanical properties of starch-based hydrogels. Due to its biocompatibility, biodegradability, and renewability, starch has garnered increasing interest for hydrogel development across pharmaceutical, biomedical, food, and agricultural applications [65,68,202].

Fabrication Methods

Starch forms hydrogels via several mechanisms, each yielding distinct structural and functional characteristics. Thermal gelation involves the swelling and gelatinization of starch granules in hot water, disrupting the crystalline regions and allowing amylose and amylopectin to leach out and form a gel network upon cooling. Chemical crosslinking, using agents such as GAD or ECH, strengthens gel networks by forming covalent bonds, thus enhancing thermal and mechanical stability. Physical crosslinking relies on non-covalent interactions like hydrogen bonding and hydrophobic interactions to produce gels without the use of chemical reagents. Additionally, enzymatic crosslinking, employing enzymes such as amylases, can modulate starch chains to produce hydrogels with tailored properties. Ionic gelation is another strategy in which divalent cations induce gelation in modified starches, especially in drug delivery applications where responsive swelling is crucial [65,68,82,83,84,202].

Biomedical Applications

Starch hydrogels have proven particularly effective in DDS due to their swelling capacity and tunable release profiles. Native starch hydrogels formed through thermal gelatinization are capable of encapsulating drugs like metformin, while chemically modified derivatives such as carboxymethyl starch enhance loading efficiency and bioavailability of agents like ibuprofen. These properties make starch hydrogels attractive platforms for sustained and targeted drug release. In TE, starch–agar hydrogels have demonstrated improved mechanical stability and compatibility with fibroblast proliferation, suggesting utility in soft tissue regeneration. These hydrogels serve as supportive scaffolds for cell attachment and growth, with potential applications in 3D bioprinting. For wound healing, starch–PVA composite hydrogels offer excellent moisture retention and mechanical resilience. Crosslinked hydrogels using agents like STPP show promise in promoting wound closure and tissue regeneration by maintaining a moist environment conducive to healing [65,68,202].

Beyond biomedical uses, starch-based hydrogels hold value across the food, cosmetic, and agricultural industries. In food technology, they function as thickeners, stabilizers, and moisture-retention agents, improving texture and shelf life. Their biocompatibility and hydrating properties also make them useful in cosmetic products such as facial masks. In agriculture, starch hydrogels enhance soil moisture retention and nutrient availability, reducing irrigation frequency and supporting sustainable crop management. As interest in biodegradable and renewable materials continues to rise, starch-based hydrogels are poised for expanded roles across disciplines [65,68,202].

Challenges and Future Perspectives

Starch-based hydrogels exhibit a suite of beneficial properties that support their multifunctionality. Their biocompatibility ensures minimal cytotoxicity, making them suitable for applications such as drug carriers and scaffolds. These hydrogels are biodegradable, naturally breaking down into glucose monomers that can be metabolized, ensuring low environmental impact. Their mechanical properties, such as elasticity and tensile strength, can be modulated by varying the amylose-to-amylopectin ratio or through chemical modifications. The swelling behavior of starch hydrogels, vital for controlled release, is influenced by the crosslinking density and starch type. Furthermore, their inherent hydrophilicity supports efficient water absorption, while controlled release capabilities enable the gradual and sustained delivery of bioactive agents, particularly for hydrophilic drugs [65,68,82,83,84,202]. Ongoing research into structural modifications, nanocomposite formulations, and hybrid systems will further optimize their functional performance and unlock novel applications in RM, “smart” packaging, and environmental remediation.

##### Xanthan Gum-Based Hydrogels

Structural Properties

XG is a natural, high-molecular-weight polysaccharide synthesized through the microbial fermentation of glucose or sucrose by the bacterium *Xanthomonas campestris* (Pammel 1895) Dowson 1939 (*Xanthomonadaceae*). Structurally, XG consists of a cellulosic backbone of β-D-glucose units linked by β(1→4) glycosidic bonds, with trisaccharide side chains comprising mannose, glucuronic acid, and a second mannose residue attached via α(1→3) and α(1→6) linkages. This repeating pentasaccharide unit, (D-glucuronic acid)–(D-glucose)–(D-mannose)–(D-glucose)–(D-mannose), contributes to the polymer’s high viscosity, stability, and ability to form gels. Additionally, XG contains multiple –OH functional groups, conferring it with significant hydrophilicity and water retention capacity. Its molecular weight typically ranges between 1–2 million D, a factor that enhances its rheological behavior and utility in hydrogel development [65,66,67,203].

Fabrication Methods

XG exhibits excellent rheological properties, including shear-thinning or pseudoplastic behavior, which allows it to flow under stress but retain viscosity at rest, an important feature in biomedical and food formulations. When processed into hydrogels, XG forms physically or chemically crosslinked networks that exhibit favorable characteristics such as biocompatibility, biodegradability, high water absorbency, and thermal and pH stability. It can interact with divalent cations (e.g., Ca^2+^) or be blended with other polymers to enhance gelation and mechanical integrity. Furthermore, XG is amenable to chemical modification, such as grafting or crosslinking with bioactive molecules, which can tailor its bioactivity for specialized functions in DDS and TE [65,66,67,203].

Biomedical Applications

The biomedical relevance of XG-based hydrogels is vast, with key applications in drug delivery, wound healing, TE, and ophthalmology. These hydrogels are capable of encapsulating and releasing both hydrophilic and hydrophobic drugs in a controlled manner, offering advantages in the sustained release of therapeutic agents, such as anticancer drugs. In wound care, XG hydrogels support healing by maintaining a moist environment and can be functionalized with antimicrobial agents like AgNPs. In TE, XG serves as a scaffold that supports cell adhesion and proliferation, particularly in applications like cartilage regeneration. Additionally, its viscoelastic properties make it ideal for ophthalmic formulations, where it enhances moisture retention and comfort in products like artificial tears and eye drops [65,66,67,203].

Beyond biomedical uses, XG-based hydrogels are integral to the food and cosmetic industries. In food technology, they function as thickening and stabilizing agents in products such as dressings, sauces, and gluten-free baked goods, where they mimic the viscoelastic properties of gluten. These hydrogels can also be engineered to deliver active ingredients in cosmetics, acting as moisturizing agents or carriers of skin-beneficial compounds. Their natural origin and regulatory acceptance as food-grade polymer further broaden their commercial appeal in clean-label and eco-friendly product lines [65,66,67,203].

Challenges and Future Perspectives

Despite their versatility, XG-based hydrogels face several challenges that limit their broader application. These include relatively low mechanical strength compared to synthetic hydrogels, sensitivity to ionic concentration which can affect gel consistency, and variability in viscosity depending on environmental conditions such as pH and temperature. Additionally, their degradation rates can be inconsistent, and chemical modifications often require complex procedures and specialized equipment. Addressing these limitations through advanced formulation strategies, polymer blending, or nanocomposite integration is essential for enhancing their functional performance. Continued research and innovation in this area are expected to expand the utility of XG-based hydrogels in next-generation biomedical, food, and personal care systems [65,66,67,203].

##### Dextran-Based Hydrogels

Structural Properties

Dex is a naturally occurring extracellular polysaccharide synthesized primarily by lactic acid (LA) bacteria, notably *Leuconostoc mesenteroides* (Tsenkovskii 1878) van Tieghem 1878 (*Lactobacillaceae*), through the fermentation of sucrose. It comprises predominantly α-D-glucopyranosyl units connected by α(1→6) glycosidic bonds, with occasional α(1→3) branching. This highly branched structure imparts Dex with excellent water solubility and unique viscoelastic properties. The general molecular formula of Dex can be approximated as C_n_H_2n_O_n_, where *n* corresponds to the number of repeating glucose units. The presence of numerous –OH groups not only contributes to its pronounced hydrophilicity but also offers reactive sites for chemical modifications, facilitating the design of functional hydrogels [65,66,67,204].

Fabrication Methods

Dex-based hydrogels are known for their high water retention, biocompatibility, and tunable mechanical and degradation properties. Their hydrophilicity supports moisture retention and creates favorable conditions for cellular activities in TE applications. Although native Dex is not inherently biodegradable, modifications, such as crosslinking with biodegradable moieties or enzymatically cleavable linkers, can impart controlled biodegradation. Furthermore, Dex hydrogels can be engineered to exhibit thermoresponsive behavior, enabling sol–gel transitions that facilitate injectable formulations for minimally invasive applications. Their mechanical strength and viscoelastic properties can be tailored by adjusting molecular weight (ranging from 1 kDa to over 500 kDa) and the DC [65,66,67,204].

Biomedical Applications

Dex-based hydrogels have shown considerable promise in several biomedical domains. In DDS, they serve as reservoirs for both hydrophilic and, with modification, hydrophobic drugs. For instance, doxorubicin-loaded Dex hydrogels have demonstrated sustained and localized release, enhancing anticancer efficacy while minimizing systemic toxicity. In TE, Dex hydrogels support chondrocyte growth and cartilage matrix synthesis, especially when functionalized with bioactive molecules like tyramine or peptides. Wound healing applications benefit from their moisture-retaining capacity and compatibility with antimicrobial agents; AgNPs–Dex hydrogels are particularly effective in treating chronic wounds. Moreover, Dex hydrogels are utilized in ophthalmology for contact lens hydration and artificial tear formulations. In emerging fields such as 3D bioprinting, Dex–methacrylate (DexMA) is used as a bioink to fabricate complex, cell-laden scaffolds [65,66,67,204,205,206].

Challenges and Future Perspectives

Despite their versatility, Dex-based hydrogels face several challenges that limit broader implementation. Their mechanical strength, while tunable, generally falls short of the requirements for load-bearing applications unless reinforced with other polymers. Achieving predictable degradation profiles can be complex due to dependency on environmental conditions and enzymatic activity. Additionally, Dex lacks native cell adhesion motifs, necessitating further functionalization for effective cell attachment and proliferation. The encapsulation efficiency for hydrophobic drugs remains suboptimal without chemical modifications or copolymers. From a translational perspective, the chemical processes required for hydrogel fabrication, such as photopolymerization or grafting, can be technically demanding, affecting scalability and reproducibility. Moreover, as with all biomaterials intended for clinical use, Dex-based hydrogels must meet rigorous regulatory standards, requiring extensive safety and efficacy evaluations. Dex-based hydrogels offer a compelling platform for a range of biomedical applications due to their customizable chemical structure, biocompatibility, and functional versatility. Continued efforts in chemical modification, hybrid hydrogel development, and advanced processing techniques, such as 3D bioprinting and injectable systems, are critical to overcoming current limitations. Future research aimed at integrating bioactive motifs, improving mechanical performance, and optimizing degradation kinetics will further enhance the translational potential of Dex hydrogels [65,66,67,204,205,206]. With growing interest in biopolymer-based therapeutic systems, Dex stands out as a promising candidate for the next generation of medical materials and regenerative technologies.

##### Pullulan-Based Hydrogels

Structural Properties

Pullulan is a natural, extracellular polysaccharide biosynthesized by the fungus *Aureobasidium pullulans* (de Bary) G. Arnaud (1918) (*Dothioraceae*) through fermentation of starch-derived sugars. Structurally, it comprises repeating maltotriose units, each formed by three glucose monomers connected via α(1→4) glycosidic bonds, linked together through α(1→6) bonds. This distinctive linkage imparts pullulan with a unique linear yet flexible structure, contributing to its high water solubility and film-forming ability. Its molecular weight can range from several tens of kDa to a few million Da, depending on fermentation conditions, which significantly influences its viscosity and gelation properties. Rich in –OH groups, pullulan is highly hydrophilic and readily modifiable, allowing it to serve as a versatile platform for hydrogel synthesis [65,66,67].

Fabrication Methods

Pullulan-based hydrogels are gaining significant interest in biomedical research due to their biocompatibility, non-immunogenicity, and biodegradability. These hydrogels can absorb large amounts of water, maintain a moist environment, and degrade enzymatically under physiological conditions. Their functional versatility is further enhanced through chemical modifications such as methacrylation or conjugation with bioactive molecules, enabling tunable mechanical properties, degradation rates, and responsiveness to external stimuli like temperature or pH. While native pullulan hydrogels may have limited mechanical strength, crosslinking with agents like GNP or blending with other polymers can improve their structural integrity and expand their range of applications [65,66,67,82,83,84].

Biomedical Applications

In DDS, pullulan hydrogels are employed as carriers for controlled and localized release of therapeutic agents. Their hydrophilic matrix allows for sustained diffusion of encapsulated drugs while protecting them from premature degradation. For instance, pullulan hydrogels have been used to deliver anticancer agents with prolonged release kinetics and improved bioavailability. Additionally, pullulan’s modifiability allows for targeted drug delivery by attaching ligands or NPs, making these hydrogels especially valuable in cancer therapeutics and chronic disease management [65,66,67,207].

In the area of wound healing, pullulan hydrogels offer numerous benefits, including moisture retention, oxygen permeability, and the potential to incorporate antimicrobial agents such as AgNPs. These properties create an optimal environment for tissue regeneration, reduce infection risk, and accelerate healing. Pullulan hydrogels have also been explored as scaffolds in TE, supporting cell adhesion, proliferation, and ECM synthesis. Functionalized variants have shown promise in engineering cartilage, skin, and vascular tissues, with their degradation profiles aligned to tissue remodeling timelines [65,66,67,207].

Beyond medical applications, pullulan hydrogels have been investigated for ocular drug delivery, where their transparency, mucoadhesiveness, and hydration capacity enhance drug residence time on the ocular surface [208]. They also show promise in food technology, where pullulan’s film-forming capacity is leveraged to create biodegradable edible films for nutrient encapsulation or as eco-friendly packaging alternatives to synthetic plastics. These applications are driven by pullulan’s Generally Recognized as Safe (GRAS) status and its non-toxic nature [209].

Challenges and Future Perspectives

Despite their advantages, several limitations hinder the widespread adoption of pullulan-based hydrogels. Their inherent mechanical weakness requires reinforcement for use in load-bearing or high-stress environments. Additionally, achieving consistent crosslinking and degradation profiles remains challenging, particularly when transitioning from lab-scale to industrial-scale production. Environmental sensitivity may affect long-term stability, and the relatively high production cost compared to synthetic polymers may limit commercialization. Nonetheless, ongoing research into advanced crosslinking strategies, composite formulations, and cost-effective manufacturing holds promise for overcoming these barriers and unlocking the full potential of pullulan hydrogels across biomedical and industrial domains [65,66,67,207,208,209].

## 3. Synthetic Hydrogels for Biomedical Applications

### 3.1. Overview

Synthetic hydrogels are 3D, hydrophilic polymeric networks capable of retaining large amounts of water while maintaining their structural integrity. Due to their tunable mechanical, chemical, and degradation properties, they have attracted substantial interest in biomedical applications such as DDS, wound healing, diagnostics, and TE. Unlike natural hydrogels, synthetic hydrogels offer precise control over stiffness, porosity, biodegradability, and bio-inertness, making them more suitable for engineered therapeutic systems. The inception of synthetic hydrogels dates back to 1960, when Wichterle and Lím introduced pHEMA, a hydrophilic polymer initially used for soft contact lenses due to its oxygen permeability and biocompatibility. This milestone catalyzed the rapid advancement of hydrogel research aimed at designing synthetic scaffolds mimicking biological environments [9,15,24,25,26,27,28,146].

Synthetic hydrogels offer distinct advantages over natural hydrogels for biomedical applications, primarily due to their high tunability, reproducibility, and structural precision. While natural hydrogels like alginate, collagen, or chitosan are biocompatible and derived from biological sources, synthetic hydrogels provide a level of control and customization that is crucial for advanced medical technologies. Key advantages of synthetic hydrogels in biomedicine include precise tunability, reproducibility and scalability, low immunogenicity, advanced drug delivery capabilities, stimuli-responsive behavior, support for 3D cell culture and bioprinting, and tissue-specific engineering. Mechanical strength, degradation rate, pore size, and swelling behavior are critical for matching tissue-specific requirements. These properties can be finely adjusted using synthetic polymers chemically modified with bioactive molecules, GFs, or cell adhesion peptides such as RGD sequences. Unlike natural materials, which can vary from batch to batch, synthetic hydrogels are highly consistent. This consistency is vital for clinical translation, FDA approval, and industrial production. Many synthetic polymers (e.g., PEG) are inert and non-immunogenic, minimizing immune response or inflammation, useful in DDS, implants, and injectable systems where immune evasion is key. Synthetic hydrogels can be designed for controlled and sustained release of small molecules, biologics (proteins, messenger ribonucleic acid (mRNA), antibodies), cells or exosomes, through diffusion, degradation-triggered or external stimulus-triggered mechanisms. Stimuli-responsive synthetic hydrogels can be engineered to respond to pH, temperature, light, enzymes, electric- or magnetic fields. PEG- or PVA-based hydrogels can be customized for 3D printing, organ-on-chip systems, or tumor models. They can mimic the ECM mechanically while remaining biochemically inert, allowing precise control of added biological signals. By blending different synthetic polymers or functionalizing them with biological cues, synthetic hydrogels can be tailored for neural tissue scaffolds, cardiac patches, cartilage regeneration, and wound healing matrices [146,210].

Table 3 highlights some quantitative data (Young’s modulus, tensile strength, swelling ratio, gelation time, in vitro degradation time, porosity), to compare the most important synthetic hydrogels for biomedical applications. Advantages, limitations, and key applications of synthetic hydrogels are exhibited in Table 4 [8,9,72,211].

Overall, synthetic hydrogels represent a cornerstone in the evolving landscape of biomedical technologies, bridging material science and RM to address complex healthcare needs.

Several synthetic polymers have since emerged, each with unique advantages for biomedical use. Key examples include poly(acrylamide) (PAAm), PEG, PVA, PAA, poloxamer, PNIPAAm, poly(lactic acid) (PLA)/PLGA, and polyurethane (PU).

### 3.2. Polyacrylamide-Based Hydrogels

#### 3.2.1. Structural Properties

PAAm is a linear synthetic polymer formed by the free radical polymerization of acrylamide monomers, yielding repeating –[CH_2_–CH(CONH_2_)]– units. In aqueous solution, PAAm chains can be crosslinked into 3D hydrogels through covalent bifunctional agents such as MBAm, divinylbenzene, or GAD, which bridge polymer chains at multiple points to create network junctions. Alternatively, ionic crosslinking with multivalent metal cations or physical gelation via hydrogen bonding and hydrophobic associations can also induce network formation. By controlling the type and concentration of crosslinker, the density of network junctions is tunable, allowing precise adjustment of gel porosity, mechanical strength, and swelling behavior [15,20,24,25,26,82,83,84,146].

#### 3.2.2. Fabrication Methods

The gelation of PAAm hydrogels proceeds primarily via free radical polymerization, initiated thermally (e.g., ammonium persulfate) or photochemically (UV activated initiators), generating macroradicals that propagate chain growth and simultaneously react with crosslinkers to form a stable network. Photopolymerization affords spatial control over gel geometry, enabling patterning and microfabrication. Ionic crosslinking leverages electrostatic interactions between anionic PAAm derivatives and oppositely charged ions, producing reversible gels with stimulus sensitive swelling. Thermosensitive PAAm derivatives, grafted with *N*-isopropylacrylamide (NIPAAm) or other responsive moieties, undergo sol–gel transitions with temperature shifts. Recent advances in click chemistry, such as azide–alkyne cycloaddition, permit rapid, bio-orthogonal crosslinking under mild conditions, broadening the repertoire of PAAm hydrogel architectures [15,20,24,25,26,146].

PAAm hydrogels are renowned for their exceptional hydrophilicity and high water content, often exceeding 90 wt %, which imparts soft, tissue-like mechanics and facilitates nutrient transport. Mechanical stiffness and elasticity are dictated by crosslink density and polymer molecular weight, enabling materials ranging from highly compliant (<10 kPa) to robust (>100 kPa) gels. Chemically inert under physiological conditions, PAAm exhibits excellent chemical stability and minimal protein adsorption, reducing nonspecific fouling. While native PAAm is non-degradable, copolymerization with hydrolyzable segments or incorporation of enzymatically cleavable linkers can endow controlled biodegradability. Moreover, functionalization with charged or bioactive groups allows tailoring of swelling kinetics, surface charge, and cell adhesive properties for specific biomedical needs [15,20,24,25,26,146].

#### 3.2.3. Biomedical Applications

In the biomedical arena, PAAm hydrogels serve myriad roles. As drug delivery matrices, their swelling controlled pore sizes and network meshes accommodate both small molecules and macromolecular therapeutics, enabling sustained, stimulus-responsive release profiles, e.g., pH or temperature triggered release of anticancer drugs like doxorubicin. In TE, PAAm gels mimic ECM properties, supporting cell encapsulation, proliferation, and differentiation in cartilage, bone, and soft tissue constructs. Their high moisture retention and conformability make them ideal for advanced wound dressings, which can be loaded with GFs or antimicrobials to accelerate healing. PAAm’s optical clarity and tunable porosity underpin its ubiquity in electrophoretic separation of proteins and nucleic acids, as well as in biosensor platforms for enzyme immobilization and diagnostic assays. Additionally, PAAm hydrogels are explored for contact lenses and cell delivery vehicles in RM [15,20,24,25,26,146,212,213,214,215,216].

#### 3.2.4. Limitations and Future Perspectives

Despite their versatility, PAAm hydrogels face challenges: intrinsic non-degradability raises concerns for long-term implants, and residual unreacted acrylamide monomers can pose cytotoxic risks if not rigorously purified. Excessive swelling may compromise mechanical integrity, while high crosslink densities can limit diffusion of nutrients and therapeutics. Thermal stability and gelation kinetics can vary with environmental conditions, complicating reproducibility. To address these issues, current research focuses on “smart” PAAm systems that respond to multiple stimuli (pH, light, biomolecules, etc.), biodegradable copolymers incorporating natural polymers (e.g., gelatin, chitosan), and nanocomposite hydrogels reinforced with SiO_2_ or metallic NPs for enhanced mechanics and multifunctionality. Advances in 3D bioprinting of PAAm based bioinks promise patient specific constructs with controlled architecture, moving toward personalized medicine and next-generation regenerative therapies [15,20,24,25,26,57,78,146,185,211,216,217,218,219,220,221].

### 3.3. Polyethylene Glycol-Based Hydrogels

#### 3.3.1. Structural Properties

PEG is a synthetic polyether of the general formula H–(O–CH_2_–CH_2_)_n_–OH, prized in biomedicine for its chemical inertness, hydrophilicity, and biocompatibility. Commercial PEGs range from oligomeric species (~200 Da) to high polymers (>100 kDa), with chain length directly governing solution viscosity, mesh size in hydrogels, and diffusion rates of solutes. By functionalizing the terminal –OH groups (e.g., with acrylates, vinyl sulfones, or thiols), PEG macromers can be crosslinked into 3D networks whose swelling behavior, pore architecture, and mechanical modulus are precisely tuned to application-specific requirements. PEG hydrogels exhibit high water content (often >90 wt %), conferring excellent nutrient diffusion and low protein adsorption, an asset for “stealth” implantable devices. Their mechanical properties span from very soft (kPa-range moduli mimicking brain tissue) to stiff (MPa-range gels for cartilage) by varying macromer concentration, chain length, and crosslink density [15,20,27,28,146,185].

#### 3.3.2. Fabrication Methods

PEG hydrogels are assembled via four principal methods. Physical gelation relies on reversible interactions, hydrogen bonding, hydrophobic association, or crystallite formation, yielding stimuli-responsive matrices that can sol–gel transition with temperature or pH shifts. Chemical crosslinking employs bifunctional reagents (e.g., GAD, diisocyanates) or “click” chemistries (thiol-ene Michael addition) to form stable covalent PEG–PEG linkages. Photo-crosslinking uses light activated initiators and acrylate/methacrylate moieties for rapid, spatially controlled network formation. Finally, enzymatic crosslinking (e.g., via TG or HRP) enables gelation under mild, cell friendly conditions, ideal for in situ TE. Incorporating thermoresponsive blocks (e.g., PNIPAAm segments) or pH sensitive moieties further endows PEG networks with triggerable swelling and deswelling, enabling on demand cargo release [15,20,27,28,82,83,84,146,185].

#### 3.3.3. Biomedical Applications

In the field of drug delivery, injectable PEG pre-gels that transition from sol to gel at body temperature enable localized and sustained release of small molecules and biologics. As a non-immunogenic polymer that is approved by the U.S. FDA, PEG is extensively utilized in DDS, particularly for formulations designed for sustained release. Drugs that are PEGylated, such as doxorubicin, benefit from being encapsulated in hydrogel depots, resulting in extended half-lives and diminished systemic toxicity. This strategy not only enhances the therapeutic effectiveness of these medications but also helps to mitigate adverse side effects, ultimately improving patient experiences and outcomes. In TE, photo-crosslinked PEGDA scaffolds support chondrocyte viability and cartilage matrix deposition, while in situ-forming PEG hydrogels deliver stem cells to bone or myocardial infarction sites with minimally invasive injections. For wound healing and sealant applications, PEG hydrogels create a moist, protective environment for wounds and can be incorporated with antimicrobial NPs for enhanced infection control. As surgical adhesives, such as PEG-based sealants, they offer strong tissue adhesion coupled with elastic compliance, ensuring effective sealing while allowing tissue movement. In the field of immunoengineering, PEG networks act as effective vaccine depots, optimizing the kinetics of antigen presentation. This controlled release mechanism significantly enhances both humoral and cellular immune responses, thereby improving the overall efficacy of vaccination strategies [15,20,27,28,33,57,146,185,212,213,214,215,216,217,218,219,220,221,222,223].

#### 3.3.4. Limitations and Future Perspectives

Despite their versatility, PEG hydrogels lack inherent bioactivity and must be functionalized with cell adhesive peptides (e.g., RGD) or GFs for effective tissue integration. Nondegradable PEG backbones can persist in vivo unless cleavable linkers (ester, disulfide, enzymatically labile) are incorporated. Cost of high purity PEG and rare immunogenic responses to PEGylated materials remain concerns. Emerging research focuses on “smart” PEG systems, incorporating multi-stimuli responsiveness, natural polymer hybrids, and nanocomposite reinforcements, to create hydrogels that seamlessly integrate tunable mechanics, targeted bioactivity, and controlled degradation for next-generation regenerative therapies and advanced DDS [15,20,27,28,33,146,185,221,222,223,224,225,226].

PEG hydrogels have been widely investigated for applications like drug delivery, injectable depots (for cancer therapy, orthopedics), cell encapsulation, TE scaffolds, and wound healing. Clinical trial outcomes of PEG hydrogels are represented by several FDA-approved formulations: e.g., OncoGel^®^ (Boston Scientific, Marlborough, MA, USA), VX-880^®^ (Vertex Pharmaceuticals, Boston, MA, USA), PEC-Encap^®^ (ViaCyte, San Diego, CA, USA), Durysta^®^ (Allergan, Dublin, Ireland), DuraSeal^®^ (Integra, Princeton, NJ, USA), ReSure^®^ Sealant (Ocular Therapeutix, Bedford, MA, USA), Coseal^®^ (Baxter Healthcare, Deerfield, IL, USA), and AdvaSeal^®^ (Ethicon, Raritan, NJ, USA) [58,227,228].

OncoGel^®^ utilizes a hydrogel matrix based on thermoresponsive triblock copolymer (PLGA–PEG–PLGA) specifically for paclitaxel delivery against glioma. This formulation is designed for local tumor management and is intended to release the drug at the tumor site. Phase I/II clinical trials showed sustained local drug release, reducing systemic toxicity. VX-880^®^ and PEC-Encap^®^ are both PEG-based cell encapsulation therapies for type 1 diabetes, but they differ in their approach to cell protection and delivery. VX-880^®^ involves implanting lab-grown islet cells under immunosuppression, while PEC-Encap^®^ encapsulates the cells in a device to protect them from immune rejection without the need for immunosuppressants. VX-880^®^ utilizes stem cell-derived islet cells, primarily containing beta cells, to restore insulin production. VX-880 is delivered intrahepatically under immune suppression. PEC-Encap^®^ is an encapsulated human pancreatic cell therapy, referred to as VC 01™. It uses a semi-permeable membrane to protect implanted cells from immune rejection. Its most significant challenge is that immune isolation was not fully effective—PEG membrane had fibrosis overgrowth, leading to reduced oxygen and nutrient flow [58,227,228].

Durysta^®^ is an FDA-approved, completed Phase III, biodegradable PEG hydrogel implant delivering bimatoprost for glaucoma. It effectively reduced intraocular pressure for up to 4–6 months, with no surgical removal required. Adverse events include mild to moderate, mostly eye irritation or corneal touch. ReSure^®^ Sealant, a PEG hydrogel that polymerizes in situ and reduces wound leakage, is recommended for ophthalmic incision sealing. DuraSeal^®^ is a PEG-based adhesive hydrogel that absorbs in ~4–8 weeks, for dural closure in neurosurgery. Coseal^®^, a PEG-based hydrogel crosslinked with thiol-reactive groups, is approved as a surgical sealant (vascular sealing). AdvaSeal^®^, a PEG-sodium hyaluronate hydrogel approved in Europe and under review in the U.S., is applied as anti-adhesion barrier for spinal surgery. PEGylated hydrogels for cartilage repair are used in combination with autologous chondrocytes. Clinical trials show improved integration, but mixed long-term success: some patients experienced mechanical failure due to inadequate stiffness matching with surrounding cartilage [58,227,228].

PEG hydrogels face a number of scalability challenges, such as sterility and crosslinking methods, batch variability, cost of Good Manufacturing Practice (GMP)-grade PEG macromers, storage and shelf-life, and biodegradability vs. stability. Many crosslinking chemistries (e.g., photopolymerization) cannot be scaled easily due to concerns with light penetration, cytotoxic photoinitiators (e.g., Irgacure 2959, BASF, Ludwigshafen, Germany), and oxygen inhibition. Enzymatic or click-chemistry crosslinking is promising but expensive and hard to scale. PEG is typically polydisperse unless custom-synthesized. Variations in molecular weight and end-group functionality lead to batch inconsistency in gel strength and degradation rate. PEGDA, PEG–maleimide, and other derivatives are expensive at GMP scale and difficult to purify from residual catalysts (e.g., tin, copper) or unreacted monomers. PEG hydrogels pre-crosslinked tend to dry or degrade. Crosslink-on-demand systems increase complexity in delivery systems (e.g., dual syringe devices, UV lamps). Balancing degradation rate for therapeutic efficacy vs. long-term structural integrity remains tricky, especially in orthopedics or neurosurgery applications [58,227,228].

### 3.4. Poly(vinyl Alcohol)-Based Hydrogels

#### 3.4.1. Structural Properties

PVA is a synthetic, water-soluble polymer obtained by hydrolyzing poly(vinyl acetate), yielding a linear backbone of –[CH_2_–CH(OH)–]_n_– units [15,17,20,146,212,213,214,215]. The degree of hydrolysis (i.e., fraction of acetate groups removed) and polymer molecular weight (typically 10–100 kDa) govern PVA’s crystallinity, solubility, and mechanical strength. PVA hydrogels can be fabricated without added reagents via repeated freeze–thaw cycles, which induce microcrystalline junctions, or by γ irradiation that generates radical mediated crosslinks. Alternatively, chemical crosslinkers—such as GAD, boric acid, or “click” reagents—can form stable covalent bonds, allowing precise control over network architecture and stiffness. PVA hydrogels exhibit high water uptake (>90 wt %), yielding large swelling ratios that facilitate nutrient diffusion and sustained release of payloads. Mechanical properties, from soft (kPa scale) to robust (MPa scale), are tuned by altering polymer concentration, molecular weight, and crosslink density. Thermal stability up to ~200 °C in dry form and resistance to aqueous hydrolysis make PVA suitable for sterilizable devices. Biocompatibility is generally excellent, though influenced by residual acetate content and choice of crosslinkers. Their low protein adsorption further minimizes foreign body responses in vivo [15,17,20].

#### 3.4.2. Fabrication Methods

PVA networks assemble through multiple mechanisms. Physical crosslinking arises from extensive hydrogen bonding and hydrophobic microdomain formation, often yielding TRHs. Chemical crosslinking uses bifunctional agents (e.g., GAD, ECH) to react with –OH groups, creating permanent covalent bridges. Freeze–thaw cycling relies solely on crystallite formation to lock chains into a hydrogel matrix, an entirely additive free method prized for its cytocompatibility. Gamma irradiation induces direct covalent crosslinks via backbone radical generation, enabling sterile hydrogel formation without chemical residues [15,17,20,82,83,84].

#### 3.4.3. Biomedical Applications

In DDS, PVA matrices provide controlled, localized release of small molecules (e.g., ibuprofen, doxorubicin) and proteins, with thermo- or pH-responsive blends enabling on demand release. TE employs PVA–alginate and PVA–collagen composites as mechanically supportive, cell permissive scaffolds for cartilage and skin regeneration; injectable, freeze–thawed PVA offers minimally invasive scaffold implantation. Wound dressings leverage PVA’s moist environment and can incorporate antimicrobial NPs (SiO_2_, Ag) to accelerate healing and prevent infection. In ophthalmology, PVA-based hydrogels serve as artificial tears and hydrophilic contact lenses, enhancing ocular comfort. Diagnostics benefit from PVA films as enzyme immobilization platforms in biosensors, capitalizing on their transparency and stability [6,15,17,20,229,230,231,232].

#### 3.4.4. Limitations and Future Perspectives

Pure PVA lacks intrinsic cell adhesive or bioactive motifs, necessitating conjugation of peptides (e.g., RGD) or blending with natural biopolymers to enhance cellular interactions. Its slow biodegradation in physiological environments can lead to long-term persistence unless degradable linkers (ester, disulfide) are introduced. Excessive swelling may compromise mechanical integrity or trigger burst release of encapsulated agents. Future research is focusing on “smart” PVA hydrogels, incorporating multi-stimuli responsiveness (pH, light, biomolecules), nanocomposite reinforcement with carbon or SiO_2_ for improved mechanics, and 3D bioprintable formulations for patient specific tissue constructs. Through these advances, PVA hydrogels will continue to evolve as versatile platforms in next-generation DDS, RM, and BME [6,15,20,57,146,211,212,213,214,215,216,217,218,219,220,226,229,230,231,232,233,234].

PVA hydrogels have been investigated for drug delivery, artificial cartilage (e.g., knee meniscus, intervertebral disc), contact lenses, embolic agents, and wound dressing. Clinical trial outcomes of PVA hydrogels include some FDA-approved formulations, such as Durasoft^®^ Contact Lenses (Alcon, Geneva, Switzerland), NUsurface^®^ Meniscus Implant (Active Implants LLC, Memphis, TN, USA), and Contour^®^ Embolization Particles (Boston Scientific, Marlborough, MA, USA). Due to water content and flexibility, PVA derivatives are widely used as hydrogels for daily disposable contact lenses for vision correction, e.g., Durasoft^®^ (Nelfilcon A). NUsurface^®^ Meniscus Implant (NUsurface, Memphis, TN, USA), made from PVA hydrogel, is the subject of several multicenter clinical trials in the U.S. (advanced clinical stages) and Europe (e.g., VENUS or SUN studies). It was designed as a partial meniscus replacement for patients with medial compartment knee pain, demonstrating reduced pain and improved function, acting as a “bridge” before knee replacement, not intended to be permanent. Challenges of cartilage replacement include long-term fixation, integration, and occasional device migration. Contour^®^ Embolization Particles, a widely used product for embolization in uterine fibroids and tumors, is not in hydrogel form per se, but crosslinked PVA microparticles. Studies on PVA-based wound dressings (e.g., hydrocolloid into PVA matrix) show good moisture retention and healing rates, but no large-scale clinical trials for hydrogel-only PVA dressings have gone to FDA approval [58,212,214].

### 3.5. Poly(acrylic Acid)-Based Hydrogels

#### 3.5.1. Structural Properties

PAA is a water-soluble, anionic polymer obtained by free radical polymerization of acrylic acid monomers. Its backbone comprises repeating –[CH_2_–C(=O)–C(OH)–]_n_– units, each bearing a –COOH moiety whose ionization state varies with pH. In alkaline environments, deprotonated carboxylates impart strong electrostatic repulsion and chain expansion, whereas in acidic media, protonated groups favor coil formation. Crosslinking, either covalent (e.g., MBAm) or ionic (via multivalent cations like Ca^2+^), converts soluble PAA into 3D hydrogels capable of dramatic, reversible swelling. PAA hydrogels stand out for their pronounced pH sensitivity: swelling ratios can exceed 100× in basic media yet collapse under acidic conditions. This tunable expansion is ideal for on demand release of encapsulated agents. Mechanical strength and elasticity are governed by crosslink density: increasing MBAm or ionic crosslinker content produces stiffer gels, while low crosslinking yields softer, highly deformable matrices. The abundant –COOH groups confer high water uptake and excellent biocompatibility, although unmodified PAA may require purification or copolymerization to attenuate any cytotoxicity associated with residual monomers [15,20,82,83,84].

#### 3.5.2. Fabrication Methods

PAA hydrogels are synthesized through several complementary strategies. Free radical polymerization, initiated thermally or by redox systems, yields high-molecular weight networks rich in hydrogen-bonding sites. Ionic crosslinking occurs when divalent or trivalent metal ions bridge adjacent carboxylates, reinforcing the gel matrix and enhancing mechanical performance. Physical crosslinking, driven by hydrogen bonding and hydrophobic interactions, can be exploited under controlled pH or temperature to form reversible networks. Finally, composite formulations, in which PAA is copolymerized or blended with polysaccharides (alginate, gelatin) or NPs, offer hybrid gels with tailored stiffness, bioactivity, and stimulus responsiveness [15,20].

#### 3.5.3. Biomedical Applications

PAA materials exhibit versatile applications across several fields, including DDS, TE, wound healing, diagnostics, and ophthalmic devices. In DDS, pH-responsive PAA gels enable targeted drug release in GI or tumor microenvironments, with doxorubicin-loaded PAA NPs demonstrating sustained, acid-triggered release for enhanced therapeutic efficacy. In TE, PAA–alginate scaffolds, formed through ionic crosslinking, promote cell adhesion and proliferation, while conductive PAA–graphene composites facilitate neural tissue regeneration by transmitting electrical signals. For wound healing, PAA hydrogels maintain a hydrated environment that is vital for recovery; when loaded with antimicrobial agents like tannin or Ag, they exhibit antimicrobial properties and accelerate re-epithelialization. In diagnostics, PAA hydrogels serve as matrices for enzyme immobilization or molecular imprinting, leading to the development of sensitive biosensors for detecting glucose, pathogens, and biomarkers. Lastly, in ophthalmic applications, PAA-based lens coatings and tear substitute gels leverage their high hydration capacity to improve contact lens comfort and effectively address dry eye syndrome [15,20,146,212,213,214,215,216,217,218].

#### 3.5.4. Limitations and Future Perspectives

Despite their versatility, PAA hydrogels suffer from limited inherent bioactivity, potential accumulation due to slow biodegradation, and sensitivity to ionic strength that can unpredictably alter swelling and release profiles. Excessive swelling may compromise mechanical integrity or cause burst release of payloads. To overcome these challenges, current research focuses on chemical functionalization with cell adhesive peptides or degradable linkers, “smart” stimuli-responsive networks (pH, temperature, light), and multicomponent composites that synergize PAA’s responsiveness with the strength or bioactivity of natural polymers and nanomaterials. Emphasis on green synthesis, using enzymatic or radiation mediated crosslinking, promises environmentally benign, cell friendly production routes for next-generation PAA hydrogels [6,15,20,69,217,218,229,230,231,232,233,234,235].

### 3.6. Poloxamer-Based Hydrogels

#### 3.6.1. Structural Properties

Poloxamers are synthetic triblock copolymers with a peculiar structure: two blocks of poly(ethylene oxide) (PEO) flanking a central block of poly(propylene oxide) (PPO), HO–[CH_2_–CH_2_–O–]*_x_*–[CH_2_–CH(CH_3_)–O–]*_y_*–[CH_2_–CH_2_–O–]*_x_*–H. Commercially known as Pluronic^®^ (BASF, Ludwigshafen, Germany), Synperonic^®^ (Croda, Snaith, UK) or Lutrol^®^ (BASF, Ludwigshafen, Germany), poloxamers can have different states of aggregation (liquid, paste, solid), and also hydrophobic or hydrophilic behavior, depending on molecular weight (1100 to 14,000) and ethylene oxide (EO)/propylene oxide (PO) weight ratios (1:9 to 8:2), respectively. Poloxamer 188 (Pluronic F68), poloxamer 237 (Pluronic 87), poloxamer 338 (Pluronic 108), and poloxamer 407 (Pluronic 127) are commonly used and freely soluble in water. Poloxamer hydrogels are thermosensitive, ensuring transition from a liquid state at room temperature to a gel state through exposure to body temperature. Hydrophobic PPO blocks in poloxamers interact when heated, with the formation of micelles. At a 20% concentration in water, the widely used poloxamer 407 can form a clear gel around 37 °C [236].

#### 3.6.2. Fabrication Methods

Poloxamers are synthesized by sequential addition of hydrophilic EO and hydrophobic PO monomers, in alkaline medium (e.g., sodium hydroxide, potassium hydroxide). Macromolecular surface-active agents with specific properties are obtained by modifying copolymer composition (PPO/PEO ratio) and molecular weight (PEO/PPO block length) during synthesis [236].

Poloxamer hydrogels are fabricated using different methods, such as hot process, crosslinking. and photopolymerization. In the hot process, solid poloxamer is mixed with water, heated (at 80 °C) to obtain a solution, then cooled to room temperature for the formation of a hydrogel. A 3D hydrogel network can be obtained through modification of poloxamers with reactive groups (e.g., acrylate) and crosslinking via photopolymerization, after adding a photoinitiator and exposure to UV light [236,237].

#### 3.6.3. Biomedical Applications

Poloxamers are generally considered biocompatible, non-toxic, and suitable for biomedical applications. Poloxamer TRHs are used for controlled drug release, TE, and as food additives. The encapsulated drug is slowly released from hydrogel upon warming, extending the duration of action and reducing side effects. Ophthalmic, injectable, transdermal, and vaginal drug delivery benefits from the special properties of poloxamer hydrogels [236,237]. Most applications involve the use of poloxamer 407 and include controlled delivery of protein/peptide drugs [238], such as insulin [239], interleukins [240], epidermal growth factor [241], bone morphogenetic protein [242], fibroblastic growth factor [243], mesenchymal stem cells (MSCs) [244], and VEGF [98]. Based on their biocompatibility, low cytotoxicity, good rheological properties, and ability to create a scaffold-like structure for cell growth and regeneration, poloxamers have received special attention for TE applications. Poloxamer thermoreversible hydrogels are easily extruded from 3D printers due to their gelation characteristics, as they quickly pass from liquid to gel state. Poloxamer 407 showed good printability, but it is not suitable for long-term cell viability. However, exploiting its elasticity and excellent rheological behavior, under shear stress, Pluronic 127 was combined with gelatin to create a biocompatible hydrogel for vascular channels [236,237].

#### 3.6.4. Limitations and Future Perspectives

Their thermoresponsive behavior, biocompatibility, safe degradation in the body, and customizability make poloxamer hydrogels ideal for next-generation DDS and TE applications, especially for the advancement in bioprinting technology. While the future of poloxamer hydrogels is promising, some challenges remain to be addressed: cytotoxicity at high concentrations, limited mechanical strength, scalability and sterilization in commercial production. In fact, one of the major disadvantages of poloxamer hydrogels is that they do not facilitate differentiation of cells into multiple linkages. Poloxamer hydrogels are expected to play a major role in controlled and localized drug delivery, combined with NPs or liposomes for precision targeting, particularly for (*i*) cancer therapy, to reduce systemic toxicity of chemotherapeutics, (*ii*) ophthalmic drugs used for chronic eye conditions, and (*iii*) intranasal and oral delivery for faster mucosal absorption and brain targeting (e.g., in Alzheimer’s disease and Parkinson’s disease). Future perspectives in RM and TE refer to the use of poloxamer hydrogels as growing tissue or cells scaffolds, mainly for neural TE, cartilage regeneration, and wound healing. The potential for minimally invasive therapies, thermoresponsive “smart” systems, 3D bioprinting applications, and injectable hydrogels with stem cells, GFs, analgesic and anti-inflammatory drugs must also be considered. Also, an important direction for patient-specific treatments is represented by hybrid systems fabricated by blending poloxamers with biopolymers like chitosan, HA, or alginate to improve biocompatibility, adhesion, and mechanical strength [241,245,246].

### 3.7. Poly(N-Isopropylacrylamide)-Based Hydrogels

#### 3.7.1. Structural Properties

PNIPAAm is a synthetic polymer obtained by free radical polymerization of NIPAAm monomers, typically initiated using azobisisobutyronitrile (AIBN) or UV activated photoinitiators. Its hallmark property is a sharp, reversible coil–globule transition at the lower critical solution temperature (LCST ≈32 °C), where intra- and intermolecular hydrogen bonds with water break down, rendering the polymer hydrophobic and collapsed. Below the LCST, PNIPAAm chains are fully hydrated and swollen; above it, they expel water and undergo a dramatic volumetric shrinkage. Copolymerization with hydrophilic or hydrophobic comonomers allows precise tuning of the LCST over a range of 20–40 °C, tailoring PNIPAAm’s responsiveness for specific applications. PNIPAAm hydrogels combine high water content with tunable mechanical and swelling characteristics. Swelling ratios can exceed 50× their dry volume below the LCST and rapidly collapse above it, enabling pulsatile release profiles. Mechanical properties span from soft (tens of kPa) to moderately stiff (hundreds of kPa) depending on crosslink density and polymer concentration. The hydrophilic–hydrophobic transition imparts dynamic changes in permeability, solute diffusion, and surface wettability. Moreover, PNIPAAm networks can be engineered to display hysteresis in their phase transition or dual responsiveness to pH and temperature by incorporating ionizable comonomers [15,18,20].

#### 3.7.2. Fabrication Methods

PNIPAAm hydrogels are fabricated via several crosslinking strategies. Chemical crosslinking with MBAm or divinyl monomers yields permanent covalent networks that maintain LCST responsiveness. Physical gelation can be achieved by blending PNIPAAm with triblock copolymers (e.g., Pluronic^®^) or by exploiting hydrophobic association at elevated temperatures to form reversible junctions. Radiation induced crosslinking (γ or electron beam) offers residue free network formation, while composite approaches incorporate NPs (Au, SiO_2_) or natural polymers (PEG, gelatin) to reinforce mechanical integrity and introduce multifunctionality such as photothermal conversion or enhanced bioactivity [15,18,20].

#### 3.7.3. Biomedical Applications

The LCST driven volume change of PNIPAAm hydrogels underpins numerous biomedical innovations. In drug delivery, temperature triggered collapse above body temperature can expel encapsulated therapeutics (e.g., proteins, anticancer drugs) in localized hyperthermia treatments. TE leverages PNIPAAm’s cell sheet detachment upon cooling, enabling non-enzyme mediated harvest of confluent cell layers for regenerative therapies. Biosensing exploits the polymer’s volumetric response to transduce biochemical signals, such as glucose or urea concentrations, into measurable optical or mechanical outputs. Wound dressings and surgical adhesives tap PNIPAAm’s moisture retention and thermoadhesive properties, while “smart” wearables incorporate PNIPAAm into textiles for adaptive thermal regulation [6,15,18,20,146,212,213,214,215,216,217,229,230,231,232,233,234].

#### 3.7.4. Limitations and Future Perspectives

Despite its versatility, PNIPAAm faces challenges including limited long-term biocompatibility due to hydrophobic collapse promoting protein fouling, insufficient mechanical strength for load-bearing applications, and non-degradability in vivo. LCST can shift unpredictably in complex media with salts or proteins, reducing reproducibility. Future research is directed toward biodegradable PNIPAAm analogs incorporating cleavable linkers, multi-stimuli-responsive copolymers (pH, light, magnetic field), and nanocomposite reinforcement to boost mechanics and introduce imaging or therapeutic functionalities. Advances in 3D bioprinting of PNIPAAm-based bioinks and precision copolymer design promise to expand its role in next-generation responsive biomaterials [69,146,185,212,213,214,215,216,217,229,230,231,232,233,234].

### 3.8. Poly(lactic Acid)/Poly(lactic-Co-Glycolic Acid)-Based Hydrogels

#### 3.8.1. Structural Properties

The hydrophilicity and water uptake of PLA/PLGA hydrogels can be effectively tuned by adjusting the LA-to-glycolic acid (GA) ratio. Higher GA content enhances the hydrophilicity of the hydrogels, resulting in increased water absorption and accelerated degradation rates. Additionally, the crosslinking density within the hydrogel network plays a crucial role in determining its elasticity and tensile strength, allowing for further optimization of mechanical properties to meet specific application requirements. By strategically manipulating both the LA-to-GA ratio and crosslinking density, hydrogels can be engineered for improved performance in various biomedical applications. Degradation proceeds via bulk hydrolysis of ester bonds, generating LA and GA byproducts that enter natural metabolic pathways, though localized pH drops can occur in poorly buffered environments. Mechanical properties span from soft (tens of kPa) to robust (MPa) depending on polymer weight, crosslinker choice, and processing method. Porosity and mesh size can be precisely engineered (e.g., by particulate leaching or electrospun fiber diameters) to support cell infiltration, nutrient diffusion, and controlled release of encapsulated agents [11,15,18,20].

#### 3.8.2. Fabrication Methods

PLA and its copolymer with GA, PLGA, are aliphatic polyesters synthesized by ring opening polymerization of lactide and glycolide cyclic monomers, respectively. The precise ratio of LA to GA units in PLGA governs its hydrophobicity, crystallinity, mechanical strength, and hydrolytic degradation rate, enabling tailored materials ranging from stiff, slowly resorbing scaffolds to more flexible, rapid degrading matrices. PLA and PLGA hydrogels are formed by creating 3D networks through physical gelation (e.g., hydrogen bonding or hydrophobic interactions in aqueous media), chemical crosslinking (using diisocyanates, carbodiimides, or multifunctional acrylates), solvent casting with particulate leaching, or electrospinning to yield fibrous mats. These techniques produce tunable pore structures, swelling behavior, and mechanical properties suited to specific biomedical uses [6,69,146,212,213,214,215,216,217,218,229,230,231,232,233,234,235].

#### 3.8.3. Biomedical Applications

PLA and PLGA hydrogels excel as drug delivery vehicles by encapsulating both hydrophilic and hydrophobic therapeutics and offering sustained, controlled release pro-files. PLGA–PNIPAAm thermoresponsive blends exploit temperature induced sol–gel transitions for minimally invasive injection and subsequent depot formation. Embedding PLGA microspheres within a hydrogel matrix enables sequential or programmed release of multiple payloads, proteins, peptides, or small molecules, by decoupling matrix swelling from microsphere degradation. Composite hydrogels incorporating chitosan or alginate further improve drug loading efficiency, mucoadhesion, and release kinetics, making these systems ideal for localized cancer therapies, anti-inflammatory treatments, and long-acting injectable formulations. In TE, PLA/PLGA hydrogels serve as scaffolds for bone, cartilage, and soft tissue regeneration, with composite variants (e.g., PLGA–bioactive glass or calcium phosphate) enhancing osteoconductivity and mechanical competence. Electrospun PLA/PLGA fiber mats mimic ECM architecture, promoting cell adhesion, proliferation, and differentiation. For wound care, hydrogels maintain a moist environment, can be loaded with antimicrobial agents (AgNPs, antibiotics), and facilitate controlled GFs release. Diagnostic applications leverage hydrogel matrices to immobilize enzymes or imaging agents, enabling real time monitoring of drug release, pH changes, or marker detection via embedded fluorescent or magnetic NPs [6,11,15,78,80,146,212,213,214,215,216,217,218,219,229,230].

#### 3.8.4. Limitations and Future Perspectives

The unpredictable swelling behavior of these hydrogels can adversely affect drug release kinetics and mechanical stability in vivo, as observed in hydrogels used for vaginal or rectal delivery systems [9,21]. Mechanical strength is another concern, as many PLA/PLGA hydrogels may exhibit insufficient load-bearing capacity. Predicting release kinetics is also complicated by the interplay between hydrogel swelling and polymer degradation dynamics [15,20,185].

Research is focusing on the development of “smart” hydrogels that can respond to multiple stimuli, such as pH, temperature, and electric field. The incorporation of biodegradable linkers aims to fine-tune erosion rates, while integrating nanomaterials (like graphene and SiO_2_) seeks to enhance mechanical reinforcement and introduce multifunctionality. Furthermore, advancements in 3D bioprinting technologies utilizing PLA/PLGA bioinks are paving the way for patient-specific scaffolds with precise architectural designs. Regulatory and manufacturing improvements are anticipated to accelerate the translation of these innovative hydrogels into next-generation solutions for DDS and RM [6,15,20,146,185,212,213,214,215,216,217,218,229,230,231,232,233,234,235].

### 3.9. Polyurethane-Based Hydrogels

#### 3.9.1. Structural Properties

PUs are segmented copolymers featuring alternating soft (polyol derived, hydrophilic) and hard (di- or poly-isocyanate derived, hydrophobic) domains linked by urethane (–NH–COO–) bonds. By selecting the type and molecular weight of the polyol, the functionality (di-, tri-, or higher) of the isocyanate, and the soft/hard segment ratio, PU networks can be finely tuned in terms of hydrophilicity, elasticity, mechanical strength, and degradation behavior. In hydrogel form, PUs assemble into 3D, water-swellable networks whose elastic, tear-resistant character arises from the reversible association of soft segments interspersed with rigid, hydrogen bonding hard segments. PU hydrogels swell to high water contents (often >80 wt %), yet maintain resilience and elasticity even under large deformations. Their mechanical modulus can span from tens of kPa (soft tissue mimics) to MPa (load-bearing constructs) by adjusting crosslink density and segment composition. The interplay between hydrophilic soft segments and hydrophobic, hydrogen-bonding hard segments confers rapid shape recovery, tunable permeability, and minimal creep. Many PU formulations incorporate biodegradable ester or carbonate linkages in the soft blocks to permit hydrolytic erosion and control mass loss [6,15,20,33,35].

#### 3.9.2. Fabrication Methods

PU hydrogels are typically prepared by (*i*) prepolymer methods, where an isocyanate-terminated prepolymer is chain extended or crosslinked with polyols or diamines to create a network; (*ii*) click chemistry approaches, for example, azide–alkyne cycloadditions, that enable rapid, quantitative crosslinking under mild conditions and precise control over network architecture; and (*iii*) in situ polymerization, wherein reactive PU precursors are mixed and gelled directly at the application site (e.g., via injectable formulations), allowing hydrogels to form under physiological conditions [6,15,20,33,35].

#### 3.9.3. Biomedical Applications

In wound healing, PU hydrogels maintain a moist environment, permit oxygen ex-change, and can be loaded with antimicrobials to reduce infection. As drug delivery matrices, their swelling controlled release profiles and enzymatically or hydrolytically degradable linkers enable sustained, localized delivery of small molecules, peptides, or NPs. TE scaffolds benefit from the tailorability of stiffness and degradation rate, supporting cell adhesion, proliferation, and differentiation in cartilage, bone, and neural regeneration. Implantable coatings on stents or catheters exploit PU hydrogels’ antifouling and thromboresistant properties. Finally, biosensor platforms integrate conductive fillers (e.g., CNTs, graphene) into PU matrices to enable real time biomarker detection and bioelectronic interfacing [78,80,146,212,213,214,215,216,217,218,229,230,231,232,233,234,235].

#### 3.9.4. Limitations and Future Perspectives

Mechanical properties may also vary with humidity and temperature, complicating their use in dynamic in vivo environments. “Smart” PU hydrogels that can respond to stimuli such as pH, temperature, and light for on-demand drug release applications are under development. Furthermore, strategies involving nanocomposite reinforcement are being employed to improve the mechanical properties of these hydrogels while also incorporating functionalities such as antimicrobial activity and advanced imaging capabilities. This integrative approach aims to create more effective and versatile hydrogel systems for a wide range of biomedical applications. Advances in green synthesis, such as biobased polyols and catalyst free click reactions, promise more sustainable, clinically translatable PU hydrogel platforms [57,146,211,212,213,214,215,216,217,218,219,220,221,229,230,231,232,233,234].

## 4. Hybrid Hydrogels for Biomedical Applications

### 4.1. Overview

Hybrid hydrogels represent an advanced class of materials synthesized by combining natural and synthetic polymers to create multifunctional systems with superior biocompatibility and mechanical strength. Natural polymers such as chitosan, alginate, and collagen impart biological recognition, degradability, and low immunogenicity, whereas synthetic polymers like PVA, PEG, and PAAm offer tunable mechanical and physicochemical properties. The integration of these polymers within a single hydrogel matrix leads to a 3D, hydrophilic polymer network that exhibits desirable features from both components. These materials are tailored for biomedical applications, including wound healing, controlled drug delivery, biosensing, and RM, due to their customizable structure and multifunctionality [16,20,29].

Hybrid hydrogels are one of the most promising material innovations in the biomedical field. They combine the best features of hydrogels with nanomaterials, fibers, or other polymers to overcome the limitations of conventional single-component hydrogels. Hybrid hydrogels exhibit key advantages such as enhanced bioactivity, improved mechanical strength, multifunctionality, antibacterial or antioxidant properties, improved drug delivery, tunable degradation rates, and customizability for 3D bioprinting. Base synthetic hydrogels (e.g., PEG, PVA) are bioinert. Adding bioactive fillers like gelatin, collagen, HA, GFs enables cell adhesion, proliferation, and signaling for stem cell delivery, skin grafts, or wound healing. Most pure hydrogels are weak and brittle, especially under load. Adding nanofibers, clay NPs, or graphene increases tensile and compressive strength, essential for applications like cartilage repair, bone scaffolds, and load-bearing tissues: e.g., Laponite (clay NP)-reinforced hydrogels used in cartilage repair showed two to five times increased Young’s modulus. Hybrid hydrogels can be designed to be stimuli-responsive such as thermoresponsive, pH-responsive, or electroconductive for neural or cardiac tissue scaffolds: e.g., polypyrrole (PPy) or GO composites used for nerve regeneration or cardiac patches. Adding AgNPs, zinc oxide, or curcumin-loaded NPs to hydrogels creates self-sterilizing chronic wound dressings, burns, and surgical implants. Incorporating microparticles, liposomes, or nanogels into hydrogels allows dual release profiles, such as fast initial release from hydrogel or sustained release from embedded NPs, useful for chemotherapy, wound healing agents, insulin, or vaccine delivery. Composite systems can incorporate slow-degrading inorganic NPs (e.g., hydroxyapatite) or enzymatically degradable fibers, allowing better control over how fast the TE scaffolds break down, depending on the tissue. Hybrid hydrogels allow better 3D printability, structural fidelity, and cell encapsulation for biofabricated organs, skin grafts, and in vitro tissue models: e.g., adding cellulose nanofibers or silk can improve viscosity and 3D print resolution [247,248].

Nanocomposite hydrogels are transforming the biomedical materials field by integrating nanomaterials, such as graphene/GO, AgNPs, CNTs, SiO_2_ NPs, magnetic (Fe_3_O_4_) NPs, and nanoclays (e.g., Laponite), typically <100 nm, into hydrogel matrices. They aim to combine the hydrophilic, soft, and biocompatible nature of hydrogels with the unique properties of nanomaterials. Hydrogels are often weak and tear prone. Nanofillers address the impact on mechanical properties by enhancing tensile and compressive strength, improved self-healing, shear-thinning and printability. NPs interact physically or chemically with the polymer matrix, acting as physical crosslinking points, resulting in greater Young’s modulus, toughness, and elastic recovery: e.g., GO/PVA nanocomposite hydrogels showed five times tensile strength increase over PVA alone. Some nanocomposites, mainly with GO or boronate complexes, allow dynamic bonding, enabling self-repair after damage. Also, NPs can make hydrogel viscoelastic, which is ideal for 3D bioprinting [248,249,250,251].

Nanomaterials, such as graphene/GO, AgNPs, functionalized CNTs, SiO_2_ NPs, and Fe_3_O_4_ NPs, add biological function to inert hydrogels. Graphene/GO nanocomposite hydrogels promote cell adhesion, proliferation, and neural differentiation, also having antibacterial and anti-inflammatory properties. Their conductivity enables applications in neural, cardiac, and muscle TE: e.g., GO/gelatin hydrogel used for neural stem cell scaffolds promotes neurite outgrowth. AgNPs nanocomposite hydrogel is a potent broad-spectrum antimicrobial applied for wound dressings, surgical coatings, and burn treatments: e.g., AgNPs/chitosan hydrogels show >95% bacterial kill rate and accelerate wound closure in vivo. Unlike CNTs, which are cytotoxic, functionalized CNTs with high electrical conductivity and mechanical strength are safer and support electrically active tissue regeneration, e.g., promoting neuronal differentiation and axon alignment. Fe_3_O_4_ NPs enable magnetically controlled movement, remote hyperthermia, or stimulus-responsive drug release. Also, they are used in MRI contrast and targeted therapy: e.g., magnetic nanocomposite hydrogels used in glioblastoma therapy for combined chemo-magnetic hyperthermia [248,249,250,251].

Challenges of nanocomposite hydrogels refer to cytotoxicity, NP aggregation, their cost, environmental persistence, and regulatory barriers. Some nanomaterials (e.g., CNTs, AgNPs at high doses) can damage cells and NP aggregation can reduce uniformity and function of hydrogels. Also, high-purity nanomaterials are expensive at scale. From the point of view of their environmental safety, NPs may accumulate or persist in ecosystems. Therefore, FDA approval is harder due to NPs safety uncertainties. Over the next 5–10 years, more FDA-approved hybrid devices are expected, including integration with wearable/implantable biosensors, tailored microenvironments for organoid growth and cell therapy, and smart drug carriers that adapt to the body’s signals [248,249,250,251].

In order to compare the most important hybrid hydrogels for biomedical applications, some quantitative data (Young’s modulus, tensile strength, swelling ratio, gelation time at 37 °C, in vitro degradation time, and porosity) are shown in Table 5. In addition, for an overall reference, Table 6 highlights the advantages, limitations, and key applications of hybrid hydrogels [16,72,210,211,252].

### 4.2. Fabrication Methods

Various synthesis methods have been developed to fabricate hybrid hydrogels with controlled architecture and properties. Physical blending is the most straightforward approach, involving the simple mixing of polymers in aqueous solutions followed by physical or chemical crosslinking to form stable hydrogel matrices. Chemical crosslinking employs agents like GAD or carbodiimide to create covalent bonds between polymer chains, enhancing structural integrity and enabling modulation of properties such as elasticity and degradation rate. In situ polymerization offers site-specific gelation within biological environments by triggering polymerization using stimuli like light or heat, thus allowing the hydrogel to conform to irregular tissue architectures and function as a biomimetic scaffold. Recent developments in 3D printing and biofabrication further allow the construction of hybrid hydrogel structures with precise geometry and spatial distribution, facilitating applications in TE and personalized medicine [16,20,29,82,83,84].

### 4.3. Biomedical Applications

Hybrid hydrogels have shown significant promise as scaffolds in TE due to their biomimetic and structural properties. For skin and cartilage regeneration, hydrogels composed of collagen and chitosan promote cell adhesion, proliferation, and tissue remodeling, mimicking the ECM and enhancing dermal and epidermal healing [30,31,43,76,81,90,105,111,155]. In cartilage repair, hybrid systems such as GelMA with chondroitin sulfate or collagen–PEG composites replicate the viscoelastic properties of native cartilage while supporting chondrocyte viability and ECM production [16,20,29]. These hydrogels also serve as delivery platforms for MSCs and GFs to improve cartilage regeneration outcomes [16,20,29]. In dermal applications, HA–PU hybrids offer tunable viscoelasticity and hydration, showing great potential for injectable fillers and wound repair [16,20,29,32,170,253].

Hybrid hydrogels offer precise control over drug release profiles, a critical requirement for effective therapeutic interventions. Their swelling behavior, degradation kinetics, and polymer interactions can be engineered to modulate the encapsulation and sustained release of drugs or biomolecules [16,20,29]. For example, alginate–PAAm hydrogels combine biocompatibility with adjustable mechanical properties, making them suitable for DDS and cell culture platforms [175]. Similarly, gelatin–PEG hydrogels are extensively explored for their biodegradability and tailored drug release characteristics, supporting applications in RM and controlled pharmacotherapy [6,16,17,20,27,29]. Furthermore, hybrid hydrogels are increasingly utilized in drug-eluting stents, allowing localized delivery of anti-proliferative agents to mitigate restenosis after vascular interventions.

In bone regeneration, hybrid hydrogels offer an ideal balance of bioactivity and mechanical reinforcement. SF–PEG hydrogels enhance hydrophilicity and mechanical performance, creating conducive environments for osteoblast activity and new bone formation [16,17,20,27,29,107]. The incorporation of bioactive ceramics such as hydroxyapatite into the hydrogel matrix further supports osteoconduction and stimulates cellular responses required for bone regeneration [254]. These materials provide both the mechanical framework and biochemical cues needed for the recruitment and differentiation of progenitor cells. Future directions involve refining the composition and processing techniques to optimize scaffold performance, degradation behavior, and clinical translation in bone repair applications [254].

Hybrid hydrogels also play a pivotal role in the development of biosensors for diagnostics and continuous monitoring. By embedding enzymes, antibodies, or nucleic acids into the hydrogel matrix, these systems can detect biological molecules with high specificity and sensitivity. For example, PEG–chitosan hydrogels loaded with glucose oxidase (GOx) enable glucose monitoring through enzymatic reactions that generate electrochemically detectable products [185,230]. Such systems are suitable for real-time monitoring in diabetic care [229,230,231]. Moreover, functionalized hybrid hydrogels targeting cancer biomarkers like prostate-specific antigen (PSA) or carcinoembryonic antigen (CEA) have demonstrated potential for non-invasive cancer diagnostics. For instance, an immunosensor based on a PVA–alginate hydrogel matrix functionalized with anti-PSA antibodies showed high sensitivity and a detection threshold below clinical diagnostic level [254,255,256,257].

Hybrid hydrogels like collagen–PEG and gelatin–PEG systems have become clinically relevant because they combine biological recognition and mechanical precision. Their success lies in how the natural components mimic the ECM, while synthetic polymers provide structure, control, and reproducibility. These systems have moved from lab to clinical trials and even commercial applications, particularly in stem cell delivery, soft tissue engineering, ocular repair, and wound healing. Hybridization allows adding cell-adhesive properties to synthetic hydrogels, improving mechanical stability and injectability of natural gels, tuning degradation rates and crosslinking density [16,58,210,252].

CorMatrix ECM^®^ (CorMatrix Cardiovascular Inc., Sunnyvale, CA, USA), a clinically successful collagen–PEG hydrogel, is recommended for soft tissue engineering, in neural tissue repair (nerve guidance conduits), corneal scaffolds (ocular implants), and skin regeneration. From the point of view of synergistic mechanisms, type I or III collagen (from bovine or porcine origin) provides natural ECM structure and bioactive cues for cell attachment, migration, and differentiation, while PEG provides a controlled mesh size, regulates drug diffusion, and can be functionalized with ligands, drugs, or peptides. Dual crosslinking (physical from collagen and covalent from PEG) provides robust yet biocompatible scaffolds. Also, PEGDA or PEG–maleimide crosslinks the network and provides mechanical strength and modularity [16,58,210,252].

Gelatin–PEG hydrogels (e.g., GelMA) are widely used in TE, 3D bioprinting, and wound healing. Also, preclinical studies in cardiac patches and bone scaffolds are being finalized. Considering the synergistic mechanisms, gelatin (denatured collagen) provides cell adhesion, cell-friendly bioactivity and biodegradability, while PEG contributes to mechanical integrity, rapid curing, and tunable porosity. In addition, methacrylated PEG enables photopolymerization and mechanical tuning for real-time scaffold construction in 3D printing [16,58,210,252].

HyStem-C^®^ system (Glycosan/BioTime, Carlsbad, CA, USA), a HA–PEG hydrogel, reached Phase II/III clinical trials for ischemic limb disease and vocal cord repair and regeneration. It is also recommended for stem cell delivery and myocardial TE. Synergistic mechanisms highlight the use of HA, a naturally occurring glycosaminoglycan that mimics soft tissues ECM, for supporting cell adhesion and migration, while PEG allows precise control over stiffness and degradation. Also, PEG–vinyl sulfone or PEGDA enables in situ crosslinking and injectability [16,58,210,252].

In the hybrid systems, the synergistic mechanisms between natural and synthetic components provide biological signals, architecture and support, controlled degradation, tailored drug release, cell guidance, and processability. Natural polymers offer binding sites for bioactive molecules, and for this reason hybrid scaffolds allow incorporation of ligands, ECM motifs, or GFs while maintaining stability. Moreover, synthetic segments can slow degradation to match tissue healing: e.g., PEG controls mesh size, release rate and allows 3D printing, injectability, or sprayability [16,58,210,252].

### 4.4. Limitations and Future Perspectives

Despite their versatility, hybrid hydrogels face several challenges. Mechanical limitations persist in load-bearing applications, where they may underperform compared to purely synthetic materials. Additionally, their production requires meticulous control over polymer ratios, crosslinking strategies, and processing conditions, leading to scalability issues and high manufacturing costs. Biocompatibility concerns arise from the degradation products of synthetic components, which may provoke adverse reactions, necessitating comprehensive in vivo evaluations. The swelling behavior can be inconsistent under varying physiological conditions, complicating controlled drug release strategies. Moreover, their longevity may be limited if the degradation rate does not align with therapeutic needs. Moving forward, research should aim to enhance mechanical resilience, simplify synthesis protocols, and fine-tune degradation and release profiles. The integration of “smart” polymers and responsive elements into hybrid hydrogels holds promise for next-generation, adaptive biomaterials tailored to complex biomedical challenges [16,17,20,29].

Future directions of hybrid hydrogels include AI-guided design for personalized medicine, multifunctionality for response to pH, temperature, enzymes, or light, self-healing that mimic natural tissue regeneration, biofabrication of hybrid inks for 4D bioprinting and organ-on-chip platforms, and clinical formulations with off-the-shelf stability and custom in situ activation [16,58,210,252].

## 5. Stimuli-Responsive Hydrogels for Biomedical Applications

### 5.1. Overview

Stimuli-responsive hydrogels are crosslinked polymer networks that can swell, shrink, degrade, or change permeability in response to specific environmental triggers. The field is moving fast, especially with support from AI design tools and biofabrication technologies concerning temperature-, pH-, enzyme-, light-, electric field-, magnetic field-, and glucose-responsive hydrogels. Biomedical applications of stimuli-responsive hydrogels include: (*i*) DDS (temperature, pH, enzymes, glucose) with controlled, site-specific, or on-demand drug release; (*ii*) TE (temperature, enzymes) using injectable scaffolds and dynamic remodeling; (*iii*) wound healing (pH, enzymes) through release of antimicrobials or GFs in infected wounds; and (*iv*) biosensors (pH, glucose) applied for real-time sensing via swelling/degradation changes [33,34,35,36,38,39].

There are several challenges in clinical translation, such as stimulus specificity, reversibility, biocompatibility, and scalability. Many stimuli (pH, enzymes) are present in multiple body regions, so targeting can be imprecise. Some systems are of single use, creating reversible or repeatable responses technically harder. Certain responsive groups or byproducts, especially light- or electric-responsive, can be cytotoxic. Because of their complex synthesis and nanocomposite incorporation, stimuli-responsive hydrogels may be hard to mass-produce. Multi-responsive hydrogels, AI-assisted design, 3D/4D printing, and personalized medicine are the main future directions. Multi-responsive hydrogels combine two or more triggers (e.g., pH and temperature) for higher control and specificity. Also, ML models predict polymer behavior and optimize responsiveness. Complex, responsive structures for bioactuators or dynamic implants can be obtained using 3D/4D printing. Patient-specific hydrogels that respond to individual biochemical cues will be part of the broader concept of personalized medicine [33,34,35,36,38,39].

### 5.2. Temperature-Responsive Hydrogels

Temperature-responsive (thermoresponsive) hydrogels are polymeric networks that respond to temperature changes by swelling or contracting, transitioning between a sol (liquid) and gel (solid-like) state, altering their porosity, mechanical strength, or drug release rate. They exhibit a volume phase transition temperature (VPTT) often near body temperature (~37 °C). VPTT is the temperature at which a hydrogel undergoes a significant physical or chemical changes, usually swelling, shrinking, sol–gel transitions, or degradation. These hydrogels typically operate near the LCST, or upper critical solution temperature (UCST) of the polymers involved. LCST is a specific temperature below which a hydrogel is soluble in water and above which it becomes insoluble, leading to swelling. UCST is a temperature above which a hydrogel is soluble and below which it becomes insoluble, leading to shrinking [258,259,260,261].

Common materials used for fabrication include PNIPAAm, Poloxamers (Pluronic F127), chitosan–β-glycerophosphate, poly(*N*-vinylcaprolactam), PEO, polyvinylmethylether, and polyhydroxyethylmethacrylate. PNIPAAm (LCST ~32 °C, liquid at room temperature, gel at body temperature) is widely used in injectable hydrogels that solidify at body temperature for drug delivery or cell scaffolds. Pluronic F127 and chitosan–β-glycerophosphate (gel at body temperature) are recommended for ocular and nasal formulations and for stem cell delivery and injectable wound dressings, respectively. Poly(*N*-vinylcaprolactam) (LCST ~35–38 °C) exhibits better biocompatibility than PNIPAAm [258,259,260,261].

TRHs offer a powerful and minimally invasive platform for in situ therapies and targeted applications such as DDS (e.g., injectable implants), TE, wound dressing, biosensors, and microfluidics. Poloxamer-based gels are widely used for subcutaneous or intranasal delivery of peptides, nonsteroidal anti-inflammatory drugs (NSAIDs), or chemotherapeutics. For TE applications, TRHs support cell encapsulation (e.g., deliver of MSCs for cartilage or bone repair) and in situ scaffold formation. For wound dressing, gels cover wound, and release antibiotics or GFs in response to local temperature changes due to inflammation. For biosensors and microfluidics, TRHs are used in valves, actuators, or sensing layers in microfluidic devices that respond to temperature shifts. Minimal invasiveness, high control, biocompatibility, and adaptability are the main advantages. Injected as a liquid, these formulations form gel in the body. Also, many materials (like chitosan, poloxamers) are FDA-approved, and drug release can be tuned by temperature and easily combined with other stimuli (e.g., pH, enzymes) [258,259,260,261].

TRHs face several challenges such as narrow transition window, mechanical weakness, burst release, non-biodegradability, and thermal cytotoxicity. Gelation needs to occur close to body temperature, leading to high or low limits usability, and some systems release drugs too rapidly after gelation. Many are soft hydrogels with limited strength, unsuitable for load-bearing implants. Synthetic materials like PNIPAAm are not naturally degradable and require modification for in vivo use. Also, exothermic reactions during polymerization or gelation can affect cell viability if not well controlled. Future directions consider biodegradable thermogels, dual-responsive systems, 3D/4D bioprinting, and AI-guided material design. Modern techniques involve engineering PNIPAAm derivatives or natural polymer hybrids, combining temperature and pH or enzyme triggers for enhanced precision, using thermogels for scaffold printing that forms structure during or after printing, and predicting LCST behavior based on molecular structure and optimizing for clinical use [258,259,260,261].

### 5.3. pH-Responsive Hydrogels

pHRHs are polymer networks containing ionizable functional groups (–COOH or –NH_2_) that gain or lose protons depending on the pH environment. Protonation or deprotonation leads to changes in electrostatic repulsion, hydrophilicity, and crosslinking density, with impact on volume changes, pore size adjustment, or site-specific controlled drug release. Chitosan, PAA, poly(methacrylic acid), Eudragit^®^ (synthetic copolymers derived from esters of acrylic acid and methacrylic acid; Evonik, Essen, Germany), poly(*N*,*N*-dimethylaminoethyl methacrylate), and poly(ethyleneimine) are common polymers used for the fabrication of pHRHs. Chitosan, a natural cationic polymer, swells in tumor or wound pH (pH 5–6.5), and is recommended for cancer therapy and wound dressings. PAA, an anionic synthetic polymer ideal for oral drug delivery, swells in the intestine (pH ~6–8) but stays collapsed in the stomach (pH ~1–3) [262,263,264,265].

The main applications of pHRHs include DDS (e.g., enteric coatings), TE, wound healing, biosensors, and diagnostic devices. pHRHs can be used to target drug release to specific tissues or organs with different pH environments, like tumors or the GI tract. For oral drug delivery, pHRHs protect drugs from stomach acid and releases them in the intestine: e.g., for insulin delivery, PAA–methacrylate hydrogels release insulin in the duodenum, avoiding degradation in the stomach. For tumor-targeted therapy, the tumor microenvironment is more acidic (pH ~6.5) than normal tissues and pHRHs release chemotherapeutics selectively at tumor sites: chitosan–HA hydrogels for doxorubicin release in breast cancer chemotherapy. pHRHs ability to respond to pH changes can be used to create scaffolds that support cell growth and differentiation, as well as to deliver GFs. pHRHs can be triggered to release antimicrobials only in infected or inflamed wounds that often have acidic pH (5.5–6.5): e.g., pHRH dressings that release AgNPs or antibiotics in response to infection-driven pH changes. Changes in pHRH properties can be used to detect pH variations, which can be helpful for diagnostics and environmental monitoring. Also, hydrogels that change color, transparency, or conductivity in response to pH are used for wearable biosensors, contact lenses, and lab-on-a-chip devices [262,263,264,265].

pHRHs have three main advantages: controlled release, targeted delivery, and enhanced biocompatibility. They can deliver drugs or other substances at specific locations and rates based on the pH environment. Also, they can be designed to be effective in specific pH ranges, minimizing side effects in other areas. Many pHRHs are derived from natural materials, making them biocompatible. Several challenges need to be addressed for pHRHs, such as complexity, stability, limited pH range, biocompatibility, burst release, and manufacturing. Designing and synthesizing pHRHs with precise properties can be a complex process. Some pHRHs may not be stable over time, especially at extreme pH levels, and buffering in body fluids can mask small pH changes and reduce responsiveness. Best performance of pHRHs is often limited to pH 5–8, and extreme pH responsiveness is rare. Also, sudden pH shifts can cause rapid, uncontrolled drug release. Some synthetic pH-sensitive polymers can cause inflammation or immune reactions. The cost and scalability of manufacturing pHRHs can be a challenge. Future directions include dual-responsive hydrogels, AI-driven design, 3D/4D bioprinting, and personalized medicine. Dual-responsive hydrogels combine pH with temperature or enzyme sensitivity for more precise targeting. AI-driven design predicts and optimizes hydrogel formulations using ML models. 3D/4D bioprinting fabricates structures with pH zones for complex, multi-functional implants. Personalized medicine tailors pH-responsiveness to individual microbiome or disease profiles [262,263,264,265].

### 5.4. Enzyme-Responsive Hydrogels

Enzyme-responsive hydrogels (ERHs) are polymeric networks designed to undergo structural or chemical changes when exposed to specific enzymes. ERHs are engineered to respond to specific enzymes in their environment, either by swelling, degrading, or changing mechanical properties. Mechanisms of action include enzymatic degradation of crosslinkers, enzyme-triggered drug release, and enzyme-driven self-healing or remodeling. Enzyme cleaves specific peptide or sugar-based linkers, ERHs soften, swell, or disintegrate and release embedded drug or cells: e.g., PEG hydrogel with MMP-cleavable peptide crosslinked for tumor-specific drug delivery. ERHs either open pores or break down entirely in response to enzyme exposure and enable targeted, controlled delivery: e.g., HA hydrogels degraded by hyaluronidase in tumor microenvironments to release doxorubicin. In TE, where cells remodel their matrix, enzymes activate gelation or reformation of hydrogel via dynamic covalent bonds: e.g., MMP-degradable gels that allow cells to migrate and proliferate, promoting tissue regeneration [266,267,268,269].

Common enzymes used for ERHs fabrication include matrix metalloproteinases (MMPs), hyaluronidase, trypsin/elastase, lipase, and urease. They either break specific bonds in the hydrogel backbone or crosslinkers or modify side groups that alter swelling behavior or permeability. PEG, gelatin, HA, chitosan, dextran, and peptide crosslinkers are the main materials used for ERHs formulation. PEG, gelatin, and HA are common hydrogel backbones, peptide crosslinkers are custom-synthesized for enzyme specificity (e.g., MMP-cleavable sequences), and chitosan or dextran are natural polysaccharides sensitive to digestive and microbial enzymes [266,267,268,269].

In the biomedical field, ERHs are used for drug delivery (e.g., biodegradable implants, tumor targeting), precision TE and RM, wound healing, and biomedical devices (biosensing). ERHs can be used to target specific areas of the body, such as diseased tissues (cancer therapy), and trigger the release of drugs only in those areas. Hydrogels can be designed to be biocompatible and biodegradable, making them suitable for use in TE and RM applications: e.g., cell-laden hydrogels degrade in response to MMPs secreted by growing cells, supporting dynamic scaffolding that adapts as tissue forms. For wound healing, inflammatory enzymes (e.g., elastase) are used to trigger release of antimicrobials or GFs. ERHs can be used to create smart materials that can respond to biological signals and perform specific functions, such as hydrogels embedded with fluorophores that change signal upon enzyme-triggered cleavage [266,267,268,269].

The main advantages of ERHs refer to controlled release, targeted delivery, and biocompatibility. ERHs can be precisely designed to release drugs only in the presence and activity of the target enzyme, reducing side effects and improving treatment efficacy. Also, many ERHs are biocompatible and biodegradable, making them suitable for biomedical applications. Enzyme availability and variability, premature degradation, immunogenicity, and limited reuse fall into the challenges category. Different patients or tissues may have different enzyme levels, and circulating enzymes in blood or tissue could cause off-target hydrogel breakdown. In addition, some peptide- or protein-based gels can trigger immune responses. ERHs exhibit limited reuse—once degraded, the hydrogel often cannot be re-formed (non-reversible). Future directions are closely related to dual-/multi-ERHs, self-reporting hydrogels, personalized enzyme-targeted therapies, and injectable enzyme-triggered depots. Dual-/multi-ERHs ensure more precise targeting by requiring two enzyme signals: e.g., MMP–hyaluronidase. Self-reporting hydrogels emit a signal when degraded, useful for in vivo monitoring. Personalized enzyme-targeted therapies tailor hydrogel properties to patient-specific enzyme profiles using omics data. Long-term localized treatment for chronic diseases (e.g., arthritis, cancer, infections) can benefit from using injectable enzyme-triggered depots [266,267,268,269].

### 5.5. Light-Responsive Hydrogels

Light-responsive hydrogels (LRHs) are crosslinked polymer networks that respond to specific wavelengths of light, typically UV, VIS, or near-infrared (NIR), to induce swelling or shrinking, degradation, crosslinking or de-crosslinking, release of encapsulated drugs or molecules, and mechanical actuation or motion. Light-responsive mechanisms offer non-contact, spatiotemporal control. They include photoisomerization, photocleavage (photodegradation), photopolymerization/photo-crosslinking, and photothermal effects. Azobenzene change its *cis*–*trans* configuration when exposed to UV or VIS light, causing structural changes in the LRH, affecting stiffness, porosity, or swelling. Azobenzene-functionalized PAAm LRHs are used to reversibly bend or contract to alternating light wavelengths. Light triggers cleavage of specific chemical bonds in the hydrogel network, causing gel breakdown or payload release: e.g., PEG-based LRHs with nitrobenzyl ester crosslinkers degrade under UV light and release therapeutic proteins. Light initiates or strengthens crosslinking in the hydrogel for in situ gelation, 3D printing, and tissue scaffolding. GelMA crosslinked using blue light and photoinitiators (e.g., LAP or Irgacure) is used to create cell-laden scaffolds in TE. Embedded light-absorbing NPs, e.g., Au nanorods, GO, molybdenum disulfide (MoS_2_), absorb NIR light and convert it into heat that triggers volume change, gel softening, or drug release: e.g., AuNP-loaded hydrogels for NIR-triggered release of anticancer drugs [261,270,271,272].

Applications of LRHs refer to on-demand drug delivery, TE and RM, biosensing, soft robotics and actuators. Triggered release of drugs, proteins, or genes at precise time and location allows external control without surgery or direct contact: e.g., UV-degradable PEG LRHs releasing VEGF for wound healing. Light-based patterning, crosslinking, or cell placement for building complex tissues supports cell encapsulation and photopatterned gradients, such as 3D-printed GelMA LRHs used to construct vascularized bone tissue scaffolds. LRHs are used in optical biosensors that change transparency or fluorescence upon analyte detection. Light can induce movement, bending, or gripping via localized swelling or shape change enables remote-controlled bioinspired actuators: e.g., azobenzene-functionalized LRHs that walk or curl when exposed to patterned light [261,270,271,272].

Several challenges and limitations of LRHs must be considered, such as tissue penetration, phototoxicity, control of degradation, and stability of photoactive groups. UV and VIS light have limited depth in tissue (~1–2 mm), NIR being preferred for clinical use. Also, UV light and some photoinitiators can damage cells (cytotoxicity) or tissue, and over- or under-exposure can lead to incomplete or uncontrolled release. Some photoactive groups may degrade over time or require oxygen-free environments to function well. NIR-responsive hydrogels, multi-wavelength responsive systems, wireless control via wearable light devices, and AI-guided optimization can be considered the main future directions of LRHs. NIR-responsive hydrogels ensure better tissue penetration, safer than UV. Multi-wavelength responsive systems are recommended for layered, sequential control of drug delivery or shape change. Also, AI-guided optimization predicts and fine-tunes photoresponses based on material and light input variables [261,270,271,272].

### 5.6. Electric Field-Responsive Hydrogels

Electric field-responsive hydrogels (EFRHs), or electroresponsive hydrogels, are polymer networks that contain charged groups, conductive or magneto-sensitive NPs (e.g., Fe_3_O_4_, graphene). When an electric field is applied, it causes ion migration, electrochemical reactions, electroosmotic swelling or shrinking, electromechanical deformation. This results in contraction, bending, or shape changes that can be precisely controlled and reversed. Mechanisms of responsiveness include electrophoretic ion movement, electrochemical redox reactions and electromechanical actuation. Hydrogels with ionic groups such as –COOH or sulfonate move or reorient when exposed to an electric field. The ionic flow draws water in or out of the hydrogel, causing swelling or deswelling. EFRHs can include redox-active molecules (e.g., ferrocene) that undergo oxidation/reduction. This causes charge redistribution, altering swelling or structural properties. Incorporation of conductive polymers like PPy, polyaniline (PANI), or CNTs enables bending, contraction, or soft actuation for artificial muscles and soft robotic actuators. PPy, as a conductive backbone, responds rapidly to voltage, PANI is electrochromic and conductive, tunable by doping, PAA contains ionizable groups and swells under electric fields, hydrophilic PUs are used as flexible matrices, and GO/CNTs improve conductivity and mechanical strength [273,274,275].

EFRHs key applications include on-demand (pulsatile) drug delivery, TE, bioelectronic interfaces, soft robotics and artificial muscles. EFRHs can release drugs in response to applied voltage, such as PPy-coated hydrogels releasing dexamethasone for inflammation control in implanted neural devices. Electric field influence cell migration, alignment, and differentiation. EFRHs with embedded electrodes can serve as active scaffolds to guide tissue growth. Bioelectronic interfaces are used as soft, conductive interfaces between electronics and biological tissues for improved biocompatibility and signal transmission: e.g., EFRHs integrated with electroencephalogram, or electromyography electrodes for wearable biosensing. Because EFRHs bend, stretch, or contract under electric fields, they are used to mimic muscle movement in soft robotic actuators. Fo example, PANI-based hydrogel actuators are used in gripping devices, microvalves, and bioinspired robots [273,274,275].

Challenges and limitations of EFRHs refer to low mechanical strength, dehydration, biocompatibility, control complexity, and long-term stability. EFRHs are soft and may break under repeated actuation, and water loss can impair conductivity and responsiveness. Conductive additives (e.g., PPy, CNTs) can be cytotoxic at high levels. EFRHs require precise control of voltage, frequency, and duration. Also, degradation of redox agents or conductive polymers over time can influence long-term stability. Future directions of EFRHs are focused on wireless or implantable actuation, integration with sensors, multi-stimuli systems, and AI-guided optimization. Wireless or implantable actuation is using remote energy sources (e.g., inductive fields) to trigger drug release or movement inside the body. Integration with sensors creates feedback-controlled systems where hydrogel responds to both electrical and biological signals. Multi-stimuli systems combine electric responsiveness with pH, temperature, or light for more precise control. ML can be used to predict and optimize EFRHs behavior based on polymer structure and applied field parameters [273,274,275].

### 5.7. Magnetic Field-Responsive Hydrogels

Magnetic field-responsive hydrogels (MFRHs) are polymeric networks embedded with magnetic nanoparticles (MNPs), such as iron oxide (Fe_3_O_4_, maghemite (γ-Fe_2_O_3_)) or cobalt ferrite. When exposed to a magnetic field, the embedded NPs cause mechanical movement, heat generation (magnetothermal effect) and structural changes in hydrogel. Core mechanisms of action include magnetic actuation, magnetic hyperthermia and magneto-mechanical stimulation. External magnetic field causes hydrogel to move, deform, or vibrate for targeted positioning, shape change, or microvalve control. Alternating magnetic field causes localized heating via NPs friction (magnetic loss). The heat triggers hydrogel swelling, shrinking, or drug release. Magneto-mechanical stimulation can deliver mechanical signals to embedded cells (e.g., in tissue scaffolds), promoting differentiation or growth. Fe_3_O_4_ NPs are biocompatible and commonly used magnetic particles embedded in PEG, PVA, chitosan, and gelatin hydrogel matrices. SF and HA are used for fabrication of tissue-compatible or injectable systems. To maintain biocompatibility, NPs are usually coated with SiO_2_, dextran, or PEG [276,277,278,279].

Key applications of MFRHs include magnetically triggered drug delivery (targeted chemotherapy), neural and musculoskeletal TE, biosensing and diagnostics, soft robotics and actuators. MFRHs can release drugs in response to magnetic heating or field-induced structural change, offering site-specific, non-invasive control of therapeutic dosing. Tested in cancer models, Fe_3_O_4_–PNIPAAm hydrogel releases doxorubicin upon magnetic stimulation. Also, stem cell differentiation, especially in bone and nerve tissues (neuroregenerative implants) can be enhanced by magnetic stimulation. MFRHs act as scaffolds that deliver both mechanical cues and bioactive molecules: e.g., hydrogel scaffold embedded with MNPs to enhance osteogenesis in MSCs under magnetic fields. MFRHs change shape or electrical properties in response to a magnetic field and can be used to detect pathogens and monitor glucose levels. In addition, MFRHs act as responsive coatings for implants; they can bend, twist, or contract under magnetic fields to develop biocompatible soft robotic components (robotic surgery tools), microgrippers, and microswimmers [276,277,278,279].

Uniform distribution of MNPs, cytotoxicity, field penetration limits, heat control, and regulatory concerns are the most important challenges and limitations of MFRHs. Clumping of MNPs can impair responsiveness and make properties inconsistent, and uncoated or high-dose magnetic particles may be toxic to human cells. Magnetic field strength decreases with distance, deep tissue targeting being harder. Also, magnetothermal systems can overheat if not carefully tuned. MFRHs are still largely in preclinical or prototype stage, human safety not being fully vetted yet. Future directions of MFRHs include dual-responsive hydrogels, implantable systems, AI-guided modeling, and biohybrid devices. Dual-responsive MFRHs combine magnetic response with pH, temperature, or light for smarter control. Implantable systems refer to injectable MFRHs that release drugs on external command via wearable magnetic devices. AI-guided modeling uses ML to optimize hydrogel composition, NP loading, and field parameters. Biohybrid devices consider integration with living cells and electronics for advanced therapeutic systems (e.g., smart implants) [276,277,278,279].

### 5.8. Glucose-Responsive Hydrogels

Glucose-responsive hydrogels (GRHs) are polymeric networks that can swell, degrade, or change their permeability in response to glucose concentration. Enzyme-, phenylboronic acid (PBA)- and concanavalin A (ConA)-based systems are core mechanisms of glucose sensitivity, working in a physiological pH window (~7.4) if optimized. Glucose oxidase (GOx) catalyzes glucose transformation into gluconic acid. When local pH drops, it triggers hydrogel swelling, degradation, or pore opening. Chitosan, PAA, and PEG are common polymers used for GRHs fabrication. Acidification changes hydrogel structure, leading to insulin release: e.g., injectable chitosan–GOx hydrogels tested in diabetic mice, showing glucose-dependent insulin release for >48 h. PBA binds reversibly to glucose, forming charged complexes and altering hydrophilicity, swelling or degradation. ConA is a glucose-binding protein that can form reversible crosslinks. It competes with free glucose, altering network crosslinking and triggering insulin release: e.g., injectable microgel beads using ConA–glucose dynamics for pulsatile insulin release [280,281,282,283].

GRHs are used for self-regulating insulin delivery, glucose sensors, and oral insulin formulations. GRHs are especially promising for diabetes management, offering a pathway to self-regulated insulin delivery, mimicking pancreatic beta cells, offering glucose-triggered, feedback-regulated insulin release. Several challenges of GRHs must be considered, such as specificity and sensitivity, biocompatibility, long-term stability, insulin stability, and regulatory hurdles. GRHs must match physiological glucose range (70–180 mg/dL). Also, GOx-based systems may degrade over time or lose activity, and insulin can denature during loading or storage in the hydrogel. A very important aspect concerns the fact that some systems (e.g., ConA) can trigger immune reactions. Combination products including sensors and drug delivery formulations enter into a tougher approval pathway. Future directions of GRHs include dual-responsive hydrogels, AI-guided design, and closed-loop systems. Dual-responsive hydrogels respond to both glucose and pH for added precision, and ML can be used to optimize polymer–glucose interactions. Also, closed-loop systems integrate hydrogel sensors with wearable insulin pumps: e.g., injectable nano-hydrogel composites for minimally invasive, long-lasting glucose control [280,281,282,283].

## 6. Toxicity, Immunogenicity and Biocompatibility of Hydrogels

Environmental and metabolic fate of synthetic hydrogels depends largely on their chemical composition. In the environment, synthetic hydrogels can persist for long periods. Non-biodegradable hydrogels may accumulate in soil or aquatic systems, becoming persistent pollutants. Under UV exposure or oxidative conditions, they may fragment into microplastics or release residual monomers. Biodegradable hydrogels, often based on natural polymers, degrade via microbial action and are much more eco-friendly. Hydrogels are generally safe when fully polymerized and used appropriately, but incomplete reactions or in vivo degradation can yield toxic byproducts. Acrylamide leaching from poorly polymerized PAAm is a significant soil and water contaminant. Crosslinkers may degrade slowly and can be toxic to microorganisms and aquatic life. Persistence of some hydrogel polymers raises long-term questions about soil health and water filtration systems. Future trends focus on green hydrogels made from biopolymers or engineered to degrade safely [284,285].

Synthetic hydrogels are usually designed to be biocompatible, but their biodegradability varies. Degradable hydrogels are engineered using labile bonds (e.g., ester, disulfide, peptide), which can break down via hydrolysis or enzymatic cleavage. In the body, low-molecular-weight byproducts can cross biological membranes, may interfere with cellular function and are generally cleared via the kidneys, but larger fragments may trigger immune responses or accumulate in tissues if not readily excreted. PEG and PEG-based hydrogels are largely non-biodegradable in vivo unless modified (e.g., with ester or peptide linkages). PAAm and pHEMA are typically non-degradable, which limits their use to short-term applications unless they are functionalized. The toxicity of synthetic hydrogels depends on the monomers and crosslinkers used, and their degradation products (Table 7) [284,285].

PVA hydrogels face some important scalability challenges, including crosslinking constraints, sterility, biodegradability, and batch consistency. PVA is thermoplastic and non-degradable by default. It needs physical (e.g., freeze–thaw) or chemical crosslinking (e.g., with GAD, boric acid), many of which raise toxicity concerns. Physical methods are time-consuming and hard to scale consistently. Autoclaving can cause chain scission, while gamma irradiation can crosslink further or degrade PVA. Some sterilization methods that do not alter PVA mechanical or chemical properties are hard to find. Native PVA is non-biodegradable and accumulates in tissues unless modified. PVA has limited long-term in vivo applications unless it is used in non-permanent implants or excretable forms. PVA from different manufacturers can vary in degree of hydrolysis, molecular weight, and residual acetate content, with impact on swelling, mechanical strength, and drug release profiles, making regulatory approval harder [58,212,214].

Despite their versatility, PLA/PLGA hydrogels face several significant challenges. The accumulation of acidic byproducts during degradation can lower the local pH and compromise tissue compatibility, posing risks to surrounding tissues [15,57,220]. Furthermore, the toxicity associated with these degradation byproducts, particularly acidic moieties, has raised concerns regarding their impact on tissue health. Additionally, the complexity of hydrogel formulation presents a considerable challenge; designing hydrogels with optimal properties for swelling, crosslinking, and degradation is technically demanding and often incurs high costs [9,211,221].

Challenges for PU hydrogels include potential cytotoxicity from residual isocyanates or degradation byproducts, variable long-term stability, and cost intensive, multi-step synthesis. In this respect, hybrid systems that combine PUs with natural polymers like collagen and alginate are being explored to enhance bioactivity and biochemical interactions [57,146,211,212,213,214,215,216,217,218,219,220,221,229,230,231,232,233,234].

Natural hydrogels support cell adhesion, proliferation (e.g., collagen, fibrin), but have immunogenicity risk (xenogeneic proteins), limited mechanical strength. By comparison, synthetic hydrogels often lack biological signals (no integrin binding motifs). Functionalization with RGD or ECM peptides and composite or layered designs can improve cell compatibility and functionality of synthetic hydrogels. Natural hydrogels often degrade too quickly (e.g., gelatin dissolves in hours to days). Synthetic hydrogels (PEG, PVA) are not biodegradable and can accumulate unless specially modified. This affects drug release timing, implant resorption and tissue remodeling support. Hybrid or composite hydrogels can be used for biodegradability and chemical degradation control. Natural hydrogels face greater scrutiny from regulators for source, purity, and viral safety concerns, as follows: endotoxins—high risk (especially from animal-derived materials); long-term safety—often untested for synthetic degradation products; immunogenicity—for animal proteins (e.g., bovine collagen). Synthetic hydrogels can pose problems mainly regarding long-term safety: e.g., PEG byproducts (ethylene glycol) may be toxic [232,286,287].

Contemporary hydrogel systems now encompass a wide array of materials, including naturally derived biopolymers such as alginate, gelatin, collagen, HA, and chitosan, known for their excellent biocompatibility and biofunctionality, albeit with limitations in mechanical robustness. In contrast, synthetic hydrogels, including those based on PEG, PVA, PAAm, and poloxamers, offer enhanced mechanical strength, processability, and chemical tunability, though sometimes at the expense of biodegradability and cytocompatibility [15,24,25,26,27,28].

## 7. Regulatory Hurdles and Scalability Issues

### 7.1. Regulatory Hurdles

As hydrogels move from lab research to clinical and commercial applications, they face important regulatory and scalability challenges. These hurdles are critical for medical devices, DDS, and TE, where biocompatibility, manufacturing consistency, and compliance with global standards are essential for market entry. Therefore, hydrogels must pass rigorous norms for medical-grade formulations, biocompatibility testing requirements, and regulatory pathways involving International Organization for Standardization (ISO) rules (e.g., FDA) [69,288,289,290,291].

Several key ISO standards that apply for medical-grade hydrogels include the most important regulations, as follows: ISO 10993 (Biological evaluation of medical devices), a core standard for biocompatibility testing [292]; ISO 13485 (Medical devices—Quality Management System (QMS) requirements), mandatory for manufacturers’ quality systems [293]; ISO 11137 (Sterilization of health care products), required for radiation-sterilized hydrogels [294]; ISO 14971 (Risk management for medical devices), for identifying and mitigating product safety risks [295]; ISO 10993-6/-10/-11 [296] (Tests for local effects, irritation, and systemic toxicity), required for implantable or injectable hydrogels. For example, a PEG-based hydrogel for ocular application must pass ISO 10993-10 [297] (irritation) and ISO 10993-11 [298] (systemic toxicity) before entering human trials [69,288,289,290,291].

Biocompatibility testing requirements according to FDA and European Medicines Agency (EMA) rules, especially under ISO 10993, include cytotoxicity, sensitization and irritation, acute and chronic systemic toxicity, genotoxicity/carcinogenicity, implantation studies (for permanent materials), and hemocompatibility (if in contact with blood). Considering the duration of contact, type of tissue, and use-case (external dressing vs. brain implant), these tests can vary significantly [69,288,289,290,291].

Depending on use and formulation, hydrogels may fall under different FDA regulatory pathways: (*i*) Class I device for wound dressing, 510 (k) with predicate device; (*ii*) Class II device, injectable filler for drug-eluting hydrogel, 510 (k) and testing data; (*iii*) Class III device, spinal implant for brain hydrogel, premarket approval (PMA); (*iv*) Drug/biological hydrogel with active drug, requiring investigational new drug application (INDA) and new drug application (NDA)/biologics license application (BLA); (*v*) Combination product, drug and device (e.g., hydrogel-eluting stent), requiring coordinated review by the Center for Devices and Radiological Health (CDRH) and the Center for Drug Evaluation and Research (CDER) or multiple regulatory streams, increasing complexity and time-to-market [69,288,289,290,291].

### 7.2. Scalability Issues in Hydrogel Manufacturing

Despite their transformative promise, hydrogels face persistent challenges that hinder their full clinical translation. These include limited mechanical durability, complex and often cost-intensive fabrication methods, potential immunogenic responses, and the difficulty of scaling production for industrial use. Furthermore, regulatory approval processes demand extensive safety validation and long-term biocompatibility studies, which remain significant bottlenecks for the commercialization of hydrogel-based therapies [15,16,17,18,19,20,69,185,186,235].

4D bioprinting of hydrogels has some important challenges such as mechanical limitations, resolution vs. cell viability, stimulus compatibility, and regulatory hurdles. Most hydrogels are too soft for load-bearing applications, high print precision can sometimes hurt living cells, and some external triggers (like UV light) can harm tissue or cells. Therefore, clinical translation of 4D bioprinted hydrogels is still in early stage [46,47,48,49,50].

Limitations in clinical use of hydrogels refer to mechanical properties, sterilization issues, biodegradability, and regulatory approvals. Some hydrogels are too mechanically weak for load-bearing applications. Sterilization can be tricky without affecting performance, and biodegradability must be precisely timed for safety and efficacy. In this respect, hydrogels are still undergoing regulatory approval for many biomedical applications, such as neural or cancer therapy [20,58,59,60].

Barriers to commercialization of hydrogels in Phase II/III clinical trials include manufacturing scale-up, shelf stability, regulatory complexity, cost of goods, and market penetration challenges. Hydrogel properties are difficult to reproduce consistently at industrial scale mainly because crosslinking and polymerization techniques are sensitive to small changes in temperature, pH, or humidity, and lab methods often do not scale cleanly for FDA-compliant production. Some hydrogels require cold chain logistics or have short shelf-lives, which is problematic for global distribution. Also, controlled degradation of hydrogels is critical: if it is too fast can induce inflammation, but if it is too slow, fibrosis or poor integration may appear. Hydrogels are often classified as combination products (device and drug/cells) having complicated, longer, and more expensive approval pathways: e.g., FDA requires sterilization data, degradation byproduct safety, and detailed manufacturing controls. Also, hydrogels involving recombinant proteins, stem cells, or multi-step synthesis can be prohibitively expensive to mass-produce. Therefore, hospitals and clinics are slow to adopt new biomaterials unless there’s clear cost–benefit and reimbursement pathways [44,58,61,62].

Bringing hydrogel products from bench to clinic involves serious cost, reproducibility, and production challenges such as raw material cost and availability, batch-to-batch consistency, sterilization challenges, cell compatibility and functionality, biodegradability and degradation control, safety concerns, shelf-life and storage stability, quality assurance and GMP compliance (Table 8) [69,288,289,290,291].

Concerning raw material cost and availability, biopolymers like collagen or SF vary based on source, requiring extensive purification (to remove endotoxins, deoxyribonucleic acid (DNA), proteins) and standardization. GMP-grade polymers are expensive, especially if they require custom synthesis or modification: e.g., collagen can cost USD 500–2000/kg, depending on purity and origin; PEGDA can cost USD 500–1000/kg. Hydrogels are highly sensitive to molecular weight of components, crosslinking density, pH and ionic strength: e.g., natural hydrogels have often non-uniform crosslinking scalability (pH, enzyme, ion methods). Even small changes can drastically alter swelling, mechanical strength, or drug release. Therefore, crosslinking techniques must move toward scalable chemistries like click reactions, thermal gelation, or ionic crosslinking that are batch-tolerant, fast, and reproducible. For the control of batch-to-batch consistency, automated mixing, precise dosing, and real-time QC (e.g., rheometry, spectroscopy) are recommended [69,288,289,290,291].

Hydrogels are often sensitive to common sterilization methods. They can degrade or become toxic when exposed to autoclaving (heat damage), gamma radiation (chain scission), or EO (toxic residue). Autoclaving destroys natural polymers (denaturation, hydrolysis) and can deform synthetic hydrogels. Gamma irradiation causes chain scission (especially PEG, PVA) and loss of mechanical strength. EO leaves toxic residues that are hard to remove from porous hydrogels. UV sterilization has limited penetration depth and is ineffective for viscous or opaque hydrogels. Natural hydrogels like gelatin or collagen are particularly fragile compared with synthetic hydrogels like PEG or pHEMA, which are more resistant to sterilization but can still degrade. Sterile filtration (only for low-viscosity pre-gels), in situ gelation from sterile precursors, or aseptic manufacturing in cleanrooms are the main solutions to address sterilization challenges [232,286,287].

Hydrogels are often unstable at room temperature: e.g., water content leads to microbial contamination risk, crosslinked networks may dry out, shrink, unstable or even degrade, and enzyme-sensitive (natural) hydrogels may degrade during storage. Many hydrogels, especially injectable types, have short shelf-lives unless they are refrigerated or freeze-dried, requiring investment in cold chain logistics. Freeze-drying with rehydration at point-of-use, incorporation of preservatives (not always allowed for implantable hydrogels), or packaging in sterile, oxygen-free environments are the main solutions to address shelf-life and storage stability challenges [232,286,287]. Quality assurance and GMP compliance include full documentation of raw material traceability, process validation, sterility testing, end-product QC. This requires dedicated cleanroom manufacturing facilities, staff training, and regulatory audits [69,288,289,290,291].

## 8. Conclusions and Future Perspectives

Hydrogels have undoubtedly emerged as a transformative class of biomaterials, owing to their unique ability to mimic the natural ECM, absorb large quantities of water, and provide an adaptable platform for diverse biomedical applications. This review has elucidated the structural diversity, fabrication strategies, and application scope of both natural and synthetic hydrogels. Natural hydrogels offer superior biocompatibility and biodegradability, while synthetic variants provide greater mechanical tunability, reproducibility, and functional customization. The convergence of these materials with advanced engineering strategies has accelerated the development of “smart” hydrogels capable of responding to physiological stimuli, guiding cellular behavior, and delivering therapeutics with spatial and temporal precision.

Despite these advances, several critical limitations hinder the full clinical translation of hydrogel-based technologies. Natural hydrogels often suffer from weak mechanical integrity and batch-to-batch variability, whereas synthetic hydrogels may lack intrinsic bioactivity and often raise concerns regarding long-term biocompatibility and biodegradability. Additionally, residual monomers or crosslinking agents in synthetic hydrogels may present cytotoxicity risks if not properly purified. From a translational standpoint, challenges such as regulatory hurdles, scalability of production, and cost-effective manufacturing remain significant obstacles. Furthermore, many hydrogel systems are yet to be validated in complex in vivo environments, which may reveal unforeseen immunological or mechanical limitations.

The future of hydrogel technology lies in the development of multifunctional, hybrid systems that integrate the strengths of both natural and synthetic materials. The incorporation of nanomaterials, bioactive peptides, and genetically engineered components may yield next-generation hydrogels with enhanced mechanical strength, dynamic responsiveness, and improved cellular interactions. Advancements in 3D bioprinting and microfabrication techniques are expected to facilitate the design of personalized hydrogel scaffolds tailored to patient-specific anatomy and pathology. In parallel, “smart” hydrogels capable of real-time monitoring, self-healing, or sequential release of therapeutic agents offer exciting opportunities for precision medicine.

To realize the full potential of hydrogel technologies in clinical practice, interdisciplinary collaborations among materials scientists, bioengineers, and clinicians will be essential. Moreover, robust standardization protocols and comprehensive preclinical evaluations must be developed to ensure reproducibility, safety, and regulatory compliance. Continued research efforts should also prioritize green synthesis methods and the use of sustainable resources to meet the growing demand for environmentally responsible biomedical materials.

Hydrogels represent a pivotal frontier in biomedical innovation. While limitations remain, their immense versatility, combined with ongoing technological and scientific advancements, positions them at the forefront of future solutions in RM, DDS, and beyond. A balanced integration of material performance, biological compatibility, and clinical practicality will ultimately dictate the successful translation of hydrogel-based systems into next-generation healthcare technologies. Future directions are based on high-level interdisciplinary research, such as computer-guided hydrogel design (AI-driven formulation), intelligent programmable hydrogels, CRISPR-engineered bioinks, biohybrid systems, and sustainability-focused hydrogel design.

## Figures and Tables

**Table 1:** Comparative mechanical and physicochemical properties of natural hydrogels.

Hydrogel	Young’s Modulus (MPa)	Tensile Strength (MPa)	Swelling Ratio (% g/g)	Gelation Time at 37 °C (min)	In Vitro Degradation Time (Days)	Porosity (%)
collagen	0.001–0.1	0.01–0.15	1000–3000	30–60	7–30	70–95
gelatin	0.01–0.1	0.05–0.3	500–1500	5–15	5–20	70–90
fibrin	0.0005–0.005	0.001–0.01	900–1200	5–10	2–7	80–95
albumin	0.001–0.06	0.01–0.05	400–800	5–20	5–15	60–80
silk fibroin	0.5–5	0.5–2	200–600	15–30	20–60	60–85
sericin	0.005–0.03	0.005–0.02	800–1500	8–10	3–10	70–90
SPI	0.1–0.6	0.1–0.4	300–800	20–30	10–25	60–85
pea protein	0.005–0.3	0.05–0.2	200–600	20–40	7–15	60–80
wheat gluten	0.3–1.5	0.2–0.8	100–400	30–60	15–40	50–75
chitin	0.1–0.5	0.1–0.4	100–300	N/A	20–60	50–80
chitosan	0.01–0.5	0.05–0.2	300–800	5–20	7–30	60–90
HA	0.001–0.1	0.02–0.1	500–1000	5–10	3–14	70–95
alginate	0.02–0.1 (Ca^2+^ gel)	0.03–0.2	700–1500	1–5 (Ca^2+^ gel)	5–14	70–90
carrageenan	0.001–0.1	0.01–0.8	600–1200	5–10	5–20	65–85
cellulose	0.1–2	0.2–5	100–400	15–30	10–60	60–90
starch	0.1–0.8	0.1–1	200–600	20–40	5–20	50–80
xanthan gum	0.01–0.2	0.05–0.3	800–2000	3–4	7–15	60–85
dextran	0.002–0.3	0.1–0.4	300–700	5–10	5–21	60–90
pullulan	0.01–0.1	0.05–0.2	400–1000	5–15	7–14	60–85

HA: hyaluronic acid; N/A: not applicable; SPI: soy protein isolate.

**Table 2:** Advantages, limitations, and key applications of natural hydrogels.

Hydrogel	Advantages	Limitations	Key Applications
collagen	▪ excellent biocompatibility; ▪ natural ECM component; ▪ promotes cell adhesion and growth.	▪ low mechanical strength; ▪ rapid degradation by collagenases; ▪ batch variability.	▪ skin/TE; ▪ corneal repair; ▪ wound healing.
gelatin	▪ high biocompatibility (derived from collagen); ▪ cell-friendly (retains RGD motifs for cell attachment); ▪ thermoresponsive.	▪ poor mechanical strength; ▪ melting at body temperature (without crosslinking); ▪ degraded by MMPs and pepsin.	▪ drug delivery; ▪ 3D bioprinting; ▪ TE.
fibrin	▪ excellent biocompatibility; ▪ promotes cell migration and angiogenesis; ▪ hemostatic.	▪ weak mechanical properties; ▪ rapid degradation by plasmin.	▪ vascular and neural TE; ▪ wound healing; ▪ tissue sealants.
albumin	▪ high biocompatibility (non-toxic, present in plasma); ▪ readily available; ▪ can bind drugs efficiently; ▪ slowly biodegradable (1–2 weeks, depends on crosslinking).	▪ poor mechanical properties; ▪ limited structural support.	▪ drug delivery; ▪ wound dressing; ▪ injectable hydrogels.
silk fibroin	▪ excellent biocompatibility; ▪ strong mechanical properties; ▪ supports cell growth and tissue integration; ▪ slowly biodegradable by proteases (over weeks to months).	▪ complex processing; ▪ batch variability depending on source.	▪ bone/cartilage TE; ▪ 3D scaffolds; ▪ wound dressing.
sericin	▪ high biocompatibility; ▪ promotes cell proliferation; ▪ antioxidant, anti-inflammatory, supports skin regeneration; ▪ moderate biodegradable by proteases (1–2 weeks).	▪ poor mechanical strength (without blending); ▪ soluble in water (less stable).	▪ drug delivery; ▪ skin regeneration; ▪ cosmetic applications.
SPI	▪ high biocompatibility; ▪ non-toxic, supports moderate cell attachment; ▪ good gelling properties; ▪ moderate biodegradable (enzymes or hydrolysis, 1–3 weeks).	▪ allergenicity (high protein content, ~90%); ▪ poor mechanical strength (unless crosslinked); ▪ pH-sensitive.	▪ drug delivery; ▪ 3D scaffolds; ▪ wound healing; ▪ edible films.
pea protein	▪ high biocompatibility; ▪ hypoallergenic, non-toxic, supports limited cell adhesion; ▪ good water retention; ▪ completely biodegradable, slower than SPI (2–4 weeks, depending on formulation).	▪ needs modification to improve gel mechanical properties; ▪ lower solubility than SPI.	▪ biodegradable films; ▪ functional foods; ▪ edible packaging.
wheat gluten	▪ high biocompatibility (edible and non-toxic); ▪ strong film-forming ability (elastic and cohesive); ▪ promotes healing (glutamine-rich content); ▪ moderate biodegradable (2–6 weeks, dependent on processing and crosslinking).	▪ allergenic; ▪ poor solubility in water; ▪ susceptible to enzymatic degradation	▪ tissue scaffolds (with modification); ▪ wound dressing; ▪ food coatings.
chitin	▪ high biocompatibility; ▪ low immunogenicity; ▪ supports cell adhesion; ▪ naturally derived antimicrobial; ▪ slowly biodegradable by chitinase (weeks to months).	▪ poor solubility in water and common solvents; ▪ limited processability.	▪ tissue scaffolds; ▪ antibacterial dressings; ▪ wound healing.
chitosan	▪ excellent biocompatibility; ▪ derived from chitin (more soluble); ▪ antimicrobial, hemostatic, mucoadhesive; ▪ promotes cell proliferation; ▪ moderate biodegradable by lysozyme, chitosanase (1–4 weeks).	▪ limited mechanical strength; ▪ pH-sensitive gelation (soluble in acidic conditions only).	▪ drug delivery; ▪ gene delivery; ▪ TE; ▪ wound healing.
HA	▪ excellent biocompatibility; ▪ native ECM component; ▪ promotes cell migration, proliferation and tissue repair; ▪ excellent water retention.	▪ needs crosslinking to improve stability; ▪ rapid enzymatic degradation (by hyaluronidase, 2–14 days in vitro).	▪ cartilage repair; ▪ dermal fillers; ▪ wound healing.
alginate	▪ high biocompatibility; ▪ non-immunogenic; ▪ easy to process by gelation with divalent cations (e.g., Ca^2+^); ▪ non-degradable by mammals (ion exchange or chemical modification for breakdown).	▪ unstable in physiological conditions (unless crosslinked); ▪ poor cell adhesion; ▪ partially degraded by lyase.	▪ drug delivery; ▪ cell encapsulation; ▪ TE; ▪ wound dressing.
carrageenan	▪ high biocompatibility; ▪ edible and generally safe (derived from red seaweed); ▪ thermoreversible gelation; ▪ slow and limited biodegradability (not easily degraded enzymatically in mammals).	▪ mild inflammatory response in high purity forms; ▪ poor mechanical strength; ▪ sensitive to ionic environment and pH.	▪ drug delivery; ▪ TE; ▪ food industry (gelling agent).
cellulose	▪ high biocompatibility; ▪ non-toxic, abundant and renewable; ▪ chemically modifiable (e.g., CMC, HPMC); ▪ slow to moderate biodegraded by cellulases, depending on form and derivatization.	▪ insoluble in water (native form); ▪ requires chemical modification for hydrogel preparation.	▪ drug delivery; ▪ tissue scaffolds; ▪ wound dressing; ▪ food packaging.
starch	▪ high biocompatibility; ▪ edible and non-toxic; ▪ GRAS; ▪ widely available and low-cost.	▪ poor mechanical strength; ▪ re-gelling and water sensitivity; ▪ fast enzymatic degradation by amylases (days to weeks).	▪ drug delivery (controlled release systems); ▪ food films.
xanthan gum	▪ high biocompatibility; ▪ stable over wide pH and temperature range; ▪ high viscosity at low concentration; ▪ shear-thinning (injectables/printing); ▪ moderate to slow biodegraded by xanthanases.	▪ weak mechanical strength; ▪ blending and crosslinking are mandatory for structural applications.	▪ injectable hydrogels; ▪ bioinks for 3D bioprinting; ▪ food thickener.
dextran	▪ excellent biocompatibility; ▪ non-immunogenic; ▪ water-soluble, easily functionalized; ▪ good film-forming ability.	▪ limited mechanical properties (unless crosslinked); ▪ moderate to fast degradation by dextranase (1–2 weeks).	▪ drug delivery (nanoparticles, gels); ▪ 3D scaffolds; ▪ injectable hydrogels.
pullulan	▪ high biocompatibility; ▪ low immunogenicity; ▪ edible, non-toxic, film-forming; ▪ oxygen barrier properties; ▪ moderate biodegraded by pullulanase and gut flora.	▪ poor mechanical strength (unless blended); ▪ moisture sensitivity.	▪ drug delivery; ▪ wound healing (films); ▪ edible coatings.

3D: three-dimensional; CMC: carboxymethyl cellulose; ECM: extracellular matrix; GRAS: Generally Recognized as Safe; HA: hyaluronic acid; HPMC: hydroxypropyl methylcellulose; SPI: soy protein isolate; MMPs: matrix metalloproteinases; RGD: arginine–glycine–aspartic acid; TE: tissue engineering.

**Table 3:** Comparative mechanical and physicochemical properties of synthetic hydrogels.

Hydrogel	Young’s Modulus (MPa)	Tensile Strength (MPa)	Swelling Ratio (% g/g)	Gelation Time at 37 °C (min)	In Vitro Degradation Time (Days)	Porosity (%)
PAAm	0.01–0.5	0.1–0.5	500–1500	5–15	ND (native form)	60–90
PEG	0.1–0.3	0.05–0.3	100–600	1–10	ND (unless modified)	60–90
PVA	0.1–0.8	0.2–1	200–400	15–30	PD	60–85
PAA	0.05–0.5	0.1–0.5	1000–3000	10–20	Variable *	60–90
Poloxamer (Pluronic F127)	0.001–0.15	0.01–0.1	500–1200	2–5	PD	70–95
PNIPAAm	0.01–0.3	0.01–0.1	500–1500	1–5	ND **	60–90
PLA	100–1000 (solid)	20–60 (solid)	<100	N/A	30–180	30–70
PLGA	1–100 (bulk form)	5–50	<100	N/A	7–60	30–80
PU	0.1–10	0.1–25	200–600	5–30	Variable *	50–90

* Slow unless modified; ** Unless copolymerized; N/A: not applicable; ND: non-degradable; PAA: poly(acrylic acid); PAAm: poly(acrylamide); PD: poorly degradable; PEG: poly(ethylene glycol); PLA: poly(lactic acid); PLGA: poly(lactic-*co*-glycolic) acid; PNIPAAm: poly(*N*-isopropylacrylamide); PVA: poly(vinyl alcohol); PU: polyurethane.

**Table 4:** Advantages, limitations, and key applications of synthetic hydrogels.

Hydrogel	Advantages	Limitations	Key Applications
PAAm	▪ moderate to high biocompatibility; ▪ inert, low toxicity when purified; ▪ easily crosslinked; ▪ high water retention; ▪ transparent and soft.	▪ residual monomers may be toxic; ▪ not biodegradable (copolymerization or additives are mandatory for degradability).	▪ tissue models; ▪ wastewater treatment; ▪ sensors; ▪ soft robotics.
PEG	▪ excellent biocompatibility; ▪ non-immunogenic, cell-inert; ▪ FDA-approved; ▪ easily functionalized by crosslinking.	▪ low cell adhesion without biofunctionalization; ▪ not biodegradable (native PEG), unless modified with degradable blocks.	▪ drug delivery; ▪ TE; ▪ injectable hydrogels; ▪ anti-fouling coatings.
PVA	▪ high biocompatibility; ▪ non-toxic; ▪ good mechanical strength; ▪ repeated freeze–thaw gelation.	▪ limited bioactivity (inert to cells, unless modified); ▪ poorly biodegradable (not easily broken down in vivo).	▪ drug delivery; ▪ cartilage repair; ▪ wound dressing; ▪ contact lenses.
PAA	▪ good to high biocompatibility; ▪ generally safe; ▪ high water absorbency; ▪ pH-sensitive swelling.	▪ brittle when dry; ▪ poor mechanical properties (without blending); ▪ limited biodegradability, not enzymatically degradable, slow hydrolysis (unless modified).	▪ oral drug delivery; ▪ diapers and hygiene products.
Poloxamer (Pluronic F127)	▪ excellent biocompatibility; ▪ thermoresponsive (sol–gel transition around body temperature).	▪ poor mechanical strength; ▪ rapid dissolution in aqueous environments; ▪ not biodegradable (but cleared renally).	▪ drug solubilization; ▪ thermoresponsive drug delivery; ▪ injectable gels; ▪ ophthalmic and dermal products.
PNIPAAm	▪ moderate to good biocompatibility (cell-inert); ▪ thermoresponsive (LCST ~32 °C); ▪ reversible sol–gel transition near body temperature; ▪ tunable swelling.	▪ poor mechanical strength; ▪ requires copolymerization for stability; ▪ non-biodegradable in native form (requires copolymerization for biodegradability).	▪ controlled drug release; ▪ cell sheet engineering; ▪ injectable gels.
PLA	▪ high biocompatibility; ▪ minimal immune response; ▪ FDA-approved; ▪ good mechanical strength.	▪ brittle; ▪ hydrophobic (limited swelling); ▪ slow biodegradation by hydrolysis to LA (weeks to months).	▪ drug delivery (e.g., microspheres); ▪ bone and tissue 3D scaffolds; ▪ implants, sutures.
PLGA	▪ excellent biocompatibility; ▪ FDA-approved; ▪ fully biodegradable, faster than PLA (tunable degradation, 7–60 days, by LA/GA ratio).	▪ inflammation caused by acidic degradation products; ▪ hydrophobic.	▪ drug delivery (e.g., sustained-release injectables, drug encapsulation); ▪ TE.
PU	▪ good to high biocompatibility (when medical grade); ▪ hemocompatible; ▪ mechanical tunable depending on chemistry (highly elastic and durable).	▪ some formulations involve toxic precursors (e.g., isocyanates); ▪ non-biodegradable or slowly biodegradable (unless specifically designed).	▪ soft tissue implants; ▪ cardiac and bone 3D scaffolds; ▪ wound dressing.

3D: three-dimensional; FDA: Food and Drug Administration; GA: glycolic acid; LA: lactic acid; LCST: lower critical solution temperature; PAA: poly(acrylic acid); PAAm: poly(acrylamide); PEG: poly(ethylene glycol); PLA: poly(lactic acid); PLGA: poly(lactic-*co*-glycolic) acid; PNIPAAm: poly(*N*-isopropylacrylamide); PVA: poly(vinyl alcohol); PU: polyurethane; TE: tissue engineering.

**Table 5:** Comparative mechanical and physicochemical properties of hybrid hydrogels.

Hydrogel	Young’s Modulus (MPa)	Tensile Strength (MPa)	Swelling Ratio (% g/g)	Gelation Time at 37 °C (min)	In Vitro Degradation Time (Days)	Porosity (%)
Collagen–PCL	1–5	1–3	<100	N/A	30–90	60–75
Gelatin–PVA	0.05–0.3	0.02–0.1	500–1000	10–20	15–30	70–90
GelMA	0.01–0.05	0.005–0.02	200–500	2–3 (UV)	10–20	60–90
Silk fibroin– gelatin	0.5–2	0.8–1.5	400–700	10–20	20–40	60–85
Chitosan–PEGDA	0.2–1.5	0.5–1.2	100–300	5–10	7–21	60–85
Chitosan– Pluronic F127	0.05–0.15	0.02–0.08	300–600	2–5	10–20	70–90
HA–PEG	0.03–0.1	0.02–0.08	200–400	2–3	10–20	70–80
Alginate– gelatin	0.02–0.1	0.01–0.08	800–1200	3–5	5–14	75–90
Alginate–PVA	0.03–0.2	0.01–0.06	500–900	10–15	10–25	70–85
PVA–starch	0.2–0.5	0.1–0.3	200–400	15–30	15–30	50–80

GelMA: gelatin–methacrylate; HA: hyaluronic acid; N/A: not applicable; PCL: poly(ε-caprolactone); PEG: poly(ethylene glycol); PEGDA: poly(ethylene glycol) diacrylate; PVA: poly(vinyl alcohol); UV: ultraviolet.

**Table 6:** Advantages, limitations, and key applications of hybrid hydrogels.

Hydrogel	Advantages	Limitations	Key Applications
Collagen–PCL	▪ high biocompatibility; ▪ collagen supports cell adhesion and proliferation; ▪ PCL is well-tolerated; ▪ excellent mechanical strength (from PCL); ▪ good structural integrity.	▪ requires organic solvents or pre-processing; ▪ not injectable; ▪ slow to moderate biodegradability: collagen degrades enzymatically, PCL degrades very slow (months to years).	▪ nerve guidance scaffolds; ▪ bone and vascular tissue engineering; ▪ skin grafts.
Gelatin–PVA	▪ high biocompatibility; ▪ low cytotoxicity (gelatin promotes cell adhesion, PVA is non-toxic but inert); ▪ improved elasticity over pure gelatin.	▪ poor thermal stability (unless crosslinked); ▪ requires freeze–thaw cycles for gelation; ▪ partial biodegradability: gelatin is biodegradable, PVA is not easily degradable in vivo.	▪ drug delivery films; ▪ soft tissue scaffolds; ▪ wound healing.
GelMA	▪ high biocompatibility; ▪ supports good cell adhesion and viability (cell encapsulation); ▪ supports 3D bioprinting; ▪ photo-crosslinkable (tunable stiffness).	▪ limited mechanical strength without additives; ▪ requires UV light and photoinitiator; ▪ moderate biodegradability: enzymatic degradation (depending on degree of methacrylation and crosslinking).	▪ 3D bioprinting; ▪ cell-laden constructs; ▪ tissue models (cardiac, cartilage, skin).
Silk fibroin– gelatin	▪ high biocompatibility; ▪ promotes cell adhesion and growth (both components); ▪ strong mechanical properties (from silk fibroin).	▪ silk fibroin may vary by source; ▪ requires careful processing and sterilization; ▪ moderate biodegradability: silk fibroin degrades slowly, gelatin degrades enzymatically.	▪ nerve tissue engineering; ▪ bone and cartilage regeneration; ▪ scaffolds for load-bearing tissues.
Chitosan– PEGDA	▪ high biocompatibility; ▪ chitosan supports cell adhesion; ▪ PEGDA is non-toxic; ▪ photo-crosslinkable for rapid gelation; ▪ tunable mechanical strength.	▪ limited elasticity; ▪ chitosan is soluble only in acidic pH; ▪ requires UV light and photoinitiator; ▪ variable biodegradability: chitosan degrades enzymatically, PEGDA is non-degradable (unless modified).	▪ drug delivery; ▪ gene delivery; ▪ cartilage and soft tissue engineering; ▪ 3D cell culture.
Chitosan– Pluronic F127	▪ high biocompatibility; ▪ enhanced mucoadhesiveness and bioactivity (from chitosan); ▪ injectable; ▪ thermoresponsive (at ~37 °C).	▪ weak mechanical strength (unless reinforced); ▪ fast erosion in aqueous media; ▪ partial biodegradability: chitosan degrades enzymatically; Pluronic is not biodegradable (renally cleared).	▪ mucosal and injectable drug delivery; ▪ localized cancer therapy; ▪ wound healing.
HA–PEG	▪ high biocompatibility; ▪ non-immunogenic and cell-friendly (HA is part of native ECM, PEG is non-toxic); ▪ good water retention; ▪ tunable stiffness and degradation (via PEG structure).	▪ requires chemically modified HA; ▪ may quickly degrade without crosslinking; ▪ partial biodegradability: HA degrades enzymatically (hyaluronidase); PEG is non-degradable (unless modified).	▪ injectable tissue scaffolds; ▪ cartilage regeneration; ▪ ophthalmology.
Alginate– gelatin	▪ high biocompatibility; ▪ supports cell adhesion (gelatin); ▪ alginate is non-toxic; ▪ combines bioactivity (gelatin) with gel strength (alginate); ▪ thermo- and ion-sensitive (Ca^2+^ crosslinking).	▪ weak mechanical strength without reinforcement; ▪ unstable in physiological fluids (with no additional crosslinking); ▪ moderate biodegradability: alginate degrades in physiological fluids; gelatin degrades enzymatically.	▪ drug delivery; ▪ 3D bioprinting; ▪ cell encapsulation; ▪ wound healing.
Alginate–PVA	▪ high biocompatibility; ▪ alginate and PVA are non-toxic; ▪ improved mechanical strength over pure alginate; ▪ flexible and elastic with freeze–thaw cycles.	▪ limited cell adhesion (from PVA); ▪ requires controlled freeze–thaw or chemical crosslinking; ▪ partial biodegradability: alginate degrades in physiological fluids; PVA is not inherently biodegradable.	▪ tissue scaffolds; ▪ wound dressing; ▪ soft actuators; ▪ food packaging.
PVA–starch	▪ high biocompatibility; ▪ edible, low-cost, non-toxic and safe (both components); ▪ good water absorbency and swelling.	▪ poor mechanical stability without crosslinking; ▪ sensitive to moisture and enzymatic degradation; ▪ variable biodegradability: PVA is not biodegradable (unless modified); starch is biodegradable.	▪ drug delivery matrices; ▪ wound healing films; ▪ edible packaging.

3D: three-dimensional; ECM: extracellular matrix; GelMA: gelatin–methacrylate; HA: hyaluronic acid; PCL: poly(ε-caprolactone); PEG: poly(ethylene glycol); PEGDA: poly(ethylene glycol) diacrylate; PVA: poly(vinyl alcohol); UV: ultraviolet.

**Table 7:** Potential toxicity of synthetic hydrogels’ degradation byproducts.

Synthetic Material	Degradation Byproducts	Toxicity
PAAm	acrylamide (if unreacted)	neurotoxic, carcinogenic (group 2A IARC)
PEG	ethylene glycol (incomplete degradation)	toxic at high doses (renal failure)
PNIPAAm	isopropylacrylamide derivatives	cytotoxic at high levels
pHEMA	methacrylic acid and its ester derivatives	low acute toxicity (prolonged exposure may irritate tissues)
Crosslinkers (e.g., GAD)	aldehydes, epoxides	cytotoxic and sensitizing

GAD: glutaraldehyde; IARC: International Agency for Research on Cancer; PAAm: poly(acrylamide); PEG: poly(ethylene glycol); pHEMA: poly(2-hydroxyethyl methacrylate); PNIPAAm: poly(*N*-isopropylacrylamide).

**Table 8:** Properties and limitations of natural vs. synthetic hydrogels.

Properties/Limitations	Natural Hydrogels	Synthetic Hydrogels
biocompatibility	excellent	good to excellent (polymer-dependent)
biodegradability	often too fast (enzyme-mediated)	often not biodegradable (unless engineered)
biological activity	high	low (unless functionalized)
mechanical strength	poor to moderate	moderate to excellent
reproducibility	variable (source-dependent)	high (chemically defined)
endotoxins	high risk (especially from animal-derived materials)	low risk
immunogenicity	high risk for animal proteins (e.g., bovine collagen)	low risk (unless impurities remain)
long-term safety	often untested for synthetic degradation products	PEG byproducts (ethylene glycol) may be toxic
sterilization compatibility	poor	moderate
crosslinking scalability	often non-uniform (enzyme, ionic, pH methods)	UV light or click chemistry (expensive)
shelf-life stability	short (weeks to months)	moderate (months to years)
batch consistency	low	high
regulatory risk	high (pathogens, endotoxins)	moderate
cost	moderate to high (due to extraction and purification)	moderate to high (GMP polymers mainly)

GMP: Good Manufacturing Practice; PEG: poly(ethylene glycol); UV: ultraviolet.

## Data Availability

The original contributions presented in this study are included in the article. Further inquiries can be directed to the corresponding author.

## Abbreviations

The following abbreviations are used in this manuscript:

3D	Three-dimensional
4D	Four-dimensional
Ag	Silver
AgNPs	Silver nanoparticles
AI	Artificial intelligence
AIBN	Azobisisobutyronitrile
Au	Gold
BLA	Biologics license application
BME	Biomedical engineering
CD44	Cluster of differentiation 44
CDER	Center for Drug Evaluation and Research
CDRH	Center for Devices and Radiological Health
CEA	Carcinoembryonic antigen
CG	Carrageenan
CMC	Carboxymethyl cellulose
CNCs	Cellulose nanocrystals
CNFs	Cellulose nanofibers
CNTs	Carbon nanotubes
ConA	Concanavalin A
CRISPR	Clustered regularly interspaced short palindromic repeats
DC	Degree of crosslinking
DD	Degree of deacetylation
DDS	Drug delivery systems
Dex	Dextran
DexMA	Dextran–methacrylate
DL	Deep learning
DNA	Deoxyribonucleic acid
DP	Degree of polymerization
ECH	Epichlorohydrin
ECM	Extracellular matrix
EDC	1-Ethyl-3-(3-dimethylaminopropyl) carbodiimide
EFRHs	Electric field-responsive hydrogels
ELP	Elastin-like polypeptide
EMA	European Medicines Agency
EO	Ethylene oxide
ERHs	Enzyme-responsive hydrogels
EU	European Union
FDA	Food and Drug Administration
Fe_3_O_4_	Magnetite
γ-Fe_2_O_3_	Maghemite
G	α-L-guluronic acid
GA	Glycolic acid
GAD	Glutaraldehyde
GelMA	Gelatin–methacrylate
GFs	Growth factors
GI	Gastrointestinal
GlcNAc	*N*-acetyl-D-glucosamine
GMP	Good Manufacturing Practice
GNP	Genipin
GO	Graphene oxide
GOx	Glucose oxidase
GRAS	Generally Recognized as Safe
GRHs	Glucose-responsive hydrogels
H_2_O_2_	Hydrogen peroxide
HA	Hyaluronic acid
HPMC	Hydroxypropyl methylcellulose
HRP	Horseradish peroxidase
HSA	Human serum albumin
IARC	International Agency for Research on Cancer
INDA	Investigational new drug application
ISO	International Organization for Standardization
LA	Lactic acid
LCST	Lower critical solution temperature
LRHs	Light-responsive hydrogels
M	β-D-mannuronic acid
MBAm	*N*,*N*′-methylenebisacrylamide
MCC	Microcrystalline cellulose
MFRHs	Magnetic field-responsive hydrogels
ML	Machine learning
MMPs	Matrix metalloproteinases
MNPs	Magnetic nanoparticles
MoS_2_	Molybdenum disulfide
MRI	Magnetic resonance imaging
mRNA	Messenger ribonucleic acid
MSCs	Mesenchymal stem cells
N/A	Not applicable
ND	Non-degradable
NDA	New drug application
NHS	*N*-hydroxysuccinimide
NIPAAm	*N*-isopropylacrylamide
NIR	Near-infrared
NPs	Nanoparticles
NSAIDs	Nonsteroidal anti-inflammatory drugs
PAA	Poly(acrylic acid)
PAAm	Poly(acrylamide)
PANI	Polyaniline
PBA	Phenylboronic acid
PCL	Poly(ε-caprolactone)
PD	Poorly degradable
PEDOT	Poly(3,4-ethylenedioxythiophene)
PEG	Poly(ethylene glycol)
PEGDA	Poly(ethylene glycol) diacrylate
PEO	Poly(ethylene oxide)
pHEMA	Poly(2-hydroxyethyl methacrylate)
pHRHs	pH-responsive hydrogels
PLA	Poly(lactic acid)
PLGA	Poly(lactic-*co*-glycolic) acid
PMA	Premarket approval
PNIPAAm	Poly(*N*-isopropylacrylamide)
PO	Propylene oxide
PPO	Poly(propylene oxide)
PPy	Polypyrrole
PSA	Prostate-specific antigen
PSS	Polystyrene sulfonate
PU	Polyurethane
PVA	Poly(vinyl alcohol)
QC	Quality control
QMS	Quality Management System
RGD	Arginine–glycine–aspartic acid
RHAMM	Receptor for hyaluronic acid-mediated motility
RM	Regenerative medicine
SF	Silk fibroin
SiO_2_	Silica
SPI	Soy protein isolate
STPP	Sodium tripolyphosphate
TE	Tissue engineering
TG	Transglutaminase
TR	Tissue repair
TRHs	Thermoresponsive hydrogels
UCST	Upper critical solution temperature
UV	Ultraviolet
VEGF	Vascular endothelial growth factor
VIS	Visible
VPTT	Volume phase transition temperature
XG	Xanthan gum
